# Single-Cell RNA Sequencing Unifies Developmental Programs of Esophageal and Gastric Intestinal Metaplasia

**DOI:** 10.1158/2159-8290.CD-22-0824

**Published:** 2023-03-17

**Authors:** Karol Nowicki-Osuch, Lizhe Zhuang, Tik Shing Cheung, Emily L. Black, Neus Masqué-Soler, Ginny Devonshire, Aisling M. Redmond, Adam Freeman, Massimilliano di Pietro, Nastazja Pilonis, Wladyslaw Januszewicz, Maria O'Donovan, Simon Tavaré, Jacqueline D. Shields, Rebecca C. Fitzgerald

**Affiliations:** 1 Irving Institute for Cancer Dynamics, Columbia University, New York, New York.; 2 New York Genome Center, New York, New York.; 3 Early Cancer Institute, University of Cambridge, Cambridge, United Kingdom.; 4 School of Cancer and Pharmaceutical Sciences, King's College London, London, United Kingdom.; 5 Cancer Research UK Cambridge Institute, University of Cambridge, Cambridge, United Kingdom.; 6 Department of Gastroenterology, Hepatology and Clinical Oncology, Centre of Postgraduate Medical Education, Warsaw, Poland.; 7 Cambridge University Hospital NHS Trust, Cambridge, United Kingdom.

## Abstract

Intestinal metaplasia in the esophagus (Barrett's esophagus IM, or BE-IM) and stomach (GIM) are considered precursors for esophageal and gastric adenocarcinoma, respectively. We hypothesize that BE-IM and GIM follow parallel developmental trajectories in response to differing inflammatory insults. Here, we construct a single-cell RNA-sequencing atlas, supported by protein expression studies, of the entire gastrointestinal tract spanning physiologically normal and pathologic states including gastric metaplasia in the esophagus (E-GM), BE-IM, atrophic gastritis, and GIM. We demonstrate that BE-IM and GIM share molecular features, and individual cells simultaneously possess transcriptional properties of gastric and intestinal epithelia, suggesting phenotypic mosaicism. Transcriptionally E-GM resembles atrophic gastritis; genetically, it is clonal and has a lower mutational burden than BE-IM. Finally, we show that GIM and BE-IM acquire a protumorigenic, activated fibroblast microenvironment. These findings suggest that BE-IM and GIM can be considered molecularly similar entities in adjacent organs, opening the path for shared detection and treatment strategies.

**Significance:**

Our data capture the gradual molecular and phenotypic transition from a gastric to intestinal phenotype (IM) in the esophagus and stomach. Because BE-IM and GIM can predispose to cancer, this new understanding of a common developmental trajectory could pave the way for a more unified approach to detection and treatment.

*See related commentary by Stachler, p. 1291.*

*This article is highlighted in the In This Issue feature, p. 1275*

## Teaser

Barrett's esophagus and gastric intestinal metaplasia are molecularly similar precancerous lesions, with epithelial cells being mosaics of gastric and intestinal phenotypes and stromal cells showing features of cancer-associated fibroblasts.

## INTRODUCTION

Metaplasia is a common phenotypic switch in the upper gastrointestinal (GI) tract that predisposes patients to cancer. The most common metaplasia of the upper GI tract is intestinal metaplasia (IM) characterized by the presence of goblet cells—typical of intestinal tissue. IM can be present in the stomach, defined as gastric IM (GIM), or in the esophagus, where it is a hallmark of Barrett's esophagus (BE). GIM and BE predispose patients to progression to adenocarcinoma of the stomach or esophagus, respectively (1–4). Recent efforts of The Cancer Genome Atlas and the International Cancer Genome Consortium have shown that adenocarcinomas of the stomach and esophagus are molecularly similar diseases sharing genetic, epigenetic, and transcriptional features (5, 6).

In the stomach, the cascade of events is generally triggered by *Helicobacter pylori* infection, which leads physiologically normal gastric cells to become inflamed and develop phenotypic changes termed gastritis. Upon continuous exposure of these cells to exogenous, inflammatory stimuli, atrophic gastritis acquires intestinal features termed GIM, which in a minority of individuals can progress through dysplastic stages to stomach adenocarcinoma (7, 8). Unlike GIM, the initial stages of its development (gastritis) seem to be reversible upon eradication of *H. pylori* (7, 8).

BE is a more proximal precancerous lesion generally occurring in response to chronic exposure to acid and bile refluxate, and in a small proportion of individuals this metaplastic state can progress to esophageal adenocarcinoma (EAC), usually through a dysplastic intermediate stage (1). Although all international societies agree that BE is an acquired metaplastic condition that requires clear identification of the columnar portion arising beyond the gastroesophageal junction (GEJ) characterized by the palisade vessels and gastric folds (9), the precise definition of which cell types constitute a diagnosis of BE is not uniformly agreed upon. The British Society of Gastroenterology defines BE “as an esophagus in which any portion of the normal distal squamous epithelial lining has been replaced by metaplastic columnar epithelium, which is clearly visible endoscopically (≥1 cm) above the GEJ and confirmed histopathologically” (10). This definition includes either: (i) IM: columnar epithelium with intestinal-type goblet cells (BE with IM—BE-IM) or (ii) gastric metaplasia: columnar epithelium without goblet cells (esophagus with gastric metaplasia—E-GM). The U.S. definitions consider only columnar epithelium containing intestinal-type metaplasia as BE (10, 11). These definitions do not consider molecular features for the diagnosis of BE, and they always require endoscopic confirmation of the esophageal origin of BE samples. Moreover, because GIM and BE share histologic features (the presence of goblet cells and specialized columnar cells), the histopathologic distinction between GIM and BE is not possible without information about where the biopsy was taken from (esophagus for E-GM or BE-IM, or stomach for GIM; ref. 12). Furthermore, there is a debate about the relationship between E-GM, BE-IM, and GIM and the degree of malignant potential and hence whether E-GM is clinically significant (9, 13)

Using detailed molecular characterization of healthy and diseased samples of the upper GI tract, we and others have recently shown that BE most likely originates from normal gastric cells that reside within the gastric cardia (4, 14–16). In light of this evidence and considering (i) the histopathologic similarity of BE-IM and GIM, (ii) the histopathologic and molecular similarity between esophageal and stomach adenocarcinomas, and (iii) the precancerous nature of BE-IM and GIM in esophageal and stomach adenocarcinomas development, respectively, one can hypothesize that adenocarcinomas of the stomach and esophagus both arise from gastric cells via BE-IM and GIM with a parallel natural history.

To understand the differences and similarities between these entities, and thus to infer their origin and developmental trajectory, we performed a comprehensive analysis of single-cell RNA sequencing (scRNA-seq) across the entirety of the physiologically normal GI tract and metaplastic conditions of the esophagus and stomach. We characterized the epithelial compartment and the surrounding microenvironmental cells, and the single-cell atlas was supplemented by mutational and protein expression profiling of specific subtypes.

## RESULTS

### The Epithelial Cells of Esophageal and Gastric IM Share Transcriptional Features

**Figure 1.**
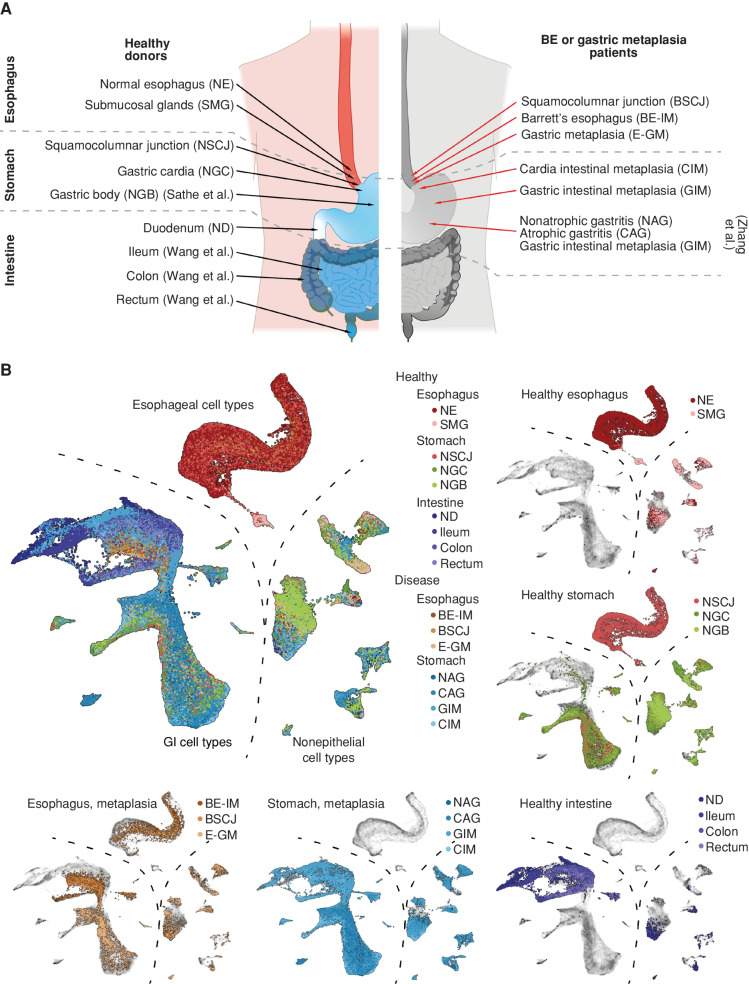
Overview of the scRNA-seq atlas of the GI tract. **A,** Overview of the samples analyzed in the study. For each sample, the approximate location of tissue is indicated. Where indicated, text in brackets denotes the study from which samples originate [Wang et al. (37), Zhang et al. (61), and Sathe et al. (62)]. The remaining samples were collected in the current study or originate from Nowicki-Osuch and Zhuang et al. (4). **B,** UMAP of all high-quality cells used in the study. The main plot shows all tissue types overlay (point order is randomized). The insets show selected tissue types grouped according to their anatomic location and disease state. BSCJ, squamocolumnar junction between NE and BE-IM.

To investigate the similarity between all tissue types, we integrated scRNA-seq datasets from across the entire physiologically normal GI tract (see Methods). Our final dataset included over 146,000 cells spanning the entire length of the human GI tract [normal esophagus (NE), esophageal submucosal glands (SMG), normal squamocolumnar junction (NSCJ), normal gastric cardia (NGC), normal gastric body (NGB), normal duodenum (ND), ileum, colon, and rectum], five normal anatomic sites (esophagus, stomach, small intestine, colon, and rectum), two metaplastic subtypes in the esophagus (BE-IM and E-GM), and metaplastic-related conditions in the stomach [nonatrophic gastritis (NAG), chronic atrophic gastritis (CAG), cardia IM (CIM), and GIM; Fig. 1A; Supplementary Table S1].

**Figure 2.**
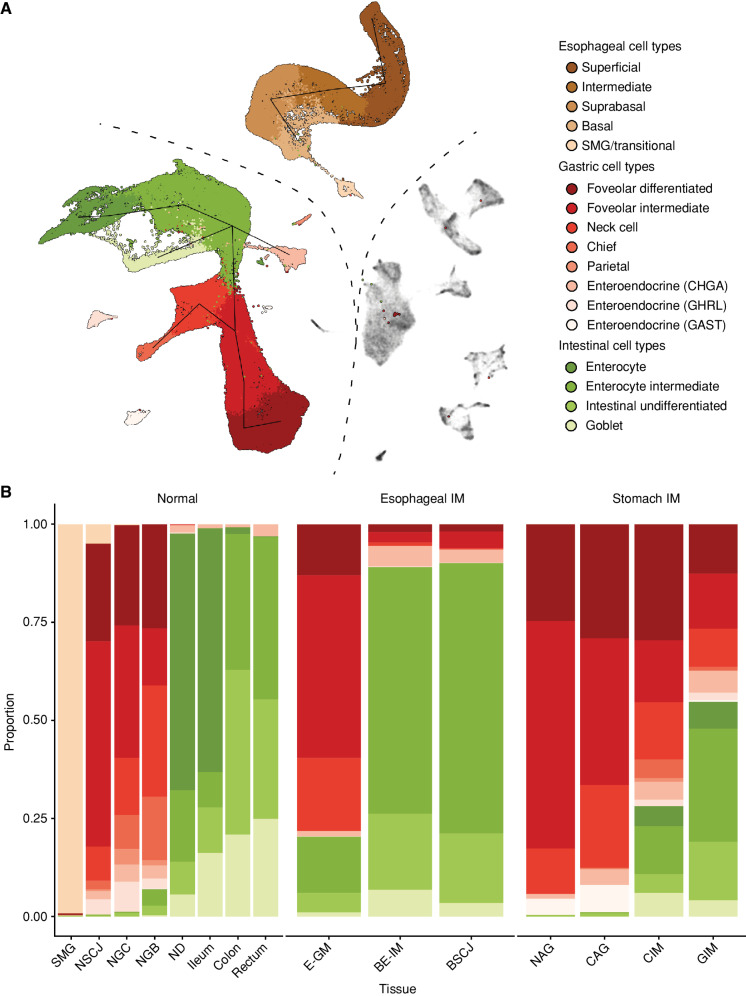
Cell-type distribution across the tissues of the GI tract. **A,** UMAP of all high-quality cells (point order is randomized) used in the study, with the cell types assigned to the epithelial compartment in color. The line indicates a TSCAN-derived trajectory between the clusters of epithelial cells. Nonepithelial cells are in gray. **B,** Stacked bar chart of the proportion of cell types assigned to individual tissue types. The color scale is the same as in **A**. BSCJ, squamocolumnar junction between NE and BE-IM.

We used Uniform Manifold Approximation and Projection (UMAP) for visualization (Methods). After batch correction, clustering, and visualization, the cell types of the GI tract were split into three major classes: (i) immune and stromal cell types, (ii) columnar GI cell types, and (iii) squamous cells and columnar cells of SMG (Fig. 1B). The shared immune and stromal components were used to inform the integration across datasets, as previously described (4). Within each healthy tissue type, we then coarsely assigned cell types to each global cluster using cell markers and information obtained from prior studies (Supplementary Figs. S1–S4; Supplementary Table S2). Next, we used *louvain* clustering to assign phenotypes to cells originating from esophageal metaplasia (E-GM and BE-IM) and stomach metaplasia (NAG, CAG, CIM, GIM; Supplementary Figs. S5 and S6) patients, and we putatively assigned the labels from healthy cells to the most similar cells in the metaplastic conditions (e.g., *MUC6*-expressing cells were assigned a neck-like label). The columnar compartments of both types of metaplasia formed a continuum and mapped to the gastric and intestinal phenotypes. Furthermore, in line with our previous BE study and the established literature associated with stomach IM, we did not observe the similarity between all IM phenotypes and SMG nor NE epithelial cell types (Fig. 2A). The similarity between the phenotypes was independently confirmed using the TSCAN algorithm (Fig. 2A).

Further, we observed a striking similarity between the developmental stages of esophageal and gastric metaplasia. In comparison with NGC and NGB, E-GM was characterized by the presence of gastric neck-like (*MUC6*-expressing cells), foveolar-like cells, and chromogranin A (*CHGA*)–expressing endocrine cells and the absence of the parietal, chief, and Ghrelin (*GHRL*)-expressing endocrine cells. In line with their immature intestinal phenotypes, there were few (<1%) goblet cells, and the remaining columnar cells did not show intestinal phenotypes (Fig. 2B; Supplementary Fig. S5). NAG and CAG, although residing in the stomach, displayed the same pattern of cell types (Fig. 2B; Supplementary Fig. S6). The BE-IM and stomach intestinal metaplasia (GIM and CIM) phenotypes were demarcated by an abundance of goblet cells (the histologic hallmark of these conditions) and the development of an intestinal, enterocyte-like phenotype in the remaining columnar cells (Fig. 2B; Supplementary Figs. S5 and S6). We observed a higher proportion of gastric-like cell populations (neck and foveolar) in the GIM and CIM specimens than in BE samples. This is likely explained by the presence of adjacent normal gastric cells in these specimens. Finally, the GIM/CIM samples contained fully differentiated enterocyte-like cells that were absent from BE specimens.

### Individual Columnar Cells of Esophageal and Gastric IM Are a Mosaic of Intestinal Enterocytes and Gastric Foveolar Cells

**Figure 3.**
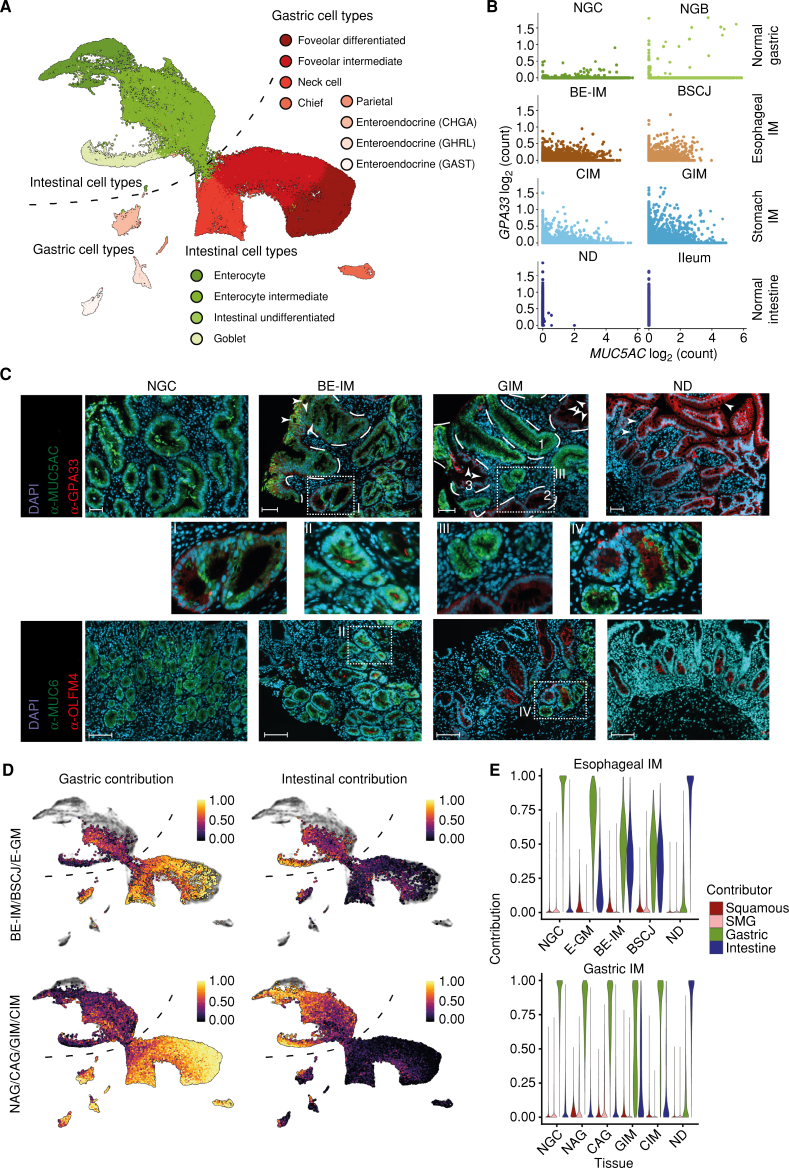
Columnar cells of the stomach and esophageal IM have mosaic gastric and intestinal phenotypes. **A,** UMAP of columnar cells isolated from samples of NSCJ, NGC, NGB, ND, ileum, colon, rectum, BE, gastric metaplasia of esophagus, squamocolumnar junction between NE and BE, CIM, GIM, nonchronic atrophic gastritis, and chronic atrophic gastritis with color denoting cell types. The cell types were assigned using cells from normal tissue types. **B,** Scatter plot of log-normalized expression of intestinal (*GPA33*) and gastric (*MUC5AC*) markers in the selected tissue types. **C,** Coimmunofluorescent staining of the esophagus with BE with BE-IM and GIM shows coexpression of intestinal and gastric markers in both types of IM using lineage markers MUC5AC (gastric) and GPA33 (intestinal), and progenitor markers MUC6 (gastric) and OLFM4 (intestinal). White arrowheads denote selected goblet cells; dashed white lines indicate selected BE-IM and GIM crypts, for GIM samples: 1, a crypt with mixed gastric and intestinal phenotype; 2, a crypt with cells showing mosaic phenotype; 3, a crypt with features of complete IM; scale bar, 100 μm. See supplementary Figs. S8 and S9 for ND and NGC. Images are representative of 12 patients (NGC = 2, ND = 2, BE-IM = 4, and GIM = 4). **D,** UMAP with MuSiC-derived contribution of gastric and intestinal phenotypes to individual cells of the esophageal IM (top) and stomach IM (bottom). **E,** Violin plots of squamous, SMG, gastric, or intestinal phenotype contribution to cells from NGC, E-GM, BE-IM, BSCJ, NAG, CAG, GIM, CIM, and ND samples.

Our observations about the cellular composition of gastric and esophageal IM are in line with the existing literature that suggests IM tissue is a mosaic of gastric and intestinal cell types (17, 18). However, the analysis of the expression pattern of individual genes considered to be the canonical marker of gastric (e.g., *MUC5AC*) or intestinal (e.g., *GPA33*) tissues revealed that the individual columnar cells of BE-IM, CIM, and GIM can express both intestinal and gastric markers simultaneously (Fig. 3A and B; Supplementary Fig. S7A and S7B). Coimmunofluorescence staining of patient samples of BE-IM and GIM confirmed this mosaic phenotype (Fig. 3C). In particular, BE-IM and GIM showed a gastric/intestinal mosaic phenotype as defined by the coexpression of markers of differentiated mucous cells (MUC5AC) and differentiated enterocytes (GPA33), as well as gastric (MUC6) and intestinal (OLFM4) progenitor cells, respectively (Fig. 3B and C; Supplementary Figs. S8 and S9; ref. 19). This pattern was absent in the normal intestine or gastric samples (Supplementary Figs. S7B, S8, and S9). We hypothesized that the cells of BE-IM, CIM, and GIM samples could form a single intermediate (mosaic) phenotype between foveolar and enterocyte phenotypes.

To assess these observations quantitively, we identified the phenotypes of esophageal and stomach IM using existing cell identification tools. First, we performed unsupervised annotation of cell phenotypes using SingleR (20). SingleR trains its algorithms using single-cell profiles of known cell types and subsequently identifies the phenotype of the unknown cells. We trained the tool using our well-annotated cellular phenotypes of the epithelial cells originating from healthy tissue types: gastric cardia, gastric body, duodenum, ileum, colon, and rectum. A benchmark comparison of known cell types with predicted cell types showed strong concordance between the manual and automatic annotation (Supplementary Fig. S10). An extension of this approach to the NSCJ cells further confirmed the accuracy of predictions (for columnar compartments of NSCJ; Supplementary Fig. S10). When we applied the method to esophageal and gastric IM cells, the identities of nonepithelial (stromal and immune), endocrine, and goblet cells were clearly assigned to the corresponding phenotypes (Supplementary Fig. S11). However, SingleR-derived annotation of the remaining columnar cells of BE-IM, CIM, and GIM was inconsistent with *louvain* clustering (Supplementary Fig. S11). These cells showed characteristics of both gastric and intestinal cell phenotypes. This was especially apparent for cells that were classified as enterocytes using the *louvain* method. Despite the strong expression of intestinal gene markers, SingleR classified them as foveolar-like cells (Supplementary Fig. S11).

Because SingleR takes the entire transcriptome into consideration, we reasoned that the columnar cells of esophageal and gastric IM might have a unique mosaic phenotype of intestinal and gastric tissue types (with gastric phenotype dominating SingleR analysis). To quantify the contribution of the intestinal phenotype to the identity of columnar cells of esophageal and stomach IM, we assumed that each phenotypical component can be deconvoluted from the transcriptional profiles of individual cells. We performed phenotypical deconvolution using MuSiC (21). We trained the algorithm using phenotypes of epithelial cells of NE, SMG, NGC, NGB, ND, ileum, and colon, and we calculated the esophagus, gastric, intestinal, and colon (EGIC) score. This score accurately recapitulated the identity of healthy GI tract cell types (Supplementary Figs. S12A–S12F and S13A and S13B). It further showed that the columnar cells of BE-IM and stomach IM cells have a mixed phenotype of gastric and intestinal cell types (Fig. 3D). In scRNA-seq analysis, this mixed phenotype was predominantly observed in undifferentiated, stem-like cells and transitional (intermediate) cell types, whereas fully differentiated cells showed predominantly either gastric or intestinal specificity (Supplementary Figs. S14A and S14B, and S15A and S15B). The distinction between cells that possess partial and complete intestinal properties is consistent with histologically distinguishable stages of IM development—complete and incomplete IM (18, 22). Similar to our earlier analysis, the contribution of NE and SMG phenotypes to the EGIC score of BE-IM and stomach IM cells was minimal (Supplementary Fig. S16A–S16D). Of note, we saw the minimal contribution of the colonic signal to the BE-IM and stomach IM phenotypes (Supplementary Fig. S16E and S16F), suggesting that both types of IM develop by the acquisition of specific, small intestinal phenotype by gastric cells.

When comparing esophageal IM with (BE-IM) and without (E-GM) goblet cells, the contribution of the intestinal features increased significantly in IM cells, as expected (Fig. 3E). Similarly, we observed a stepwise acquisition of an intestinal phenotype in the NAG→CAG→GIM/CIM progression that was also associated with the gradual loss of gastric properties by these cells (Fig. 3E).

We further validated the EGIC score using a recently published independent cohort of scRNA-seq from gastric cancer samples (23). After data integration, we computationally isolated columnar and cancer cells (Supplementary Fig. S17A–S17D). Similar to our earlier analysis, cancer cells were most closely related to columnar cells (Supplementary Fig. S17A and S17B). The EGIC score for the columnar cells present in the normal biopsies adjacent to gastric cancer (NGB adjacent; Supplementary Fig. S17D) was mainly composed of signals originating from the gastric component. In the case of cancer cells, we also observed the acquisition of intestinal phenotypes; however, we did not see a strong intestinal contribution to the EGIC score of cancer cells (Supplementary Fig. S17D), unlike BE-IM, GIM, and CIM cells.

Independent, pseudotime-based analysis using *monocle3* (24, 25) further showed that esophageal and gastric IM cells have intermediate phenotypes between gastric and intestinal phenotypes. Similar to our deconvolution analysis, E-GM resided at an earlier pseudotime timeline of BE-IM phenotype development, and this trajectory was recapitulated in the NAG→CAG→GIM/CIM progression (Supplementary Fig. S18A–S18F). Additionally, in line with our bulk analysis of esophageal cancer (26) and the existing literature associated with gastric cancer, we observed that cancer cells derived from gastric cancer samples are not at the completely intestinalized termini of the trajectory (Supplementary Fig. S18F and S18G), suggesting that (i) fully intestinalized cells can revert to a less differentiated phenotype and progress to cancer or (ii) complete intestinalization of GIM is not associated with cancer progression (27).

### Gastric Metaplasia in the Esophagus Shares Features with Atrophic Gastritis

**Figure 4.**
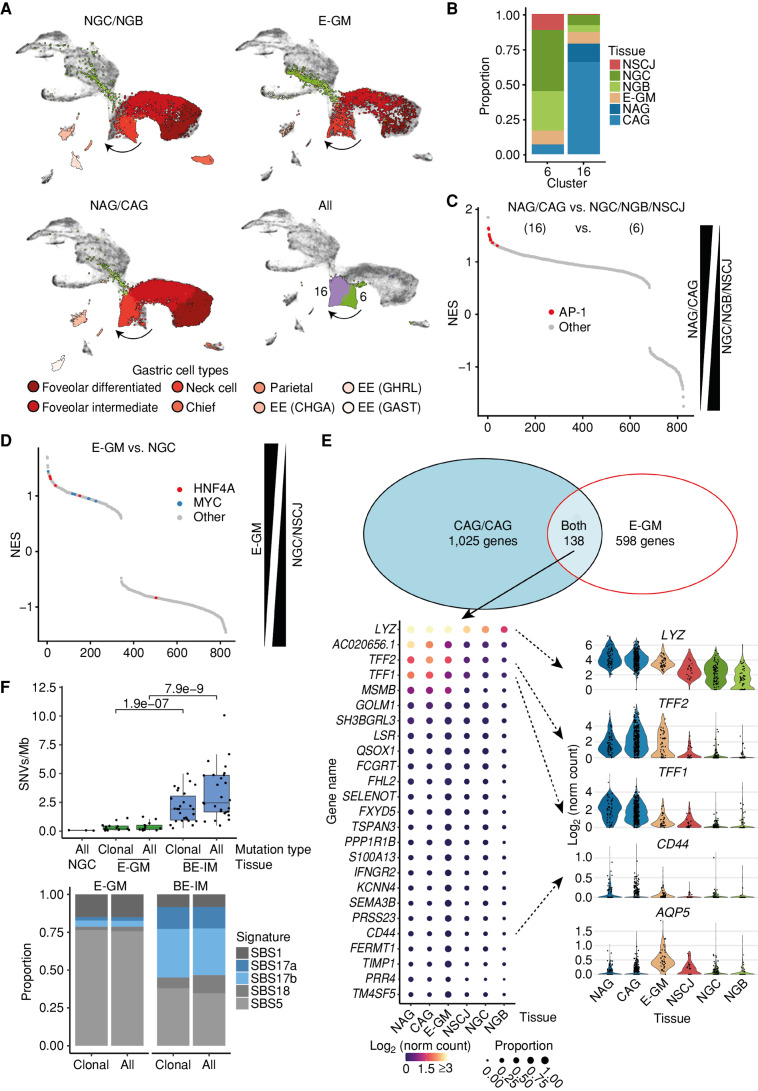
E-GM and atrophic gastritis share early features of developing IM. **A,** UMAP of columnar cells of gastric cell types (NGC and NGB, top left), E-GM (top right), and atrophic gastritis (NAG and CAG, bottom left) with cell-type annotation highlighted. Bottom right, UMAP with clusters 6 and 16 (identified during reclustering of columnar cells and overlapping gastric neck cells) highlighted. EE, enteroendocrine. **B,** Stacked bar chart of tissue contribution to the cells assigned to clusters 6 and 16. **C,** Gene set enrichment analysis (GSEA) using the C3 gene set database of differentially expressed genes between neck-like cells from E-GM and NGC samples. HNF4A- and MYC-related pathways are highlighted. The differential analysis was done between E-GM cells and NGC + NSCJ neck-like cells with each patient treated as an individual replicate. NES, normalized enrichment score. **D,** GSEA using the C3 gene set database of differentially expressed genes between NAG/CAG and NGC/NGB/NSCJ sample neck-like cells. AP-1–related pathways are highlighted. The differential analysis was done between cluster 16 cells (including NAG, CAG, and E-GM cells) and cluster 6 (including NGC + NGB + NSCJ neck-like cells) with each patient treated as an individual replicate. **E,** Venn diagram demonstrating the overlap between genes enriched in the comparison of atrophic gastritis (NAG and CAG) and normal gastric samples or E-GM and normal gastric samples. Bottom left: bubble plot of top 25 expressed genes shared between the comparison. Bottom right, violin plots of selected genes. **F,** WGS-based analysis of E-GM and BE-IM. Top, mutational burden [single-nucleotide variants/megabase (SNV/Mb)] of NGC, E-GM, and BE-IM samples. Bottom, distribution of COSMIC SNV signatures in the E-GM and BE-IM samples.

Next, we focused our attention on the diseased cell and molecular phenotypes residing in the esophagus and stomach without IM (9, 28). First, we observed that E-GM and NAG/CAG samples share similar features. Both diseases were characterized by the absence of the parietal, chief, and *GHRL*-expressing endocrine cells and the acquisition of a weak intestinal phenotype in the remaining columnar cells (Fig. 2B; Supplementary Figs. S5 and S6). We searched for specific markers of these early lesions. We noticed a difference between the neck-like cells of E-GM/NAG/CAG and normal gastric neck-like cells (Fig. 4A), which we confirmed by reclustering analysis of the columnar cells (Supplementary Fig. S19A). These cells formed clusters 16 and 6 (Fig. 4A; Supplementary Table S3). Cluster 6 was mainly composed of normal cells from NGC, NSCJ, and NGB (Fig. 4B; Supplementary Fig. S19B). We further noticed a difference between NGC cells collected from healthy donors and NGC cells collected from samples adjacent to IM. The majority (>95%) of neck-like cells from NGC samples collected from disease-free organ donors were in cluster 6 (Supplementary Fig. S19B). Conversely, only ∼60% of NGC adjacent to diseases were in cluster 6 (Supplementary Fig. S19B). In the disease states, the majority of E-GM (∼70%), NAG (>95%), and CAG (>95%) cells were in cluster 16 (Fig. 4B; Supplementary Fig. S19B). Differential expression (DE) gene analysis and gene set enrichment analysis between clusters 16 (NAG, CAG) and 6 (normal) showed upregulation of genes controlled by AP-1 (a dimer of JUN and FOS transcription factors; Fig. 4C). To identify genes that were specific for GM development, we directly compared E-GM neck-like cells with NGC and NSCJ neck-like cells. In line with our previous observation, we noticed that the transcriptional program was dominated by MYC and HNF4A transcription factors, which are the main drivers of BE-IM development from normal gastric cells (4), further supporting that these genes play key roles in the early stages of its development (Fig. 4D). Finally, we identified 138 genes that were upregulated in both stomach and esophageal diseases (Fig. 4E; Supplementary Table S4). These genes included recently identified mouse markers (*CD44*, *TFF2*, and *AQP5*) of spasmolytic polypeptide-expressing metaplasia (SPEM; refs. 29, 30).

To shed further light onto the relationship between E-GM and BE-IM, we performed whole-genome sequencing (WGS) of clinically confirmed E-GM samples to complement our previous BE-IM data (26). This showed that the mutational burden was substantially lower in E-GM compared with BE-IM and was independent of the clonal status of mutations (Fig. 4F). On average, we observed 1,280 point mutations per sample (Supplementary Table S5), and the mutation burden was independent of age or sequencing coverage. The signature profile, as defined by the COSMIC database, showed an aging profile with very little evidence of SBS17 typically observed in BE-IM and EAC (26, 31). None of the E-GM samples had substantial chromosomal aberrations (ploidy 1.9–2.0; Supplementary Fig. S20A), and no mutations in common driver genes were observed in BE-IM and EAC except for one sample with a point mutation in the *SMARCA4* gene. Next, we evaluated the penetrance of individual mutations within E-GM samples. For the majority (13 out of 14) of samples, the variant allele frequency (VAF) followed a right-side skewed distribution with an average mode of ∼0.1. We did not observe a tail of high-penetrance (VAF >0.25) mutations in E-GM (Supplementary Fig. S20B), in contrast to normal gastric samples. We and others have previously shown that these mutations most likely arise during embryonic development and are present in all epithelial cells (4, 32). We can then tentatively infer that E-GM mutations follow a clonal distribution; however, it should be noted that normal gastric tissue was used as a reference sample during mutation calling, potentially obstructing the detection of these mutations (Supplementary Fig. S20B). Overall, the DNA sequencing data show that GM has a mutation profile in line with its low malignant risk (33).

### An Expansion of Stromal Cells Involved in Matrix Deposition Characterizes Developent of Stomach and Esophageal IM

Having established the developmental expression pattern of epithelial cells present in the gastric and esophageal IM, we next turned our attention to the nonepithelial cells present in our samples in view of the increasing data suggesting a contribution of the microenvironment in carcinogenesis (34–36). Due to sample processing performed by Wang and colleagues (37), very few immune and stromal cells were identified in this dataset. As a result, these samples were excluded from the subsequent analysis. Across the analyzed samples, we did not identify significant quantitative and qualitative changes in cell populations of endothelial cells (Supplementary Fig. S21A–S21C) and T-cell populations (Supplementary Fig. S22A–S22C). We observed two distinct types of natural killer (NK) cells: intestinal marked by granzyme A (*GZMA*) expression and gastric marked by granzyme B (*GZMB*) expression (Supplementary Fig. S22A and S22C). All IM samples—that is, BE-IM and GIM—were predominantly marked by the presence of the gastric NK cells, further suggesting their similarity. We did not see differences between B cells present in all samples (Supplementary Fig. S23A–S23D); however, we observed some isotype switching in the plasma cells. As expected, we observed that IgA-producing cells are the dominant isotype of plasma cells in the GI tract. IgA1/IgKappa is the dominant type in all cases except for the GIM and CAG from the Zhang cohort (ref. 61; Supplementary Fig. S23D), in which IgA2/IgKappa was the dominant type. The difference between Cambridge CIM/GIM and Zhang GIM samples was not explained by *H. pylori* status or technical differences (Supplementary Fig. S23C; NAG samples from Zhang's study had similar plasma cell populations to Cambridge CIM/GIM). Finally, with the exception of a slight increase in the ratio of monocytes in the BE, we did not observe marked differences between the population of myeloid cells (Supplementary Fig. S24A–S24C).

**Figure 5.**
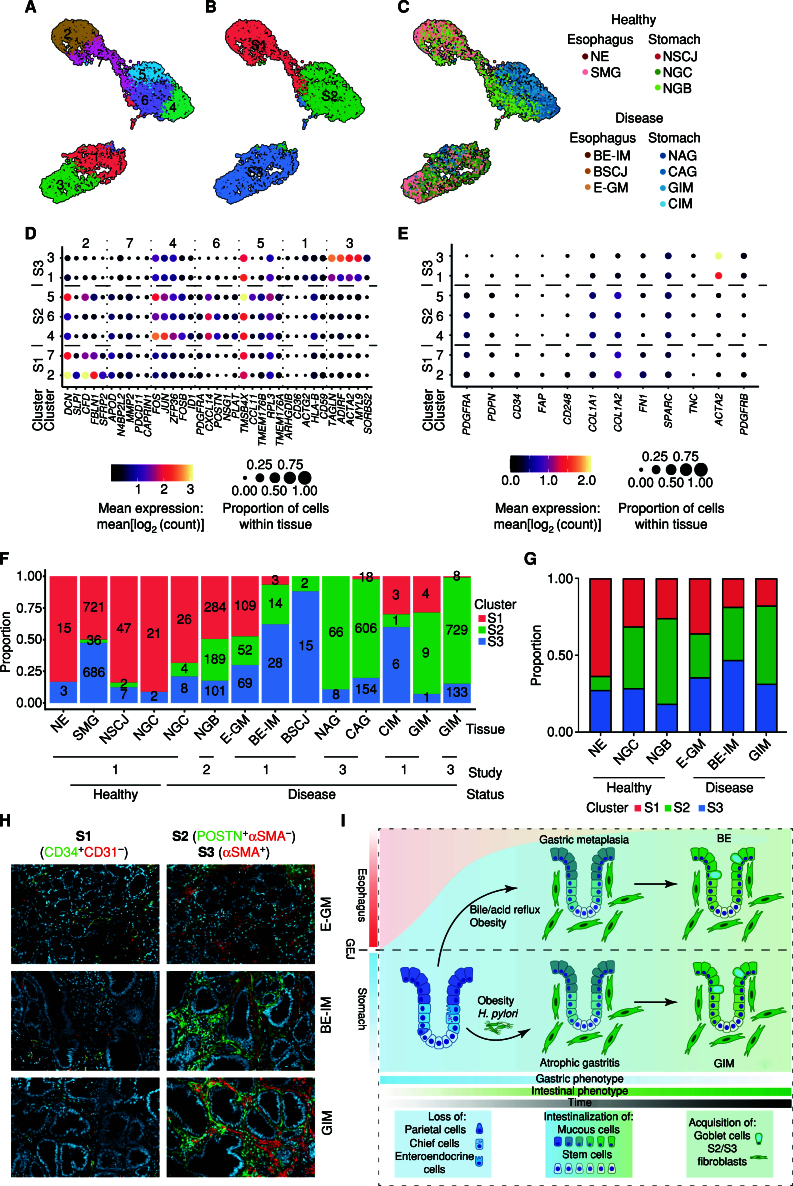
Gastric and esophageal IM are enriched for extracellular matrix–depositing subtypes of fibroblasts. **A,** UMAP of fibroblast-like cells (cluster 13 in Supplementary Fig. S1) with fibroblast-specific clusters highlighted. **B,** UMAP of fibroblast-like cells (cluster 13 in Supplementary Fig. S1) with manually annotated cell types derived from Davidson et al. (38). **C,** UMAP of fibroblast-like cells (cluster 13 in Supplementary Fig. S1) with the contribution of individual tissue type highlighted. **D,** Bubble plot of top five genes identified in the differential analysis between the fibroblast-specific cell clusters. **E,** Bubble plot of genes identified as markers of early fibroblast development by Davidson et al. (38). **F,** Contribution of cell types derived from Davidson et al. (38) to cell counts from individual tissue types. The tissues originate from three studies: (i) Nowicki-Osuch and Zhuang et al. (4) and this study, (ii) Sathe et al. (62), and (iii) Zhang et al. (61). For tissue for which patient status could be identified, the tissue was split into healthy and disease states. NGB samples are adjacent to gastric cancer samples. **G,** Proportion of each subtype in total S1/S2/S3 fibroblasts derived from IF staining of E-GM, BE-IM, and GIM. **H,** Representative IF staining of E-GM, BE-IM, and GIM of S1 (CD34^+^CD31^−^), S2 (POSTN^+^aSMA^−^), and S3 (aSMA^+^) fibroblasts. **I,** Schematic summary of unified developmental trajectories between BE-IM and GIM.

Next, we focused our attention on the stromal compartment of our scRNA-seq data. Reclustering of stomal cells (initially characterized by the expression of CALD1, FBLN1, and COL6A2) split these cells into seven clusters (Fig. 5A; Supplementary Table S6), with marker genes showing three major classes of cell types across all tissue types (Fig. 5B–D). Cross-comparison with a recently published mouse melanoma model of cancer development (38) suggested that the major three classes of cell types resemble the following: S1 early immune (clusters 2 and 7); S2 desmoplastic/extracellular matrix–depositing (clusters 4–6); and S3 contractile (clusters 1 and 3) fibroblast phenotypes (Fig. 5B and E; Supplementary Table S6). Similar to the mouse melanoma model data, the S1 population (PDGFRA^hi^ PDPN^hi^ CD34^hi^) was characterized by the expression of genes involved in the regulation of immune cell recruitment (cytokines: CXCL12; complement factors: CFD, C3, C1S, CFH). The S2 cell population (PDGFRA^hi^ PDPN^hi^ CD34^lo^) was characterized by the expression of extracellular matrix components (CXCL14, POSTN, COL6A1, and COL6A2). Furthermore, one of the subclusters of the S2 population (cluster 4) was characterized by transcription of immediate early genes (FOS, JUN, and FOSB). This family of transcription factors has recently been associated with the activation of angiogenesis in breast cancer models (39). The S3 population expressed markers of contractile fibroblasts (ACTA2, MYL9, and ACTG2). *Monocle3*-based pseudotime analysis recapitulated links between stromal cell populations and highlighted a gradual increase in the transcriptional signal associated with S2/S3 development as the cells dedifferentiated from S1 (Supplementary Fig. S25A–S25F).

The comparison of different tissue types showed expansion of S2/S3 populations in the samples collected from the IM of the stomach (Fig. 5C and F). This observation was independent of the anatomic site and specific research study, even though we observed a different number of sequenced cells per individual sample (Fig. 5F). In the case of esophageal IM (BE-IM), as observed for GIM, we observed a decrease in the proportion of the S1 cell population that was counteracted by an expansion of S2/S3 populations, and this was confirmed using immunofluorescent (IF) microscopy (Fig. 5G and 5H; Supplementary Figs. S26 and S27A–S27C). In line with our scRNA-seq data, we saw the expansion of S2/S3 populations, as well as a decrease in the S1 population in both IM types (BE-IM and GIM), and this trend was also observed in the E-GM, making it the first clear marker of metaplastic change in the esophagus. To shed further light on this, we also compared two types of E-GM: (i) E-GM biopsies collected from patients without any IM detected in other biopsies collected during the same procedure and (ii) BE-IM “Gob-” samples collected from BE-IM patients who did not have histologically detectable goblet cells within that specific biopsy. Interestingly, BE-IM Gob- showed a significantly lower composition of S1 fibroblasts when compared with E-GM, which could be potentially exploited as a BE-IM marker that does not rely on IM goblet cell features because the assessment of goblet cells is prone to sampling bias (Supplementary Fig. S28A–S28C).

## DISCUSSION

Our scRNA-seq data suggest that BE-IM and GIM share transcriptional similarities that make them phenotypically indistinguishable. First, in line with their histopathologic characterization, both diseases are defined by the presence of clearly identifiable goblet cells that share transcriptional features with goblet cells of the small and large intestines (40). The remaining columnar cells of BE-IM and GIM are characterized by the presence of columnar phenotypes that harbor different levels of intestinal development (Fig. 5I). Historically, both diseases have been characterized as mosaic tissue containing two cell phenotypes (intestinal and gastric), and recent histologic studies identified a wide diversity of cell types present within individual BE-IM crypts (17, 18). Our scRNA-seq data demonstrate, for the first time, that not only the tissue but also each individual epithelial cell is a mosaic of two phenotypes: gastric and intestinal. These cells exist on a continuum with some cells retaining the gastric phenotype and some acquiring full intestinal enterocyte properties. Each gastric columnar cell type (including putative isthmus/neck stem cells) that is retained during IM development gradually acquires an intestinal phenotype. Transdifferentiation can be defined as an irreversible switch of one type of already differentiated cell type to another type of normal differentiated cell type (41, 42), whereas transcommitment can be defined as a shift of developmental trajectory of a stem cell from normal to abnormal differentiation pattern (43). The simultaneous existence of chimeric (GI) stem cells and differentiated columnar cells in both gastric and esophageal IM does not neatly fit into either of these definitions. To the best of our knowledge, this is the first time that an intermediate state between two normal tissue phenotypes has been captured in pseudo real time in human data.

The partial and complete acquisition of intestinal phenotype by gastric and esophageal IM is in line with the histopathologic classification of these conditions that include incomplete and complete IM (18, 22). Due to the nature of scRNA-seq that does not retain the tissue architecture, we were not able to directly correlate the histologically defined “completeness” of IM cells with their phenotypes. However, in line with the literature, we can speculate that complete IM also has a high intestinal EGIC score. We further observed that gastric cancer cells have a mixed EGIC score in line with the literature suggesting that incomplete IM is the precursor of gastric adenocarcinoma (18, 44).

Our recent comprehensive multimodal assessment of BE-IM origin identified gastric cells as a source of BE-IM (4). The striking phenotypic similarity between BE-IM and GIM further supports this conclusion. The gastric origin of GIM has never been questioned (45). Our data suggest that both diseases do not share phenotypic similarities with NE, SMG (46), or specialized transitional epithelium at NSCJ (47). Given the general rule of parsimony in biology, it seems highly unlikely that two phenotypically similar diseases that eventually lead to molecularly similar tumors would arise from different progenitor cells.

Recent comprehensive molecular characterization of esophageal and stomach adenocarcinomas suggests that these diseases are identical on genetic, epigenetic, and transcriptional levels (5, 6). Our conclusion that BE-IM and GIM are also phenotypically similar supports the notion that as a result of distinct exogenous triggers of reflux and infection, respectively, both cancers follow similar molecular trajectories of progression wherein normal gastric cells acquire an intestinal phenotype and a small proportion (0.3% per year) of IM develops genetic aberrations consistent with progression to adenocarcinoma (48–50). Currently, clinical data identify GIM and BE-IM as cancer risk factors; however, focal IM at the cardia without an endoscopically visible columnar segment in the esophagus is more controversial with regard to its risk of cancer progression (51, 52), and this could reflect the quantity of IM present.

Our data further show that E-GM has some phenotypic features of atrophic gastritis. First, we observed a clear loss of chief and parietal cell types. Second, within the stem-like cells of E-GM and NAG/CAG samples, we observed the early phenotypic change that allows for the distinction of these cells from gastric cardia stem-like cells. Of note, the stem-like cells of NAG/CAG (MUC6^+^ neck cells) expressed recently identified marker genes (*CD44*, *AQP5*, *TFF2*, and *LYZ*) of SPEM (29, 30). SPEM has been proposed as an early developmental stage of gastric cancer; however, its clinical role is poorly understood (53). Our data suggest that the SPEM-like phenotype might also exist in E-GM mucous neck-like cells. We further observed that chief and GHRL-expressing endocrine cell phenotypes, mainly present in cells derived from samples collected from the proximal (NSCJ) and distal (2 cm below squamocolumnar junction—NGC) cardia locations, were absent in E-GM tissue, potentially allowing for its distinction and simplified diagnosis of this condition that is independent of endoscopic characterization of sample origin. Considerable debate exists about the nature of cardia epithelium and its disease status (54–56); hence, we cannot rule out that E-GM has properties of cardia mucosa as defined by Chandrasoma and colleagues (57). The development of high-resolution spatial transcriptomics coupled with targeted sample collection should allow for detailed studies in the future. The lower mutational burden and benign profile observed in E-GM when compared with BE-IM is in keeping with its more indolent behavior, though it should also be noted that columnar segments without IM also tend to be shorter, which may also influence their malignant potential.

Finally, our scRNA-seq analysis identified S2/S3 fibroblasts as novel populations of fibroblasts that share features with the well-described cancer-associated fibroblast (CAF) population. However, unlike CAFs, these cells are already present in the nondysplastic stages of cancer development. In line with the previously described early development of CAFs in cancer models (38), we hypothesize that the emergence of S2/S3 fibroblasts in BE-IM and GIM creates a protumorigenic environment that facilitates the development of future cancer cells, and future studies would be interesting to address their role in disease progression.

A limitation of the study is the possible loss of some of the cell types during single-cell library preparation. Although we identified all known cell types within our atlas, we noticed that some of the cell types were affected by library preparation methods; for example, in comparison with epithelial cells, we observed fewer immune cell types than expected. In line with the previous scRNA-seq (58), we focused our analysis on quantitative comparison within individual immune cell subtypes. The conclusions drawn have been robustly confirmed using independent cohorts from previously published studies. However, due to this technical limitation, we were not able to identify previously observed changes associated with BE-IM immune phenotypes (59, 60). Second, future studies are needed to further understand epigenetic changes (e.g., using single-cell Assay for Transposase-Accessible Chromatin using sequencing) within individual cell populations. Finally, due to the nature of scRNA-seq, the spatial relationship between cell types has been lost. The technical advances in spatial transcriptomics will allow for future studies aiming to identify how the changes of cell phenotypes during disease progression affect the interaction of stromal and epithelial environments. Similar approaches will allow for detailed characterization of the heterogeneity within the gastric and esophageal IM, including differences between complete and incomplete IM previously observed histologically.

Taken together, we conclude that IM of the stomach and IM of the esophagus share the same epithelial phenotype and stromal microenvironment. Taken together with our recent identification of a gastric origin for BE-IM, and the molecular continuum described for esophageal and stomach adenocarcinomas, this suggests that BE-IM and GIM are single disease entities that evolve in response to an inflammatory trigger—be that a pathogen or a chemical injury from refluxate. This finding suggests that a more unified approach could be taken for the early detection of precancerous lesions at either side of the GEJ as well as joint enrollment into clinical trials of patients with esophageal and proximal gastric adenocarcinoma.

## METHODS

### Patient and Donor Information

Endoscopic samples were collected at the Cambridge University Hospitals NHS Trust (Addenbrooke's Hospital) from nondysplastic patients with at least 1 cm of BE or esophageal gastric metaplasia or from GIM of gastric body or cardia (REC 01/149). Healthy, endoscopic reference samples were collected from patients without endoscopic or histologic evidence of BE (REC 01/149). Additionally, previously published (4) healthy samples were collected from deceased transplant organ donors (REC 15/EE/0152). Additional GIM scRNA-seq data were obtained from a previously published study (61). Finally, healthy control scRNA-seq data of NGB and intestine were obtained from (37, 62). Supplementary Table S1 contains details of individual samples collected for the study, including patients’ dysplasia/cancer progression status. The study was approved by the Institutional Ethics Committees, conducted in accordance with the Declaration of Helsinki, and written informed consent was obtained from all subjects.

### Sample Overview

To perform this comparative analysis, we used our previously published scRNA-seq data from ND, NGC, NE, NSCJ, SMG, BE-IM, and the squamocolumnar junction between BE-IM and NE (BSCJ). We further extended the analysis to include novel biopsy samples collected from BE-GM, CIM, and GIM. Finally, we also included previously published scRNA-seq data from NAG, CAG, and GIM samples obtained by Zhang and colleagues (61), scRNA-seq data from NGB samples published by Sathe and colleagues (62), and scRNA-seq data obtained from the normal rectum, colon, and ileum by Wang and colleagues (37). Our final dataset included over 146,000 cells spanning the entire length of the human GI tract (NE, SMG, NSCJ, NGC, NGB, ND, ileum, colon, and rectum), five normal anatomic sites (esophagus, stomach, small intestine, colon, and rectum) and all known stages of development for IM of esophagus (BE-GM and BE-IM) and stomach (NAG, CAG, GIM, CIM; Fig. 1A). Additional cancer samples were obtained from the recently published study by Kumar and colleagues (23). Only primary tumor and adjacent normal samples were used in the analysis.

### Sample Collection

As this study is exploratory in nature, no formal blinding, randomization, or power analysis was performed. Subject demographics (age and weight) are provided in Supplementary Table S1. For each condition, samples were collected from at least three patients (replicates) with two biopsies per site. Patients’ gender was not used as a biological variable. No specific exclusion criteria were used for patients. The endoscopic samples were collected using a standard endoscopic technique by highly experienced endoscopists (M. di Pietro, W. Januszewicz, and N. Pilonis). NE samples were collected at least 2 cm above the squamocolumnar junction and BE samples from at least 2 cm below the BSCJ but from a clearly defined esophageal region. NGC samples of non-BE patients were collected at least 2 cm below NSCJ, NGC samples of BE patients were collected 2 cm below the anatomically defined GEJ, and NGC samples of CIM patients were collected endoscopically from NGC (2 cm below the anatomically defined GEJ). B-SCJ samples were collected directly at the BE-NE squamocolumnar junction and NSCJ samples were collected directly at the NE-NGC squamocolumnar junction. ND samples were collected endoscopically from the second part of the duodenum. All samples were sequenced and further processed for read alignment and quality control (see Supplementary Table S1).

### 10x Genomics scRNA-seq

Samples were finely minced with a scalpel and digested with 0.25% Trypsin-EDTA (Thermo Fisher, #25200056) for 30 minutes at 37°C with occasional agitation. The digestion was terminated by additional RPMI (Thermo Fisher, #R8758) supplemented with 10% fetal bovine serum (Thermo Fisher, #16000044), and the cell suspension was filtered with a 70-μm mesh. A cell pellet was collected following 5 minutes of centrifugation at 300 × *g*. Red blood cells were removed with red blood cell lysis buffer (BioVision, #5830-100). The cells were resuspended in PBS + 0.04% BSA (Sigma-Aldrich, #SRE0036) and counted, and 3,000 to 5,000 cells were loaded into the chromium controller (10x Genomics) following the manufacturer's instructions and processed using version 3 of the 3′ scRNA-seq protocol (Supplementary Table S1).

### WGS and Analysis

Each sample was snap-frozen and embedded in OCT. Hematoxylin and eosin (H&E) staining was performed for each specimen, and stained slides are available on request. WGS was performed as previously described (63). Briefly, RNA and genomic DNA were extracted from whole tissue samples using the Qiagen AllPrep Mini Kit (cat no. 80284). Libraries were prepared using the Illumina PCR Free Tagmentation kit with matching IDT unique dual indexes (UDI) and protocols (cat no. 20041795 and 20027213), with inputs ranging from 50 to 1500 ng of total DNA. Libraries were diluted 1:10,000 for quantification on a Roche LC480 II using the Kapa universal Illumina qPCR kit (cat no. 07960140001), and pooling was balanced dependent on the coverage required per sample. Paired-end sequencing was carried out for 14 esophageal gastric metaplasia biopsies and matched NGC biopsies using Illumina's NovaSeq 6000 platform. Minimum average post-filtering depths of 58× for gastric metaplasia and 36× for matched normal samples were achieved.

Reads were mapped to the human reference genome (GRCh37) using Burrows–Wheeler alignment (BWA-mem) 0.7.17 (RRID:SCR_010910; ref. 64), and duplicates were marked using Picard 2.9.5. Somatic mutations and indels were called using Strelka 2.0.15 (65). Germline heterozygous positions were determined using GATK 3.2-2 (RRID:SCR_001876; ref. 66), and copy-number alterations were called from read counts at these positions using ASCAT v2.3. Clonality of single-nucleotide variants was assessed using Ccube (https://github.com/keyuan/ccube). Mutational signatures were extracted using SigProfilerExtractor v 1.1.7.

### Public Data

Raw data for the gastric IM samples (61) were downloaded from Gene Expression Omnibus (GEO; RRID:SCR_005012, accession number GSE134520). Raw data for normal intestinal samples (37) were downloaded from GEO (RRID:SCR_005012, accession number GSE125970). Raw data for normal gastric samples (62) were downloaded from https://dna-discovery.stanford.edu/research/datasets/.

### Read Alignment and Counting of 10x Genomics scRNA-seq Data

The Cell Ranger v2.1.1 mkref function, using default settings, takes the full *Homo sapiens* genome sequence (GRCh38) together with the *Homo sapiens* gene annotation (GRCh38.92) as input to generate a reference for read mapping. To obtain gene-specific transcript counts per sample, the Cell Ranger v3.0.1 count function with default settings was used to align and count unique molecular identifiers (UMI) per sample. This analysis was performed for all samples except for those from Sathe and colleagues (62) for which we used counts available with this dataset.

### Cell- and Sample-Level Quality Control

All samples were first inspected for diverse quality measures. For this, we plotted the total number of UMIs per cell, the total number of genes detected per cell, and the percentage of UMIs originating from mitochondrial genes per cell. Based on the visual inspection of the distribution of these quality measures, we determined a suitable lower- and upper-bound threshold per quality measure and sample (see Supplementary Table S1). The lower-bound threshold on the percentage of mitochondrial UMIs was 0.5%, whereas the upper-bound threshold was set to 25% (see Supplementary Table S1). A high percentage of mitochondrial UMIs was observed in metabolic active tissues such as NGC and ND. For the consistency of our analysis, these cells were excluded. After cell-level quality filtering, the remaining cells per sample ranged between 121 and 8,587 (see Supplementary Table S1). For downstream analysis (such as batch-correction or DE analysis), we removed genes that were not detected in any cell across all cells or the cells selected for a specific analysis. After this quality control, data from individual samples were combined and processed as a single-cell data object.

### Normalization and Batch Correction of scRNA-seq Data

After quality control, the transcriptomes of cells processed within the same library were normalized using the scran and batchelor packages (67). First, we performed scaling normalization within each batch (sample) to provide comparable results to the lowest-coverage batch using multiBatchNorm function from the batchelor package. This function returns scaled, log_2_-normalized expression values for each gene. Next, we identified top 2,000 highly variable genes using modelGeneVar and getTopHGVs functions from the scran package. Finally, to remove sample-specific effects (also referred to as batch effects) and obtain matched cell types across tissues and patients, we used the fastMNN function (with default settings) implemented in the batchelor package. In line with our previous study, we observed that cell profiles of immune and stromal components across tissue types and individual batches demonstrated relatively limited batch effects and reasoned that they could be used as “anchor” cell populations during batch correction. By applying the approach outlined above, we used the set of 2,000 genes with the highest biological variability across all samples as input genes for fastMNN. Samples were ordered based on the count of cells within individual tissue types. The tissues were corrected in the following order: “NE_Fitz,” “NSCJ_Fitz,” “NGC_Fitz,” “NGB_Ji,” “ND_Fitz,” “SMG_Fitz,” “BSCJ_Fitz,” “E-GM_Fitz,” “BE-IM_Fitz,” “CIM_Fitz,” “GIM_Fitz,” “NAG_Li,” “CAG_Li,” “GIM_Li,” “Ileum_Wang,” “Colon_Wang,” and “Rectum_Wang.” The order in which tissue types were entered had minimal effect on the batch corrections.

### Dimensionality Reduction

The batch-corrected output of the fastMNN function was used for dimensionality reduction using umap function from the umap package. In order to assess the effects of parameter selection, the analysis was performed with variable min_dist (values 0.01–0.5) and n_neighbors set to 15.

### Clustering of Batch-Corrected Single-Cell Transcriptomes

Clustering was performed on the batch-corrected output of the fastMNN function using a graph-based approach. First, the buildSNNGraph function (with default settings and setting a dataset-specific number of shared nearest-neighbors k) of the scran package was used to build a shared nearest-neighbor graph in which each node represents a cell. Next, the cluster_louvain function of the igraph package performs multilevel modularity optimization to find community structure in the graph.

### DE Testing and Marker Gene Extraction

DE testing was used to (i) identify DE genes between two conditions (e.g., cell types or tissues) or (ii) identify cell type–specific genes within one tissue. For both strategies, we used pseudobulk approaches, in which raw UMI counts per cell type, patient, and condition are summed. The edgeR R package (68) was then used to perform DE testing while incorporating variation across the different patients.

For a single pairwise comparison between two conditions, we first calculated normalization factors using the calcNormFactors function in edgeR. Next, we estimated dispersion across all pseudobulk samples using the estimateDisp function while providing a design matrix containing factors that indicate patient and condition structure. We then fitted a robust quasi-likelihood negative binomial generalized log-linear model to the count data while providing the design matrix using the glmQLFit function. DE testing was performed between the conditions using the glmTreat function with the following parameters: coef = 2, lfc = 0.5 [testing an absolute log-fold change (logFC) in mean expression >0.5]. We recorded the logFC, unshrunken logFC, log counts per million, *P* value, false discovery rate (FDR), gene name, and the directionality of the result per pairwise comparison.

To identify cell type–specific gene within one tissue, DE for all possible pairwise comparisons was tested using the strategy explained above. To combine the *P* values across all pairwise comparisons, we used the combinePValues function implemented in scran while selecting “method = simes.” This approach does not require all pairwise tests to reject the null hypothesis. The combined *P* values were corrected for multiple testing by calculating the FDR. We report the logFC and *P* value per pairwise comparison, the combined *P* value and FDR.

### Cell-Type Identification

Due to the large number of tissue- and cell-type combinations, we decided to perform a top step cell-type identification. First, we performed clustering across all batch-corrected cell and tissue types. We observed that cells fall into three broad categories: squamous epithelium (*KRT13*), columnar epithelium (*KRT8*), and nonepithelial cell types. For each cell category, we then assigned phenotypes using our previously published cell types (4). Previously, we used the “BE-Columnar” label for the BE-IM columnar cells. In the current analysis, we instead transferred the cell label of normal tissue types (either gastric or intestinal) to the BE-IM, E-GM, CIM, GIM, NAG, and CAG samples. After coarse cell-type assignments, we performed additional cell-type identification within individual cell populations as described below. This analysis relied on previously published cell-type annotation and comparison of markers with known cell types from GTEx (69) and ProteinAtlas (70).

### Epithelial Cell Types

Squamous epithelium formed a continuum of cell types, with basal cells marked by expression of *KRT5* and superficial cells marked by *KRT4*. Within this continuum, we also identified suprabasal cells (*FABP5*), and we labeled all remaining cells between basal and stratified superficial cells as intermediate squamous epithelium. These cell types were found in the NE samples and also in the squamous compartment of NSCJ, B-SCJ, and SMG samples. Detailed analysis of *KRT7* transitional cells in the NSCJ and SMG cell types was performed previously and was not undertaken in this study (4).

Columnar epithelium of NGB and NGC was annotated as chief cells (*PGA3*, *PGA5*), parietal cells (*GIF*), mucous neck cells (*MUC6*), foveolar cells (*MUC5AC*), and two types of enteroendocrine cells marked by *CHGA* and *GHRL*. Additionally, we observed *GAST*-secreting enteroendocrine cell NAG and CAG samples. Unlike NGB and NGC samples, these samples were collected in the distal stomach where *GAST*-secreting enteroendocrine cells reside.

For the intestinal cell types, we used *OLFM4* and *LGR5* as markers of stem cells, *MUC2* and *TFF3* as makers of goblet cells, and *FABP1* and *FABP2* as markers of enterocytes.

### B-cell Lineages

B cells were identified by expression of *MS4A1* (encodes CD20) and *CD19*. We further identified naive subtype of B cells and a second class of naive cells expressing Fc fragment of IgM receptor, *FCMR*. We identified multiple class switch plasma cells. We assigned major classes using genes encoding constant regions of immunoglobin heavy and light chains. We observed IgA1 (lambda) (expressing *IGHA1* and *IGLC2*), IgA1 (kappa) (expressing *IGHA1* and *IGKV1-5*), IgA2 (kappa) (expressing *IGHA2* and *IGKV4-1*), IgG (kappa) (expressing *IGHG1* and *IGKV4–1*), and IgG (lambda) (expressing *IGHG1* and *IGLC2*).

### T Cells

T-cell lineage was identified by the expression of *CD3D*. CD4 T cells expressed *CD4* and CD8 T cells expressed *CD8A*. Activated cells were marked by *CD69*. The Gamma-Delta T (gdT) cell subset expressed gamma and delta variable chain genes. T_h_1 and T_h_17 were marked by expression of *CAMK4* and *IL7R* and *RORA* with Treg expression *FOXP3*. T_mem_ cells expressed *CXCR4*. We further identified two subtypes of NK cells expressing *GZMA* in the intestine and *GZMB* in the stomach.

### Myeloid Cell Types

Macrophages were identified by the expression of *CD74*, monocytes by *FCN1* and *S100A6*, and mast cells by the expression of *CPA3*. Among dendritic cells (DC), we identified plasmacytoid DC (pDC; *CLEC4C*, *JCHAIN*) and cDC1 (*CLEC9A*). We used the expression of *CD69* to mark activated cell types.

### Stromal and Endothelial Cell Types

Endothelial cells expressing *PECAM1* and *CDH5* were subdivided into arterial (*GJA4*, *HEY1*, *HEY2*, and *EFNB2*) and venous (*ACKR1* and *VWF*) subtypes. As described in Results, we used the previous annotation of fibroblast by Davidson and colleagues (38), and they were separated into S1 (*CFD, PDPN*), S2 (*CXCL14, POSTN*), and S3 (*ACTA2*) subtypes.

### Disease State Cell-Type Annotation and EGIC Score Calculation

First, cell types in the esophageal and gastric IM samples were assigned using the *louvain* clustering approach. In this approach, we transferred labels from normal cell types to other cells that were presented in the same cluster. Second, we used SingleR (20) to perform unbiased annotation of cell types using annotated normal tissues as reference transcriptome. SingleR training was performed using the trainSingleR function using the Wilcox ranked sum test, and 20 marker genes were retained per cell type. We saw high concordance between manual cell annotation using *louvain* clustering and SingleR for nonepithelial cell types. However, there was strong discordance between labels of the epithelial cells. As described in Results, we noticed that individual cells have properties of two tissue types, and each method could not accurately transfer labels from normal to IM cells.

EGIC score was calculated using MuSiC (21). MuSiC was designed for deconvolution of bulk RNA-seq samples using a two-step process. First, well-annotated cell types from multiple subjects are used to identify cell type–specific genes with low cross-subject variance. These genes are then used for deconvolution of individual bulk samples. During EGIC score calculation, we assumed that individual cells can be treated as bulk samples (the dominant marker genes are expressed in the majority of cells), and the contribution of normal cell types can be calculated for each cell. We used normal cells as a validation cohort, and using this approach, we were able to reconstruct cell identity. The strength of this approach lies in the fact that raw gene expression counts are used to assign labels to cell types and the relative contribution of different cell types can be calculated.

### Trajectory Analysis

First, we used TSCAN to reconstruct minimal spanning trees across the epithelial cells using the distances between mutual nearest-neighbor pairs between clusters (71). We ran quickPseudotime function on the principal component analysis (PCA) data with outgroup and with.mnn options set to TRUE.

We also used monocle3 to construct cell-to-cell pseudotime across selected cell types (24, 25). We first performed dimensionality reduction using the preprocess_cds function, followed by batch correction using *align_cds*. Finally, we constructed the principal graph across the cells using reversed graph embedding from the function learn_graph. The graphs were anchored to the specific cell population using order_cells.

### Immunofluorescence and HALO Qualification

Formalin-fixed, paraffin-embedded slides of disease-free organ donor GEJ tissues and patient biopsies were stained for immunofluorescence to identify S1/S2/S3 using routine immunofluorescence protocols. Briefly, slides were dewaxed and rehydrated with xylene, 100%, 95%, and 70% ethanol. Antigen retrieval was performed by boiling the slides in Tris-EDTA pH 9 buffer for 4 minutes before staining for CD31 (ab9498, Abcam), CD34 (AF7227, all R&D Systems unless otherwise stated), periostin (AF3548), or αSMA (MAB1420) overnight at 4°C. Samples were then washed and incubated with appropriate Alexa Fluor–conjugated secondary antibodies before counterstaining nuclei with DAPI. For each sample, two adjacent slides were stained for S1 quantification and S2/S3 quantification, respectively (Supplementary Table S1).

Slides were scanned using Zeiss Axio Scanner with a resolution of 0.163 μm per pixel. Images were quantified using HALO (Indica Lab). S1, S2, and S3 were quantified as staining areas (μm^2^) of CD34^+^/CD31^−^, POSTN^+^/αSMA^−^, and αSMA^+^, respectively. The abundance of each within the total fibroblast compartment was then calculated.

NE, NGC, and NGB were cross-sections of GEJ tissues from disease-free organ donors. The tissues were obtained as longitude tubes that included both NE and gastric compartments with a total length of approximately 5 cm. Notably, NGC was defined as a 3-mm-long gastric compartment immediately after the Z-line. NGB was defined as areas that were 3 to 4 cm away from the Z-line. Only epithelium and lamina propria were quantified, and the area of muscularis mucosa was excluded to avoid false-positive staining for αSMA^+^ S3 fibroblasts. E-GM, BE-IM, and GIM were quantified from patient endoscopic biopsies.

### Data Availability

The data generated in this study are available within the article and its supplementary data files. The sequencing reads used in this study are available from the European Genome-phenome Archive (accession: EGAD00001010074). Publicly available expression profile data analyzed in this study were obtained from GEO (RRID:SCR_005012) at GSE134520, GSE125970, and GSE183904 or were downloaded from https://dna-discovery.stanford.edu/research/datasets/. Raw and processed data can be downloaded from the cellxgene single-cell RNA-seq atlas (https://cellxgene.cziscience.com/collections/a18474f4-ff1e-4864-af69-270b956cee5b). This website also contains interactive visualization and analysis tools. The code used for processing raw data and downstream analysis and the steps used during figure generation can be accessed at https://github.com/karolno/BE-GIM_Comparison.

## Supplementary Material

Supplementary Table S1 This file contains details of individual samples (including clinical data) used in the sutdy

Supplementary Table S2 This table contains marker genes identified in each cluster of cells.

Supplementary Table S3 This table contains marker genes identified in the pairwise comparison of clusters defined as columnar cells.

Supplementary Table S4 This table contains results of pairwise differential expression analysis between E-GM and gastric tissues and Atrophic gastritis and gastric tissue.

Supplementary Table S5 This table contains details of mutations identified in WGS of E-GM samples.

Supplementary Table S6 This table contains marker genes identified in the pairwise comparison of clusters identified in fibroblast population

Supplementary Figures This file captions for supplementary tables and supplementary figures S1-S28 together with their legends
