# Supplementary material for: Single-cell RNA sequencing unifies developmental programs of Esophageal and Gastric Intestinal Metaplasia
Source: Cancer Discov. Author manuscript; Available in PMC 2023 Jun 23. (PMC10236154; doi:10.1158/2159-8290.CD-22-0824)
Supplement: Supplementary legends [file EMS172799-supplement-Supplementary_legends.pdf]

Table Legends:

Table S1: Summary of clinical and sequencing data for all samples analyzed

Table S2: Marker genes identified for all clusters identified in all samples

Table S3: Marker genes identified for the columnar cells

Table S4: Results of differential gene analysis between E-GM and normal tissues and NAG/CAG and normal tissues

Table S5: The summary of mutations detected in E-GM and IM samples

Table S6: Marker genes identified for the fibroblasts

Supplementary Figures and Legends

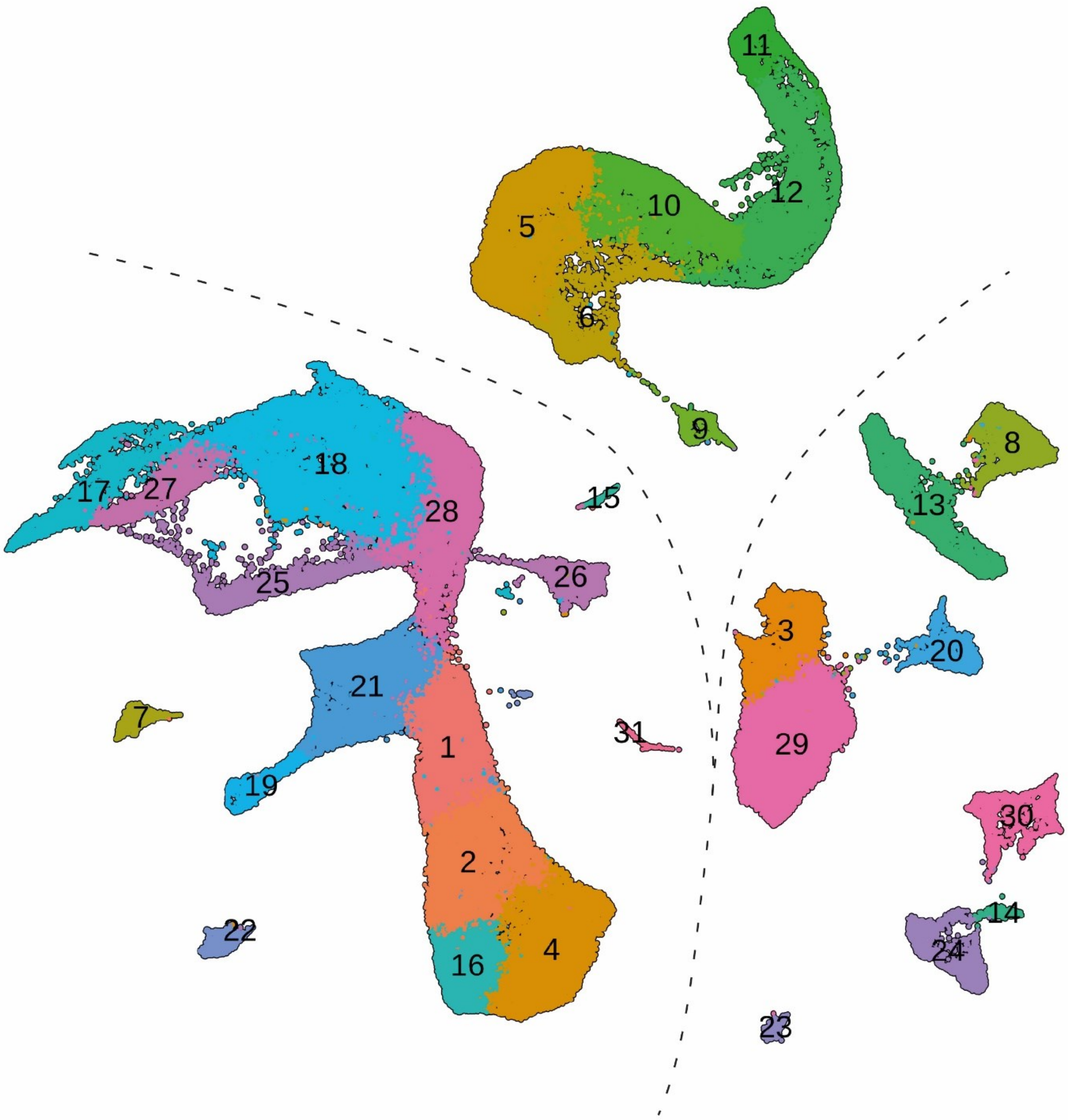

11
12
10
5
6
9
8
13
18
17
27
28
15
25
26
3
20
21
7
31
1
29
19
30
2
14
22
4
24
16
23

Figure S1: Clustering of all cell types.

UMAP of batch corrected cells analyzed in this study with computationally identified cell clusters highlighted.

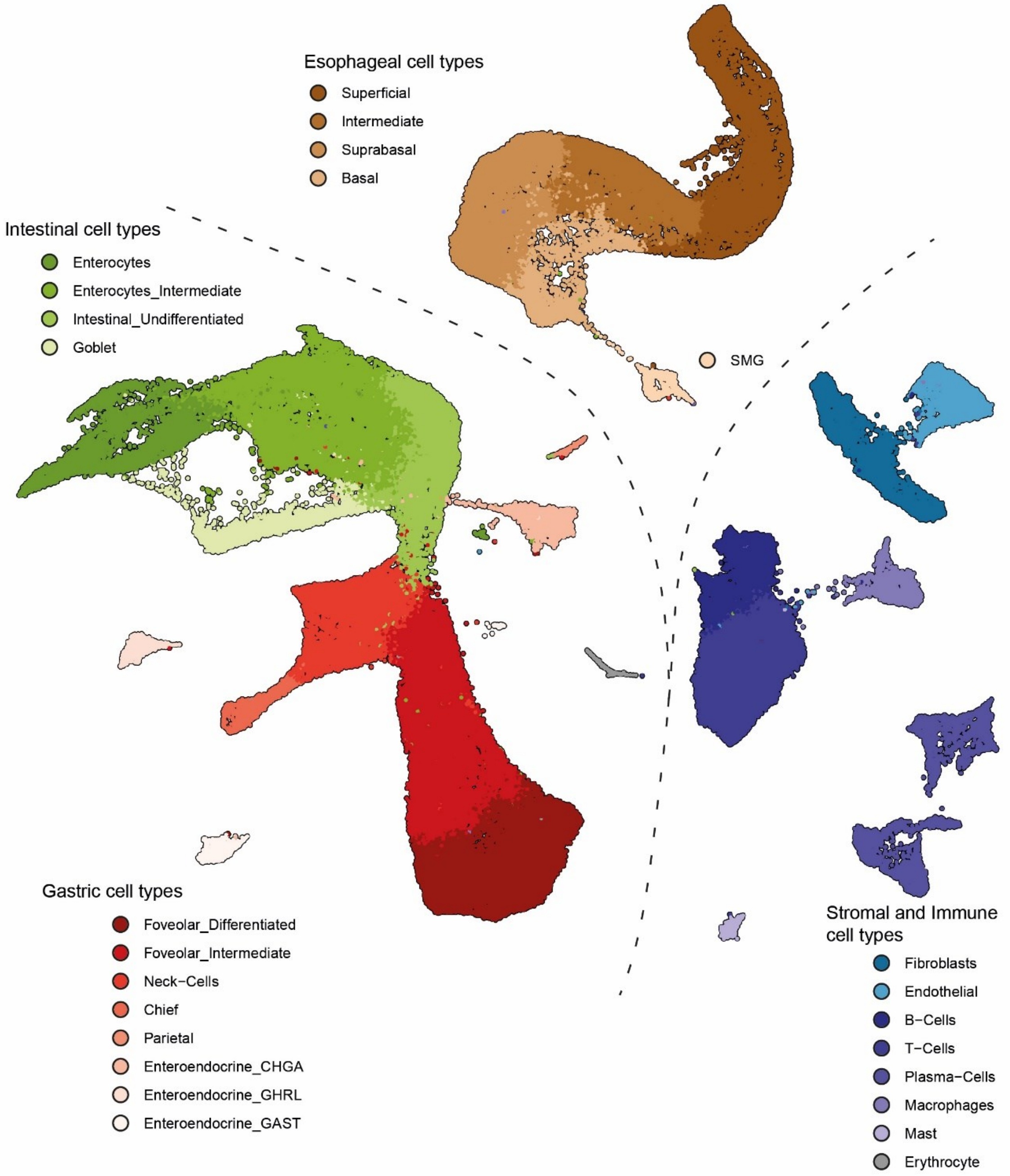

Esophageal cell types
Superficial
Intermediate
Suprabasal
Basal
SMG
Intestinal cell types
Enterocytes
Enterocytes_Intermediate
Intestinal_Undifferentiated
Goblet
Gastric cell types
Foveolar_Differentiated
Foveolar_Intermediate
Neck−Cells
Chief
Parietal
Enteroendocrine_CHGA
Enteroendocrine_GHRL
Enteroendocrine_GAST
Stromal and Immune cell types
Fibroblasts
Endothelial
B−Cells
T−Cells
Plasma−Cells
Macrophages
Mast
Erythrocyte

Figure S2: Annotation of all cell types.

UMAP of batch corrected cells analyzed in this study with manually annotated cell types highlighted.

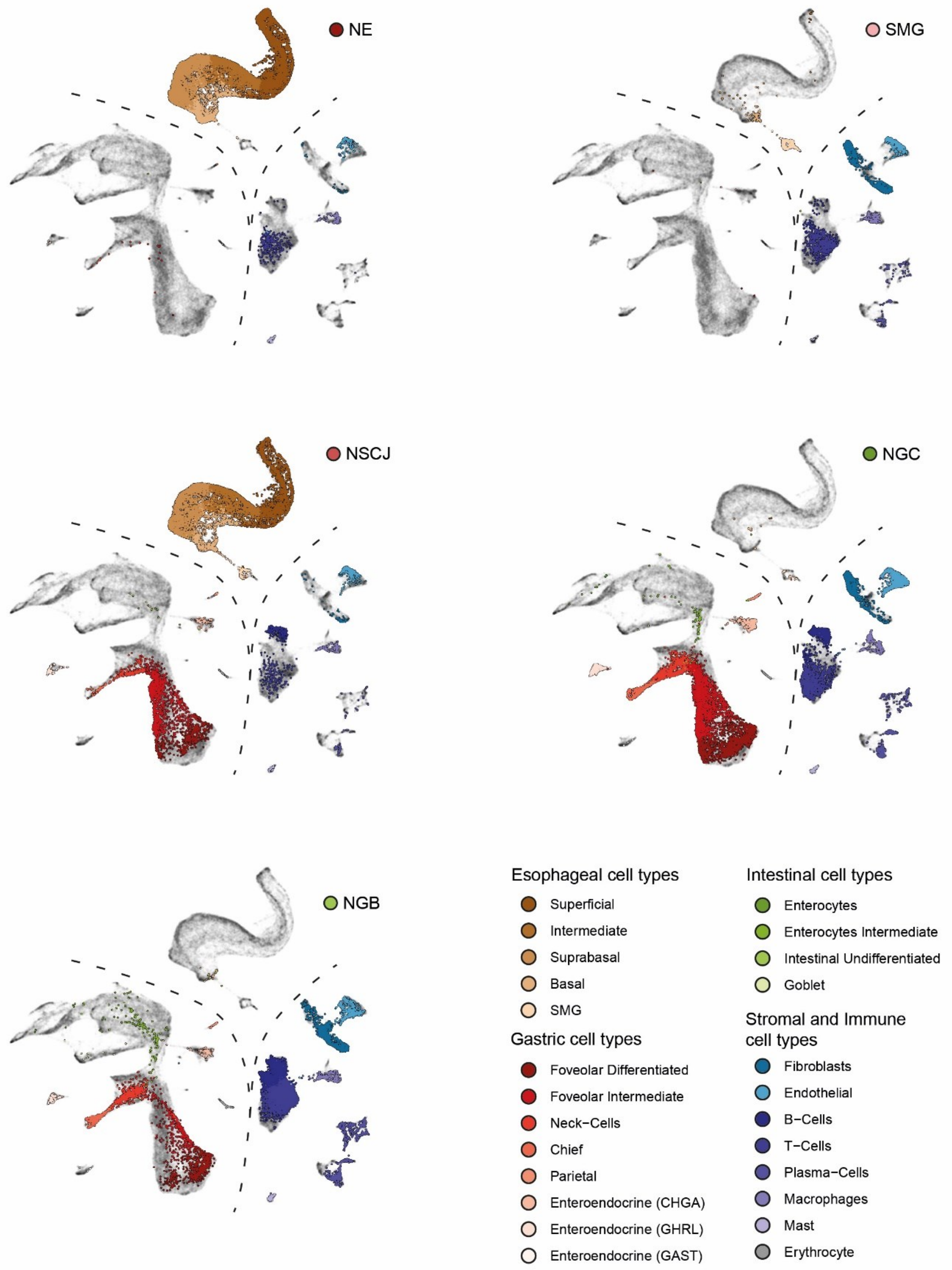
NE
SMG
NSCJ
NGC
NGB
Esophageal cell types
Superficial
Intermediate
Suprabasal
Basal
SMG
Gastric cell types
Foveolar Differentiated
Foveolar Intermediate
Neck−Cells
Chief
Parietal
Enteroendocrine (CHGA)
Enteroendocrine (GHRL)
Enteroendocrine (GAST)
Intestinal cell types
Enterocytes
Enterocytes Intermediate
Intestinal Undifferentiated
Goblet
Stromal and Immune cell types
Fibroblasts
Endothelial
B−Cells
T−Cells
Plasma−Cells
Macrophages
Mast
Erythrocyte

Figure S3: Annotation of all cell types in normal tissue types of stomach and esophagus

UMAP of batch corrected cells analyzed in this study with manually annotated cell types highlighted in the NE, SMG, NSCJ, NGC and NGB samples. Please note that the NGB samples were collected from tissues adjacent to gastric cancer samples (Sathe et al.) and the cells from at least patient seem to contain intestinal foveolar-like and goblet cells suggesting presence of gastric IM in this patient.

Abbreviation: NE – normal squamous esophagus, SMG – esophageal submucosal glands, NSCJ – normal squamo-columnar junction, NGC – normal gastric cardia, NGB – normal gastric body.

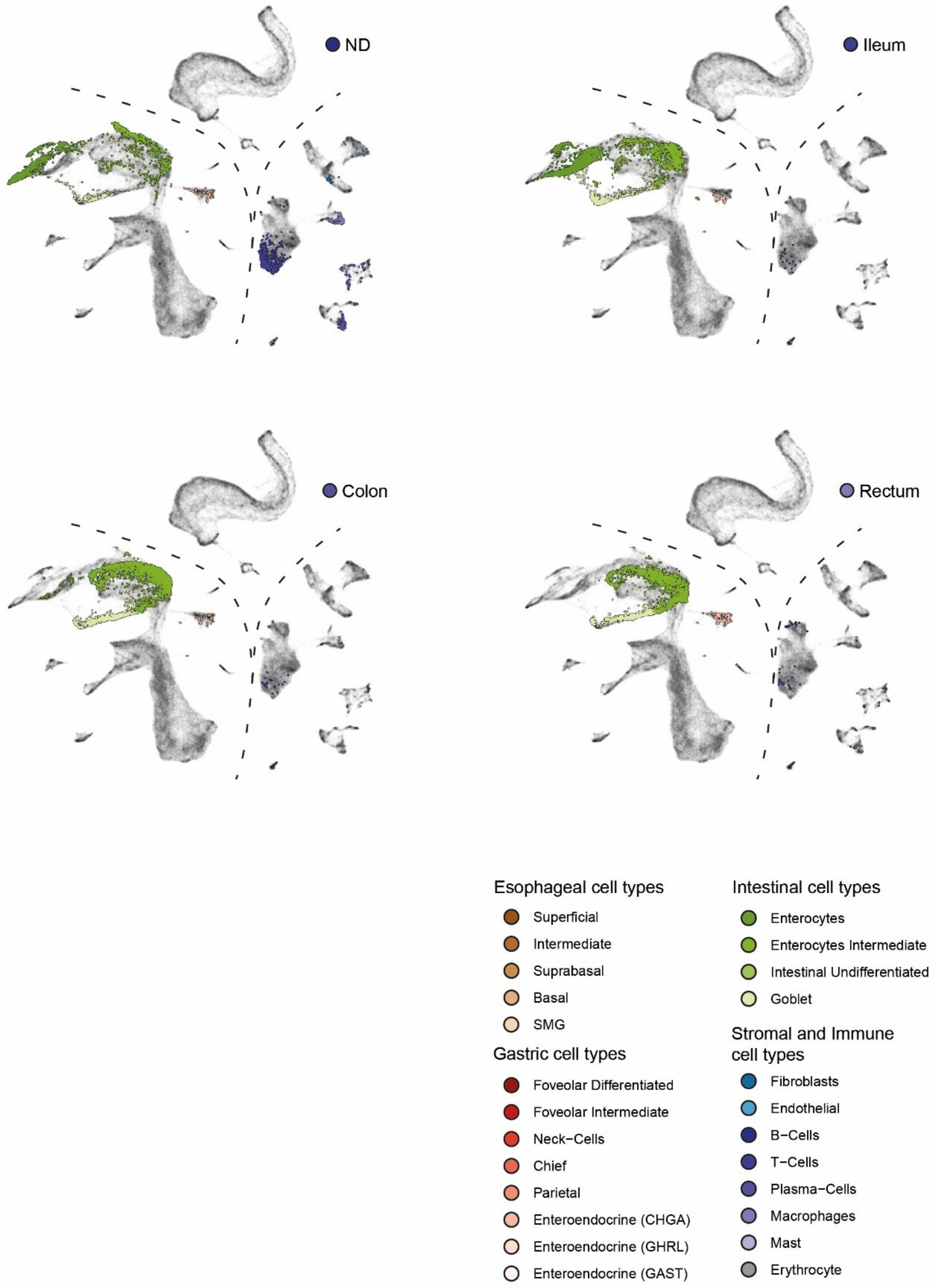

ND
Ileum
Colon
Rectum
Esophageal cell types
Superficial
Intermediate
Suprabasal
Basal
SMG
Gastric cell types
Foveolar Differentiated
Foveolar Intermediate
Neck−Cells
Chief
Parietal
Enteroendocrine (CHGA)
Enteroendocrine (GHRL)
Enteroendocrine (GAST)
Intestinal cell types
Enterocytes
Enterocytes Intermediate
Intestinal Undifferentiated
Goblet
Stromal and Immune cell types
Fibroblasts
Endothelial
B−Cells
T−Cells
Plasma−Cells
Macrophages
Mast
Erythrocyte

Figure S4: Annotation of all cell types in normal tissue types of intestine

UMAP of batch corrected cells analyzed in this study with manually annotated cell types highlighted in the ND, Ileum, Colon and Rectum samples. Please note that Ileum, Colon and Rectum were actively enriched for the epithelial cells by the authors and very few non-epithelial cells were identified.

Abbreviation: ND – normal duodenum.

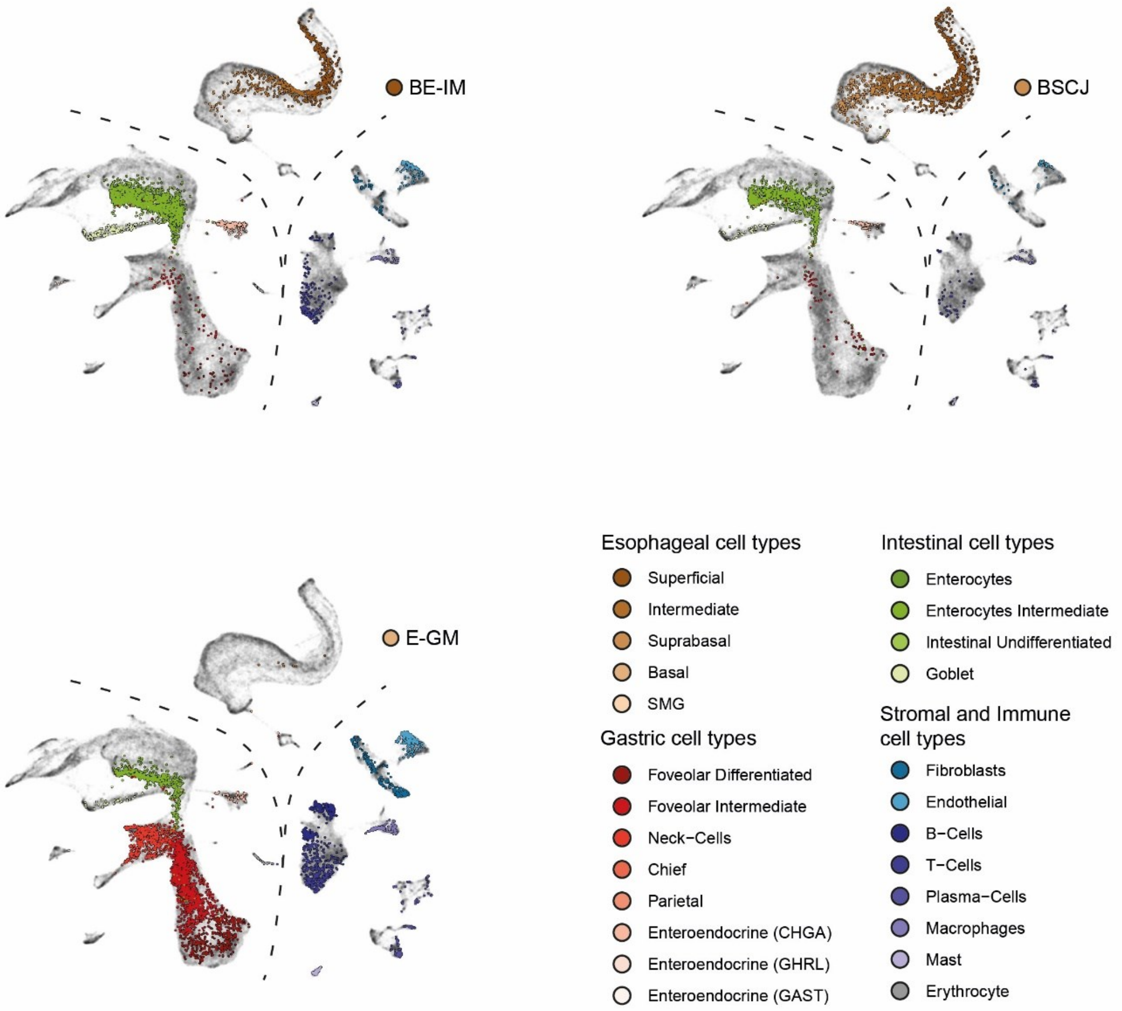
BE-IM
BSCJ
E-GM
Esophageal cell types
Superficial
Intermediate
Suprabasal
Basal
SMG
Gastric cell types
Foveolar Differentiated
Foveolar Intermediate
Neck−Cells
Chief
Parietal
Enteroendocrine (CHGA)
Enteroendocrine (GHRL)
Enteroendocrine (GAST)
Intestinal cell types
Enterocytes
Enterocytes Intermediate
Intestinal Undifferentiated
Goblet
Stromal and Immune cell types
Fibroblasts
Endothelial
B−Cells
T−Cells
Plasma−Cells
Macrophages
Mast
Erythrocyte

Figure S5: Annotation of all cell types in esophageal IM tissue types

UMAP of batch corrected cells analyzed in this study with manually annotated cell types highlighted in the BE, BSCJ and GM samples. Please note that one BE sample contained squamous cells that originate from squamous island confirmed on the adjacent H&E.

Abbreviation: BE-IM – Barrett's esophagus, E-GM – gastric metaplasia of esophagus, BSCJ – squamo-columnar junction between NE and BE.

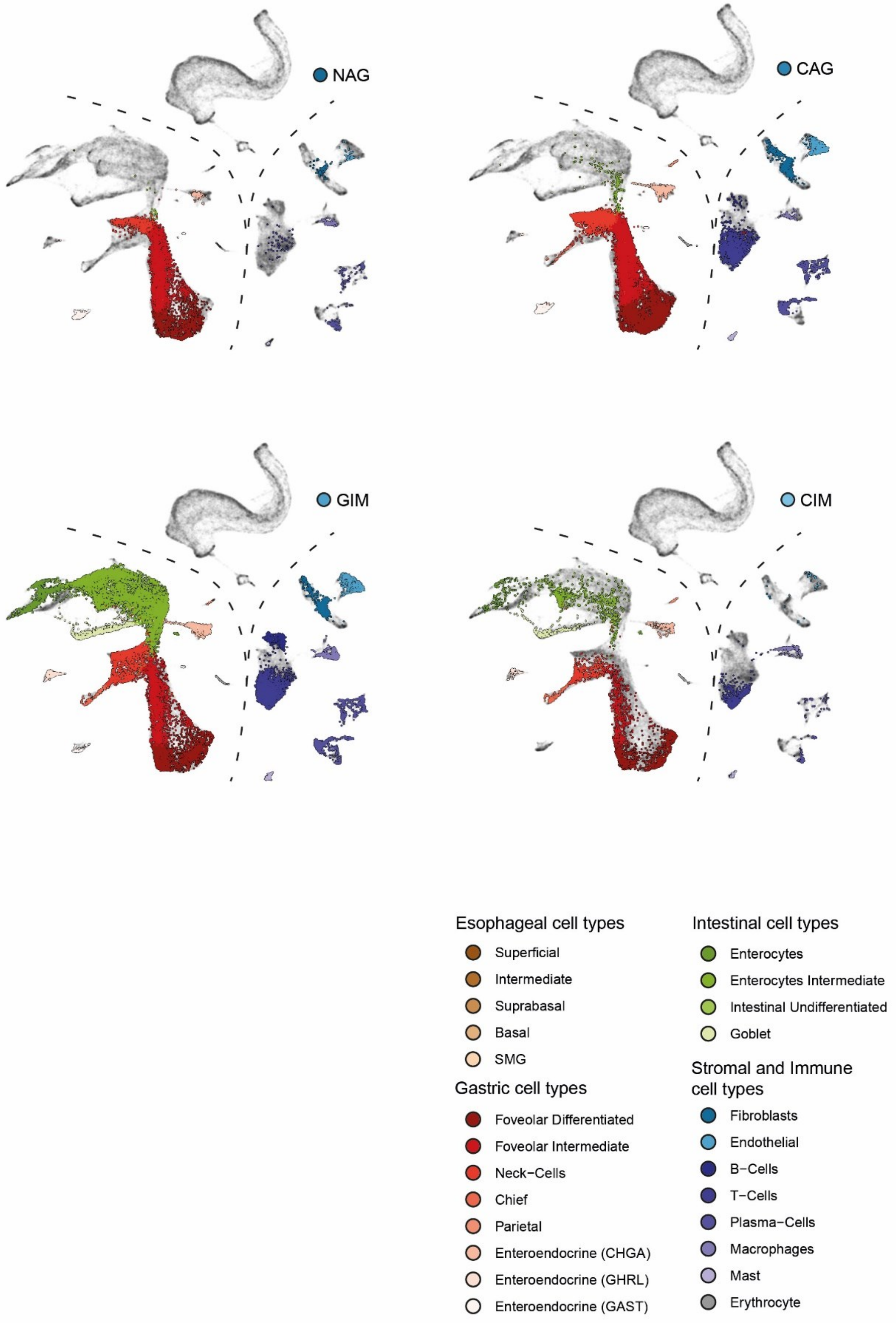
NAG
CAG
GIM
CIM
Esophageal cell types
Superficial
Intermediate
Suprabasal
Basal
SMG
Gastric cell types
Foveolar Differentiated
Foveolar Intermediate
Neck−Cells
Chief
Parietal
Enteroendocrine (CHGA)
Enteroendocrine (GHRL)
Enteroendocrine (GAST)
Intestinal cell types
Enterocytes
Enterocytes Intermediate
Intestinal Undifferentiated
Goblet
Stromal and Immune cell types
Fibroblasts
Endothelial
B−Cells
T−Cells
Plasma−Cells
Macrophages
Mast
Erythrocyte

Figure S6: Annotation of all cell types in stomach IM tissue types

UMAP of batch corrected cells analyzed in this study with manually annotated cell types highlighted in the NAG, CAG, GIM and CIM samples. Please note that NAG, CAG and GIM samples were collected in the pyloric stomach by Zhang et al. and these sample are the only samples containing Gastrin (GAST) producing enteroendocrine cells.

Abbreviation: CIM – cardia intestinal metaplasia, GIM – gastric intestinal metaplasia, NAG – non-chronic atrophic gastritis, CAG – chronic atrophic gastritis.

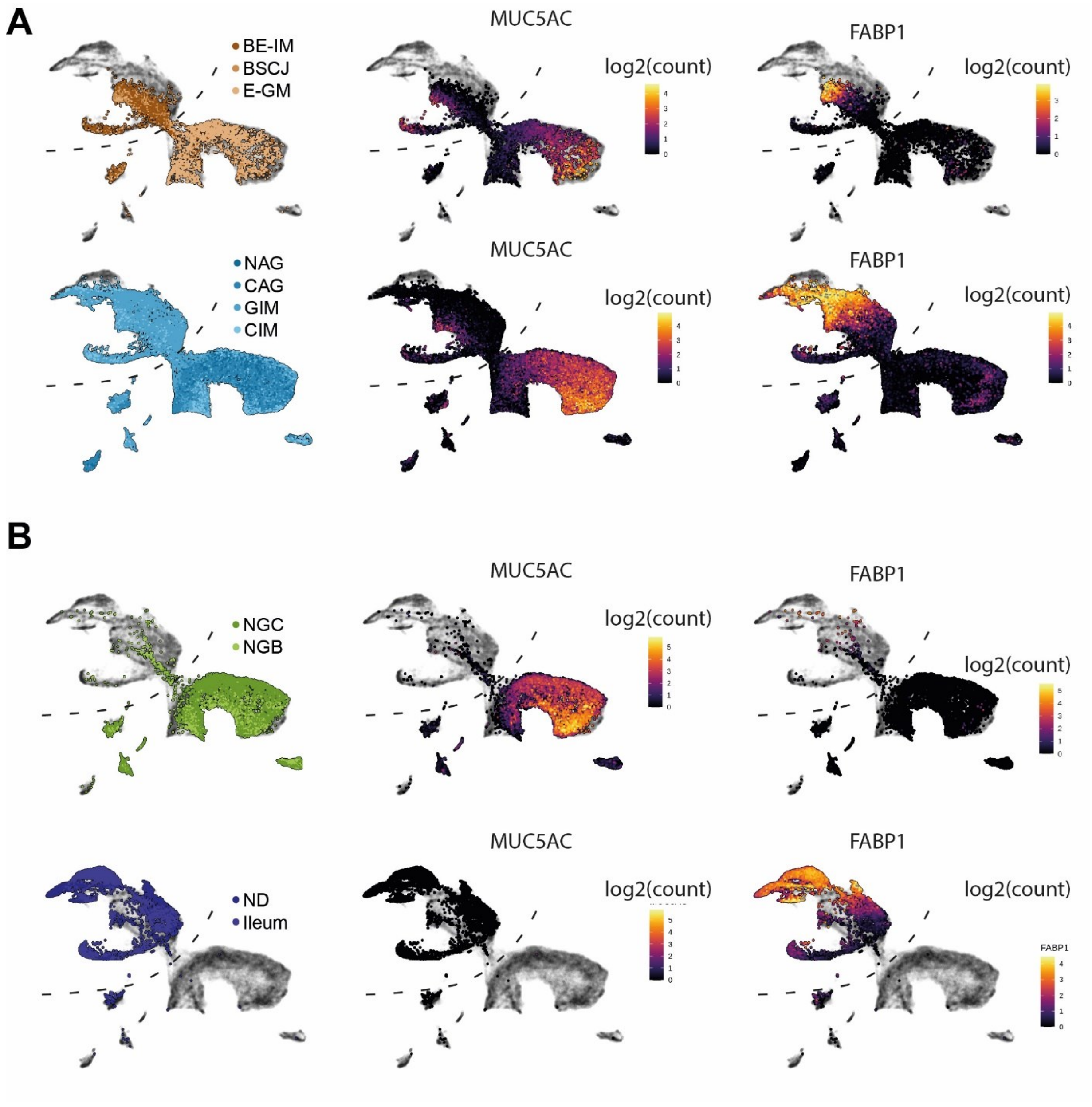

A
BE-IM
BSCJ
E-GM
MUC5AC
log2(count)
FABP1
log2(count)
NAG
CAG
GIM
CIM
MUC5AC
log2(count)
FABP1
log2(count)
B
NGC
NGB
MUC5AC
log2(count)
FABP1
log2(count)
ND
Ileum
MUC5AC
log2(count)
FABP1
log2(count)
FABP1

Figure S7: Expression of MUC5AC and FABP1 markers in normal gastric and intestinal cell types.

A) UMAP of esophageal IM (E-GM, BE-IM, BSCJ) and stomach IM (NAG, CAG, GIM, CIM) with tissue types highlighted. The middle UMAP show expression of MUC5AC (marker of gastric foveolar cells) in the corresponding tissues. The right UMAP show expression of FABP1 (marker of intestinal enterocytes) in the corresponding tissues.
B) UMAP of gastric (NGC and NBG) and small intestinal (ND, Ileum) cells with tissue types highlighted. The middle UMAP show expression of MUC5AC (marker of gastric foveolar cells) in the corresponding tissues. The right UMAP show expression of FABP1 (marker of intestinal enterocytes) in the corresponding tissues.

Abbreviation: BE-IM – Barrett's esophagus, E-GM – gastric metaplasia of esophagus, BSCJ – squamo-columnar junction between NE and BE, CIM – cardia intestinal metaplasia, GIM – gastric intestinal metaplasia, NAG – non-chronic atrophic gastritis, CAG – chronic atrophic gastritis NGC – normal gastric cardia, NGB – normal gastric body, ND – normal duodenum.

Normal duodenum (ND)

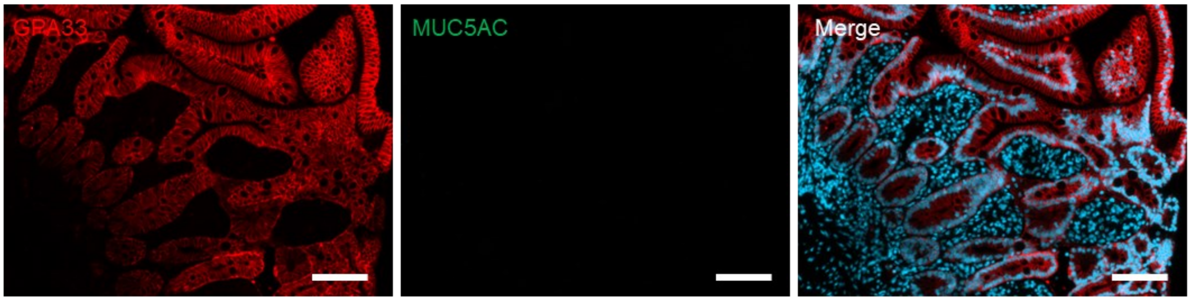

Normal gastric cardia (NGC)

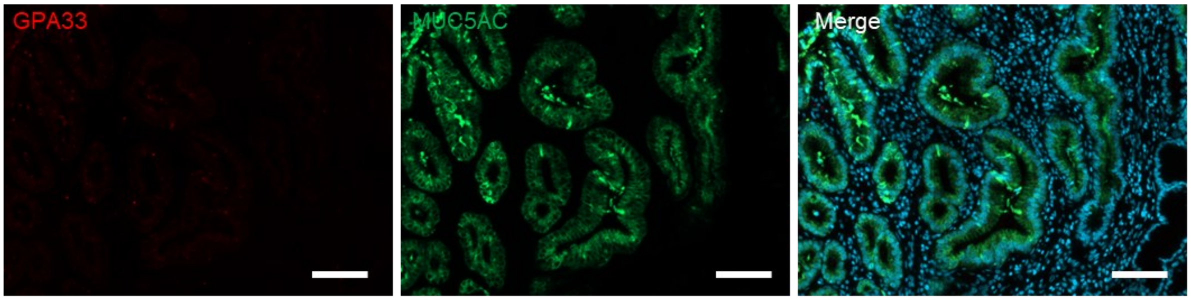

E-GM

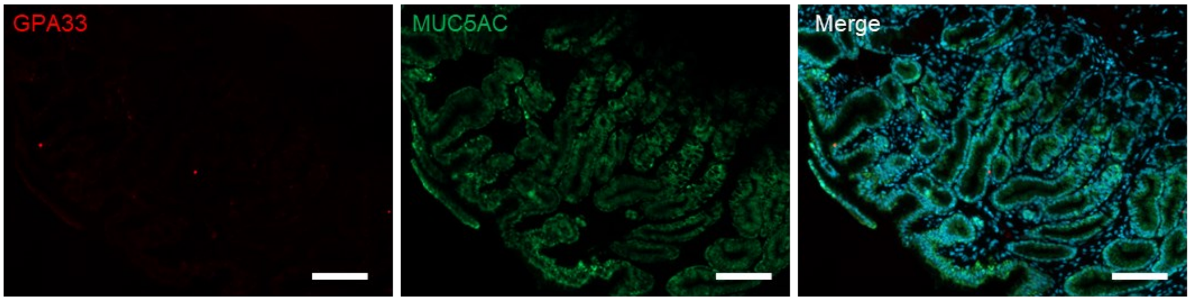

BE-IM

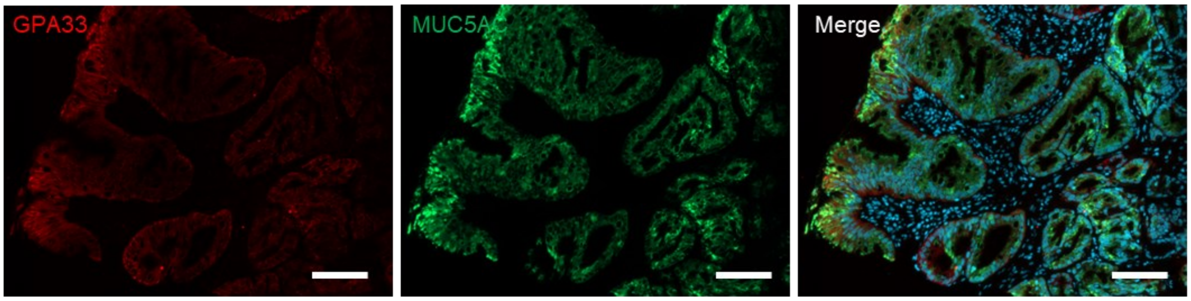

GIM

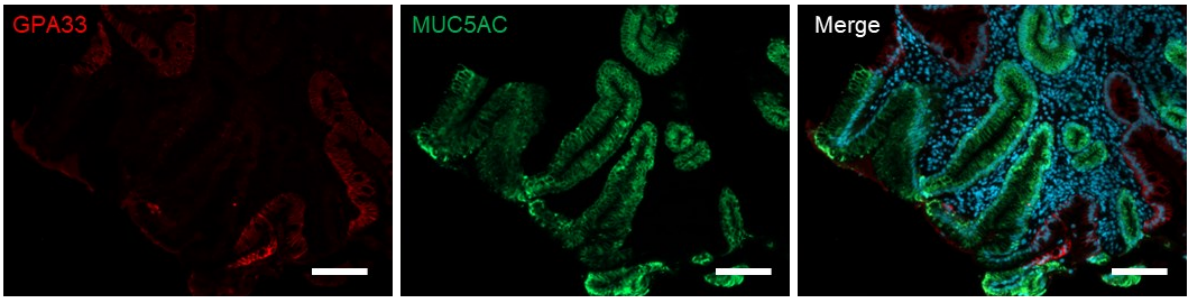

Scale bar = 100 μm

Figure S8: Co-immunofluorescent staining with MUC5AC and GPA33

Co-immunofluorescent staining of Normal gastric cardia (NGC), duodenum (ND), Esophagus with gastric metaplasia (E-GM), Barrett's esophagus with intestinal metaplasia (BE-IM) and gastric intestinal metaplasia (GIM) shows co-expression of differentiated intestinal (GPA33) and gastric (MUC5AC) markers in both types of intestinal metaplasia. scale bar: 100 µm. Related to figure 3C. Images are representative of 15 patients (NGC = 2, ND = 2, BE-IM = 4, GIM = 4, E-GM = 3). Each patient has 1 to 4 biopsies.

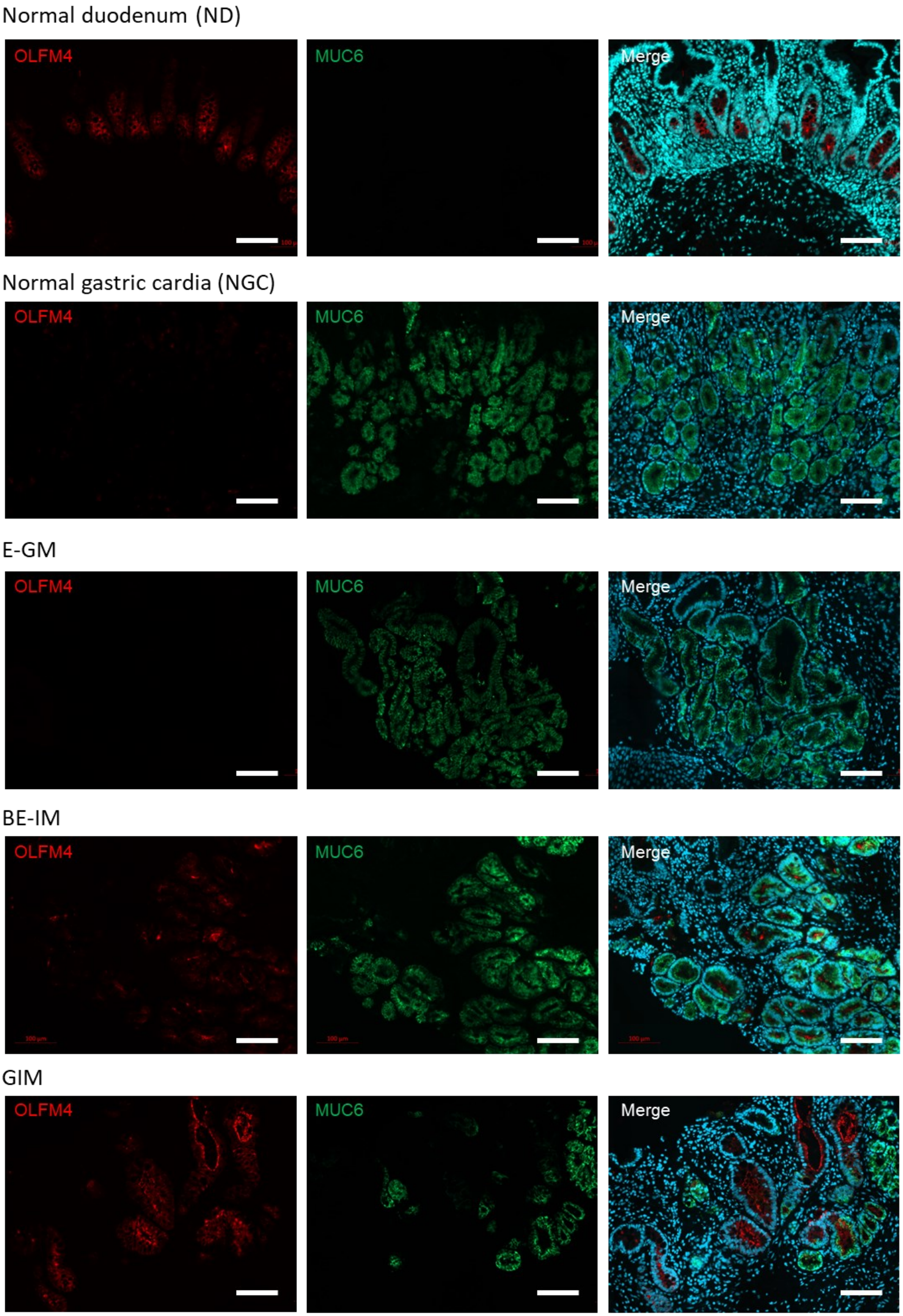

Scale bar = 100 μm

Figure S9: Co-immunofluorescent staining with MUC6 and OLFM4

Co-immunofluorescent staining of Normal gastric cardia (NGC), duodenum (ND), Esophagus with gastric metaplasia (E-GM), Barrett's esophagus with intestinal metaplasia (BE-IM) and gastric intestinal metaplasia (GIM) shows co-expression of intestinal (MUC6) and gastric (OLFM4) progenitor markers in both types of intestinal metaplasia. scale bar: 100 µm. Related to figure 3C. Images are representative of 15 patients (NGC = 2, ND = 2, BE-IM = 4, GIM = 4, E-GM = 3). Each patient has 1 to 4 biopsies.

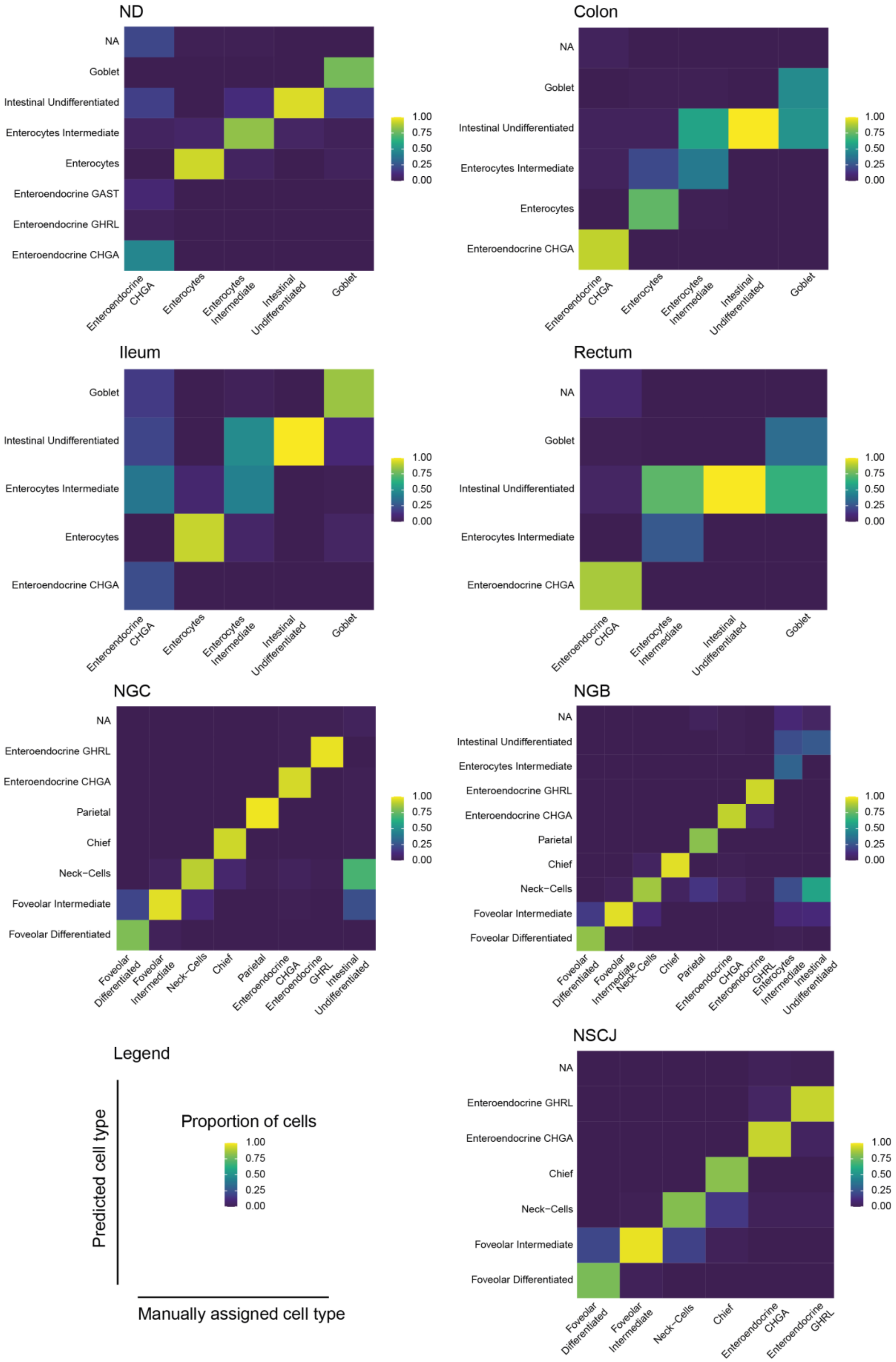

ND
NA
Goblet
Intestinal Undifferentiated
Enterocytes Intermediate
Enterocytes
Enteroendocrine GAST
Enteroendocrine GHRL
Enteroendocrine CHGA
Enteroendocrine CHGA
Enterocytes
Enterocytes Intermediate
Intestinal Undifferentiated
Goblet
Colon
Ileum
Rectum
NGC
Enteroendocrine GHRL
Parietal
Chief
Neck−Cells
Foveolar Intermediate
Foveolar Differentiated
NGB
NSCJ
1.00
0.75
0.50
0.25
0.00
Legend
Proportion of cells
Predicted cell type
Manually assigned cell type

Figure S10: Confusion matrix of cell type annotation in the normal tissue types using SingleR annotation.

Confusion matrix displaying the proportion of cells that were annotated using SingleR (y-axis) that overlap the given reference cell types (x-axis).

Abbreviation: NSCJ – normal squamo-columnar junction, NGC – normal gastric cardia, NGB – normal gastric body, ND – normal duodenum,

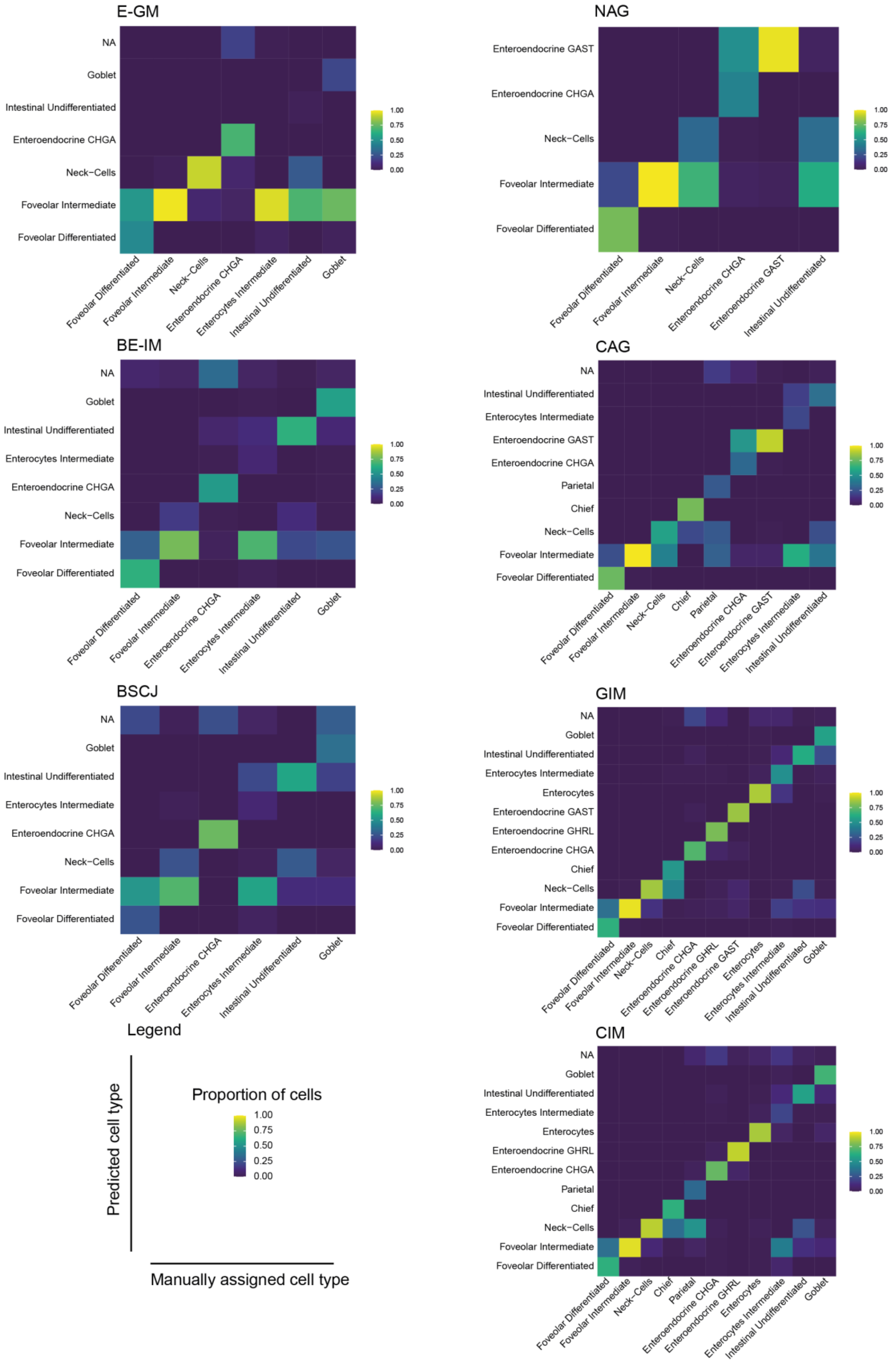
E-GM
NA
Goblet
Intestinal Undifferentiated
Enteroendocrine CHGA
Neck−Cells
Foveolar Intermediate
Foveolar Differentiated
Foveolar Differentiated
Foveolar Intermediate
Neck−Cells
Enteroendocrine CHGA
Enterocytes Intermediate
Intestinal Undifferentiated
Goblet
1.00
0.75
0.50
0.25
0.00
NAG
Enteroendocrine GAST
Enteroendocrine CHGA
Neck−Cells
Foveolar Intermediate
Foveolar Differentiated
Foveolar Differentiated
Foveolar Intermediate
Neck−Cells
Enteroendocrine CHGA
Enteroendocrine GAST
Intestinal Undifferentiated
1.00
0.75
0.50
0.25
0.00
BE-IM
NA
Goblet
Intestinal Undifferentiated
Enterocytes Intermediate
Enteroendocrine CHGA
Neck−Cells
Foveolar Intermediate
Foveolar Differentiated
Foveolar Differentiated
Foveolar Intermediate
Enteroendocrine CHGA
Enterocytes Intermediate
Intestinal Undifferentiated
Goblet
1.00
0.75
0.50
0.25
0.00
CAG
NA
Intestinal Undifferentiated
Enterocytes Intermediate
Enteroendocrine GAST
Enteroendocrine CHGA
Parietal
Chief
Neck−Cells
Foveolar Intermediate
Foveolar Differentiated
Foveolar Differentiated
Foveolar Intermediate
Neck−Cells
Chief
Parietal
Enteroendocrine CHGA
Enteroendocrine GAST
Enterocytes Intermediate
Intestinal Undifferentiated
1.00
0.75
0.50
0.25
0.00
BSCJ
NA
Goblet
Intestinal Undifferentiated
Enterocytes Intermediate
Enteroendocrine CHGA
Neck−Cells
Foveolar Intermediate
Foveolar Differentiated
Foveolar Differentiated
Foveolar Intermediate
Enteroendocrine CHGA
Enterocytes Intermediate
Intestinal Undifferentiated
Goblet
1.00
0.75
0.50
0.25
0.00
GIM
NA
Goblet
Intestinal Undifferentiated
Enterocytes Intermediate
Enterocytes
Enteroendocrine GAST
Enteroendocrine GHRL
Enteroendocrine CHGA
Chief
Neck−Cells
Foveolar Intermediate
Foveolar Differentiated
Foveolar Differentiated
Foveolar Intermediate
Neck−Cells
Chief
Enteroendocrine CHGA
Enteroendocrine GHRL
Enteroendocrine GAST
Enterocytes
Enterocytes Intermediate
Intestinal Undifferentiated
Goblet
1.00
0.75
0.50
0.25
0.00
Legend
Predicted cell type
Proportion of cells
1.00
0.75
0.50
0.25
0.00
Manually assigned cell type
CIM
NA
Goblet
Intestinal Undifferentiated
Enterocytes Intermediate
Enterocytes
Enteroendocrine GHRL
Enteroendocrine CHGA
Parietal
Chief
Neck−Cells
Foveolar Intermediate
Foveolar Differentiated
Foveolar Differentiated
Foveolar Intermediate
Neck−Cells
Chief
Parietal
Enteroendocrine CHGA
Enteroendocrine GHRL
Enterocytes
Enterocytes Intermediate
Intestinal Undifferentiated
Goblet
1.00
0.75
0.50
0.25
0.00

Figure S11: Confusion matrix of cell type annotation in the IM tissue types using SingleR annotation.

Confusion matrix displaying the proportion of cells that were annotated using SingleR (y-axis) that overlap the given reference cell types (manually annotated using reference genes from normal tissues, x-axis).

Abbreviation: BE-IM – Barrett's esophagus, E-GM – gastric metaplasia of esophagus, BSCJ – squamo-columnar junction between NE and BE, CIM – cardia intestinal metaplasia, GIM – gastric intestinal metaplasia, NAG – non-chronic atrophic gastritis, CAG – chronic atrophic gastritis.

s

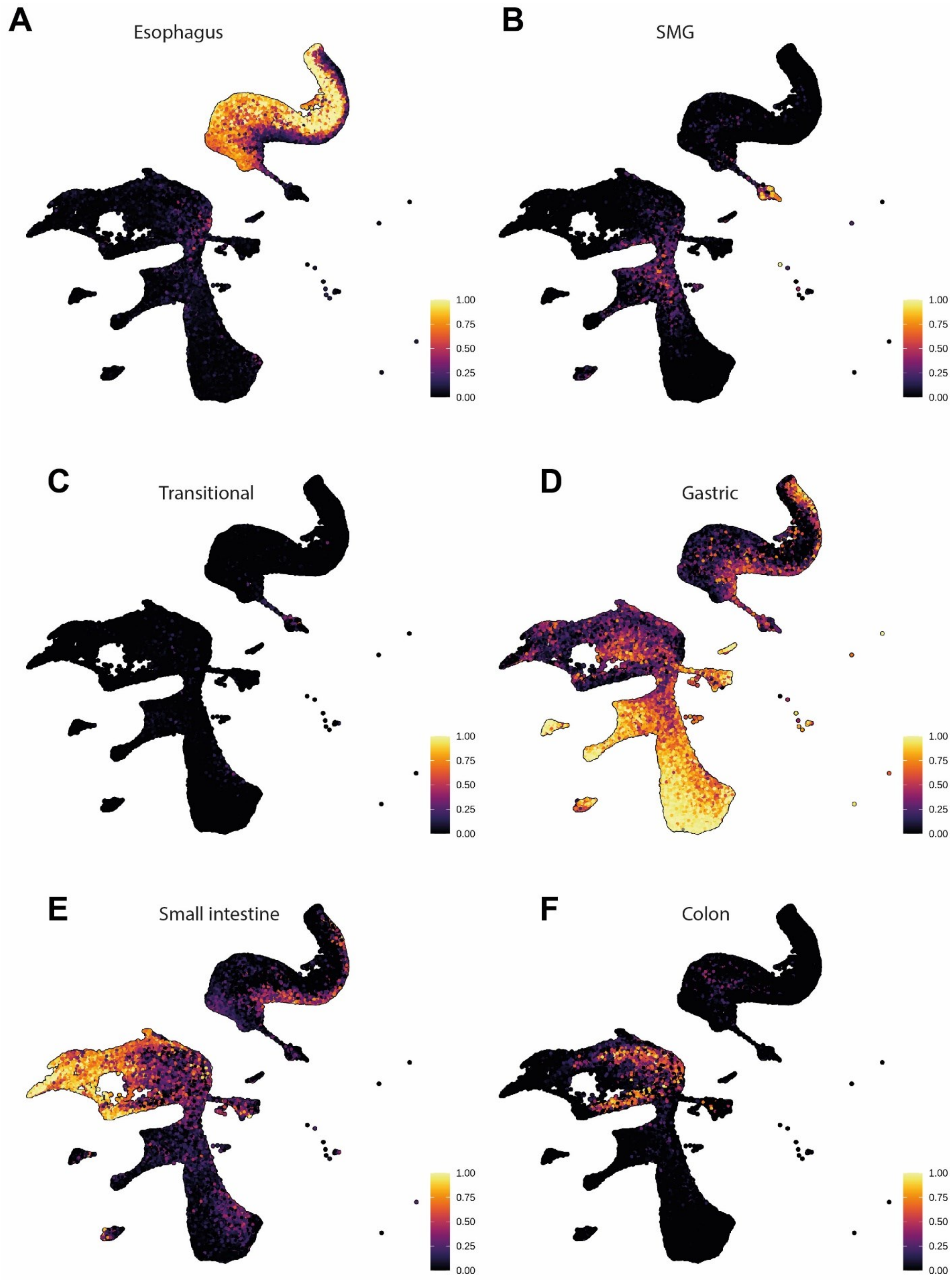
A
Esophagus
B
SMG
C
Transitional
D
Gastric
E
Small intestine
F
Colon
1.00
0.75
0.50
0.25
0.00

Figure S12: MuSiC accurately predicts contribution of cell types to know tissue types.

UMAP project of all columnar cell types (same coordinates as figure 1B) with the contribution of Esophageal (A), SMG (B), Transitional (C), Gastric (D), Small intestinal (E) and Colonic (F) phenotypes highlighted. The contribution of Transitional phenotype is minimal as these cells share most phenotypic features with SMG cell types.

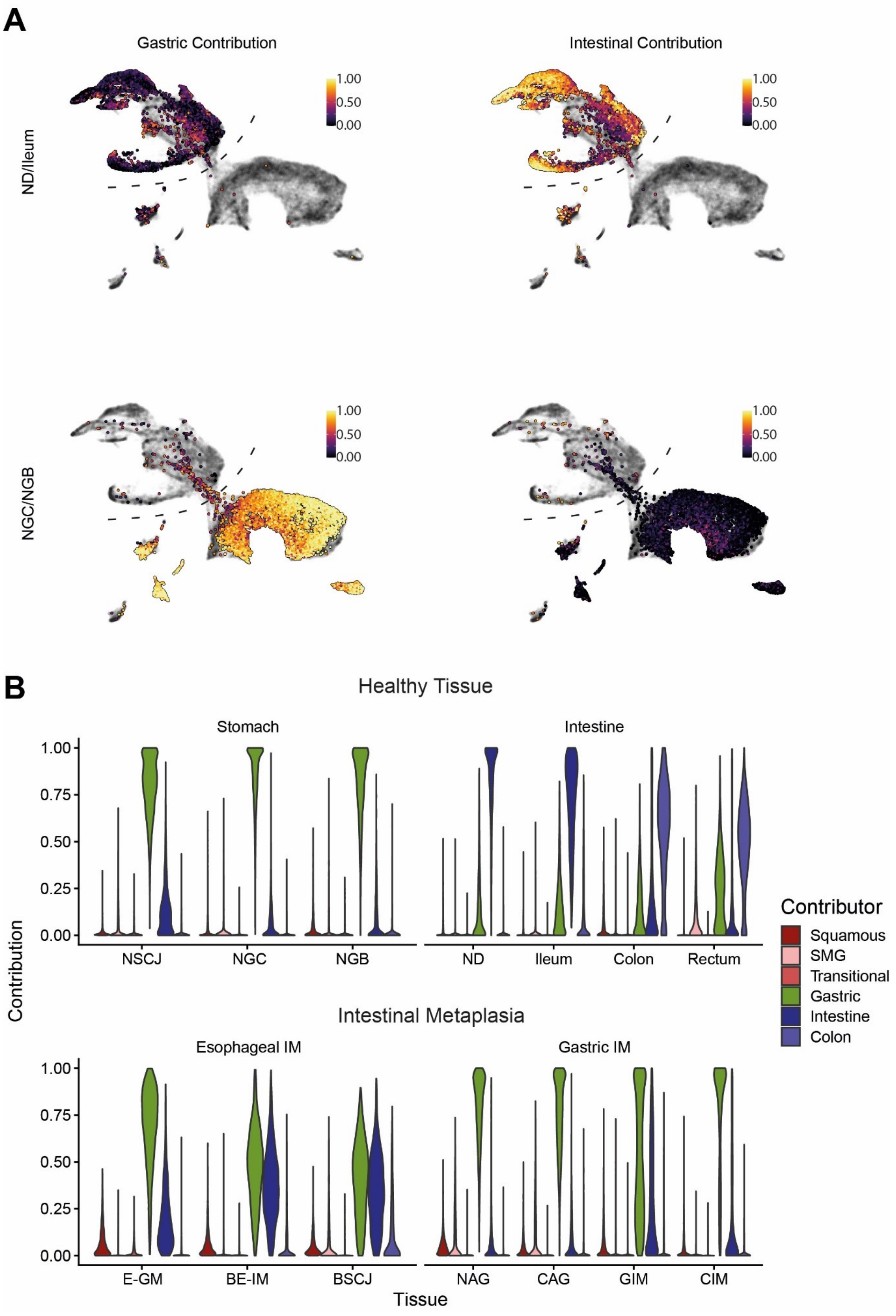
A
Gastric Contribution
Intestinal Contribution
ND/Ileum
NGC/NGB
1.00
0.50
0.00
B
Healthy Tissue
Stomach
Intestine
NSCJ
NGC
NGB
ND
Ileum
Colon
Rectum
Intestinal Metaplasia
Esophageal IM
Gastric IM
E-GM
BE-IM
BSCJ
NAG
CAG
GIM
CIM
Contribution
Tissue
0.00
0.25
0.50
0.75
1.00
Contributor
Squamous
SMG
Transitional
Gastric
Intestine
Colon

Figure S13: Only Gastric and Intestinal phenotypes contribute to Esophageal and Gastric IM cell types

A) UMAP with MuSiC derived contribution of gastric and intestinal phenotypes to individual cells of the small intestine (ND and Ileum, top) and stomach (NGC and NGB).

B) Violin plot of MuSiC derived esophageal (squamous), SMG, Transitional, Gastric, Intestinal and Colonic phenotype contribution to columnar cells from normal tissue types (NSCJ, NGC, NGB, ND, Ileum, Colon, Rectum, top panel) or columnar cells from esophageal and Gastric IM tissue types (E-GM, BE-IM, BSCJ, NAG, CAG, GIM, CIM).

Abbreviation: NSCJ – normal squamo-columnar junction, NGC – normal gastric cardia, NGB – normal gastric body, ND – normal duodenum, BE-IM – Barrett's esophagus, E-GM – gastric metaplasia of esophagus, BSCJ – squamo-columnar junction between NE and BE, CIM – cardia intestinal metaplasia, GIM – gastric intestinal metaplasia, NAG – non-chronic atrophic gastritis, CAG – chronic atrophic gastritis.

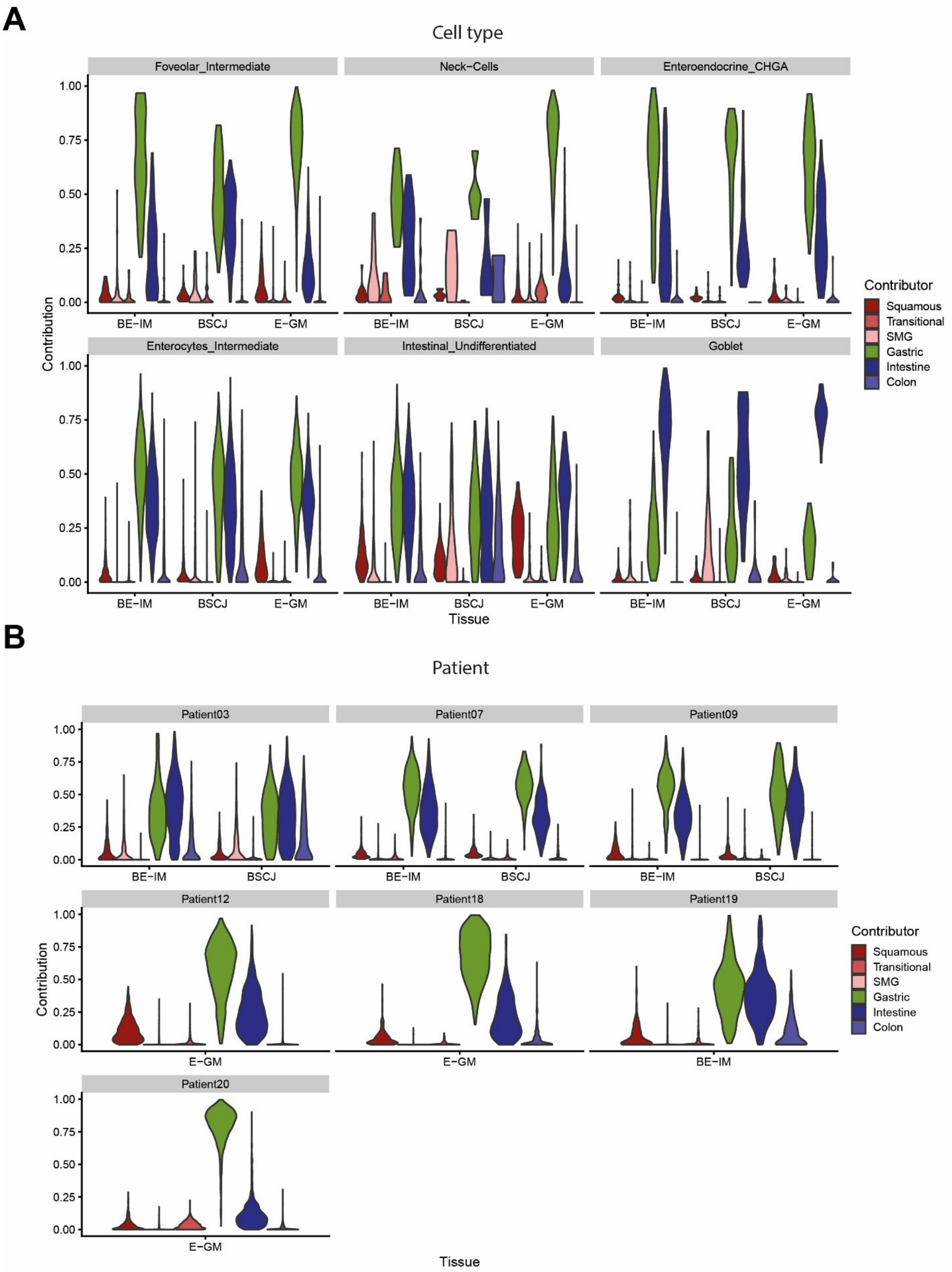
A
Cell type
Foveolar_Intermediate
Neck−Cells
Enteroendocrine_CHGA
Enterocytes_Intermediate
Intestinal_Undifferentiated
Goblet
0.00
0.25
0.50
0.75
1.00
BE−IM
BSCJ
E−GM
Contribution
Tissue
Contributor
Squamous
Transitional
SMG
Gastric
Intestine
Colon
B
Patient
Patient03
Patient07
Patient09
Patient12
Patient18
Patient19
Patient20
Contribution
Tissue

Figure S14: Tissue contribution to esophageal IM samples stratified by cell type and patient

Violin plot of MuSiC derived esophageal (squamous), SMG, Transitional, Gastric, Intestinal and Colonic phenotype contribution to columnar cells from esophageal IM tissue types (E-GM, BE-IM, BSCJ). Data is divided by cell type annotation using *louvain* method (A) or stratified by the patient (B).

Abbreviation: BE-IM – Barrett's esophagus, E-GM – gastric metaplasia of esophagus, BSCJ – squamo-columnar junction between NE and BE.

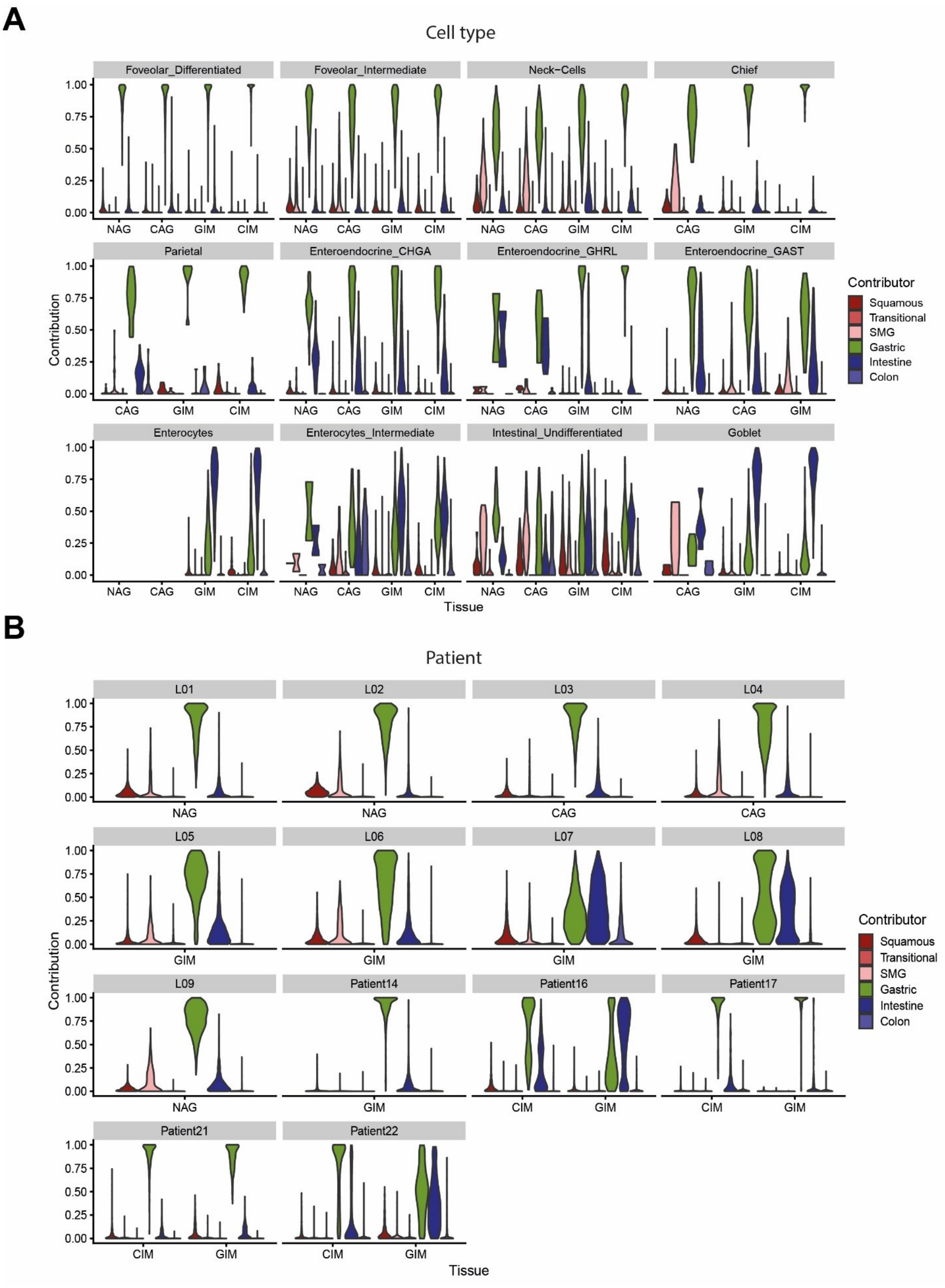
A
Cell type
Foveolar_Differentiated
Foveolar_Intermediate
Neck−Cells
Chief
Parietal
Enteroendocrine_CHGA
Enteroendocrine_GHRL
Enteroendocrine_GAST
Enterocytes
Enterocytes_Intermediate
Intestinal_Undifferentiated
Goblet
1.00
0.75
0.50
0.25
0.00
NAG
CAG
GIM
CIM
Contribution
Tissue
Contributor
Squamous
Transitional
SMG
Gastric
Intestine
Colon
B
Patient
L01
L02
L03
L04
L05
L06
L07
L08
L09
Patient14
Patient16
Patient17
Patient21
Patient22
1.00
0.75
0.50
0.25
0.00
NAG
CAG
GIM
CIM
Contribution
Tissue
Contributor
Squamous
Transitional
SMG
Gastric
Intestine
Colon

Figure S15: Tissue contribution to stomach IM samples stratified by cell type and patient

Violin plot of MuSiC derived esophageal (squamous), SMG, Transitional, Gastric, Intestinal and Colonic phenotype contribution to columnar cells from esophageal IM tissue types (NAG, CAG, GIM and CIM). Data is divided by cell type annotation using *louvain* method (A) or stratified by the patient (B).

Abbreviation: CIM – cardia intestinal metaplasia, GIM – gastric intestinal metaplasia, NAG – non-chronic atrophic gastritis, CAG – chronic atrophic gastritis.

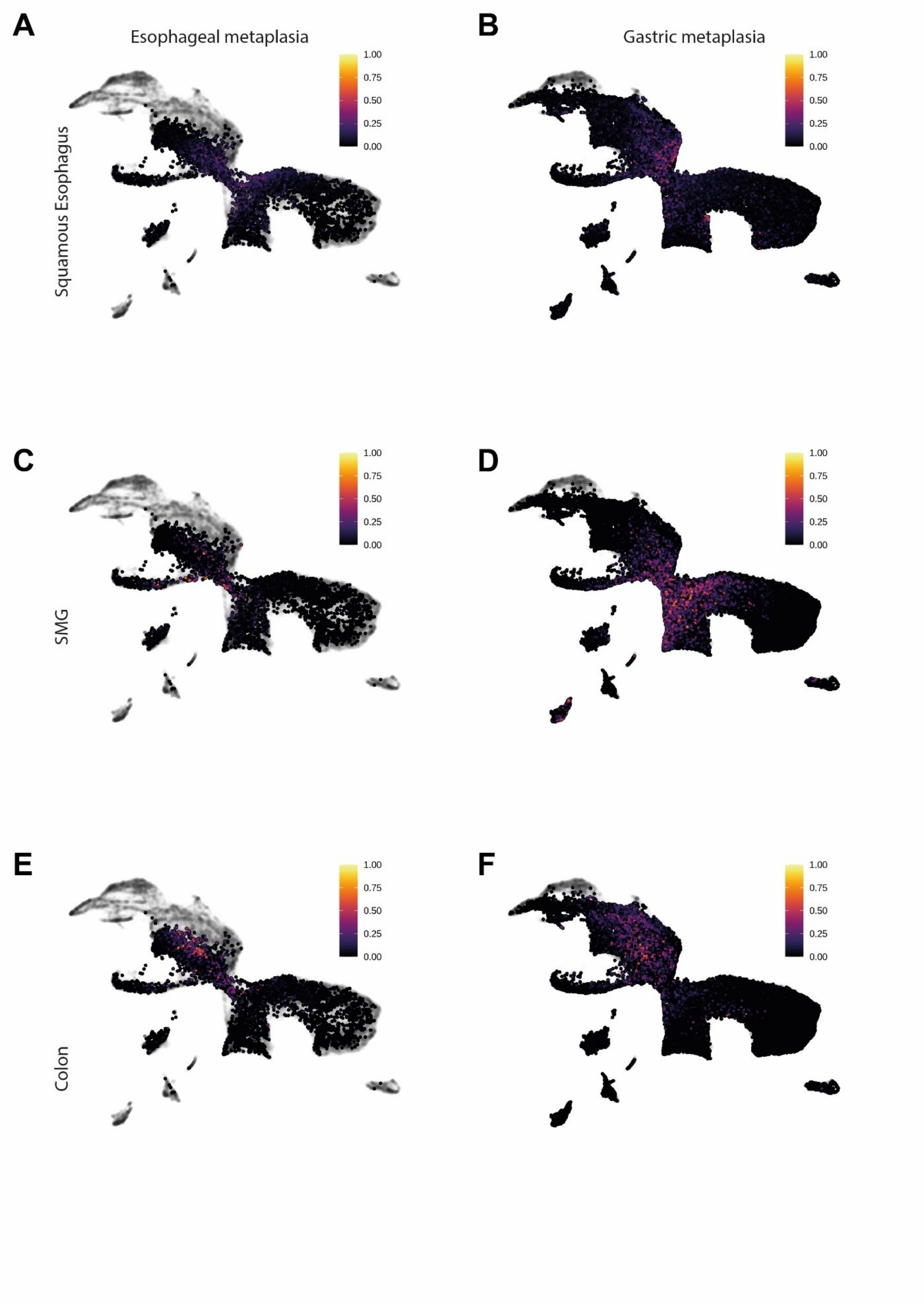
A
Esophageal metaplasia
B
Gastric metaplasia
Squamous Esophagus
C
D
SMG
E
F
Colon
1.00
0.75
0.50
0.25
0.00

Figure S16: Esophageal IM (E-GM and BE-IM) and Gastric IM (NAG, CAG, GIM, CIM) cell do not share features with squamous esophagus, SMG nor Colonic cell types.

UMAP project of columnar cell types from esophageal IM (E-GM and BE-IM) (A, C, E) or gastric IM (NAG, CAG, GIM, CIM (B, D, F) (same coordinates as figure 3A) with MuSiC derived contribution of squamous esophageal (A, B), SGM (C, D) or Colonic (E, F) phenotypes highlighted.

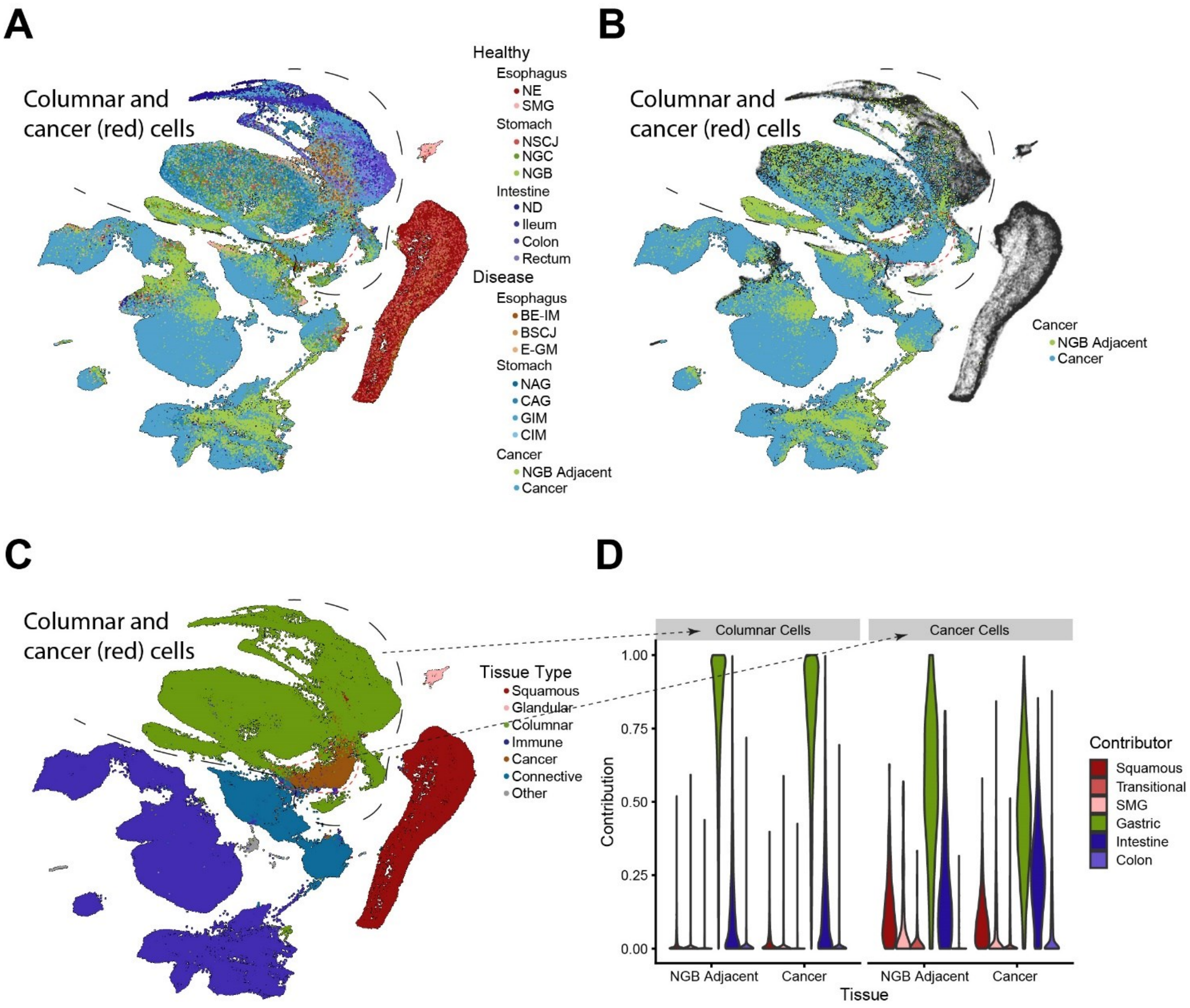
A
Columnar and cancer (red) cells
Healthy
Esophagus
NE
SMG
Stomach
NSCJ
NGC
NGB
Intestine
ND
Ileum
Colon
Rectum
Disease
Esophagus
BE-IM
BSCJ
E-GM
Stomach
NAG
CAG
GIM
CIM
Cancer
NGB Adjacent
Cancer
B
Columnar and cancer (red) cells
Cancer
NGB Adjacent
Cancer
C
Columnar and cancer (red) cells
Tissue Type
Squamous
Glandular
Columnar
Immune
Cancer
Connective
Other
D
Columnar Cells
Cancer Cells
Contribution
1.00
0.75
0.50
0.25
0.00
NGB Adjacent
Cancer
NGB Adjacent
Cancer
Tissue
Contributor
Squamous
Transitional
SMG
Gastric
Intestine
Colon

Figure S17: Gastric cancer cells do not share features with squamous esophagus, SMG nor Colonic cell types.

A) UMAP of all cells from this study with cells obtained from normal gastric samples adjacent to gastric cancer and gastric cancer cells overlaid (Kumar et al. 2022)
B) UMAP of normal gastric samples adjacent to gastric cancer and gastric cancer cells overlaid (Kumar et al. 2022). Only cells from Kumar et al 2022 are presented.
C) UMAP of all cells from this study with cells obtained from normal gastric samples adjacent to gastric cancer and gastric cancer cells overlaid with global tissue type of cells indicated
D) Violin plot of MuSiC-derived esophageal (squamous), SMG, Transitional, Gastric, Intestinal and Colonic phenotype contribution to columnar cells from normal cells adjacent to cancer or columnar cells from gastric cancer samples. Cell from teach tissue type were split into Cancer like cells (brown cells in C) or columnar (non-cancer) like cells (green in C).

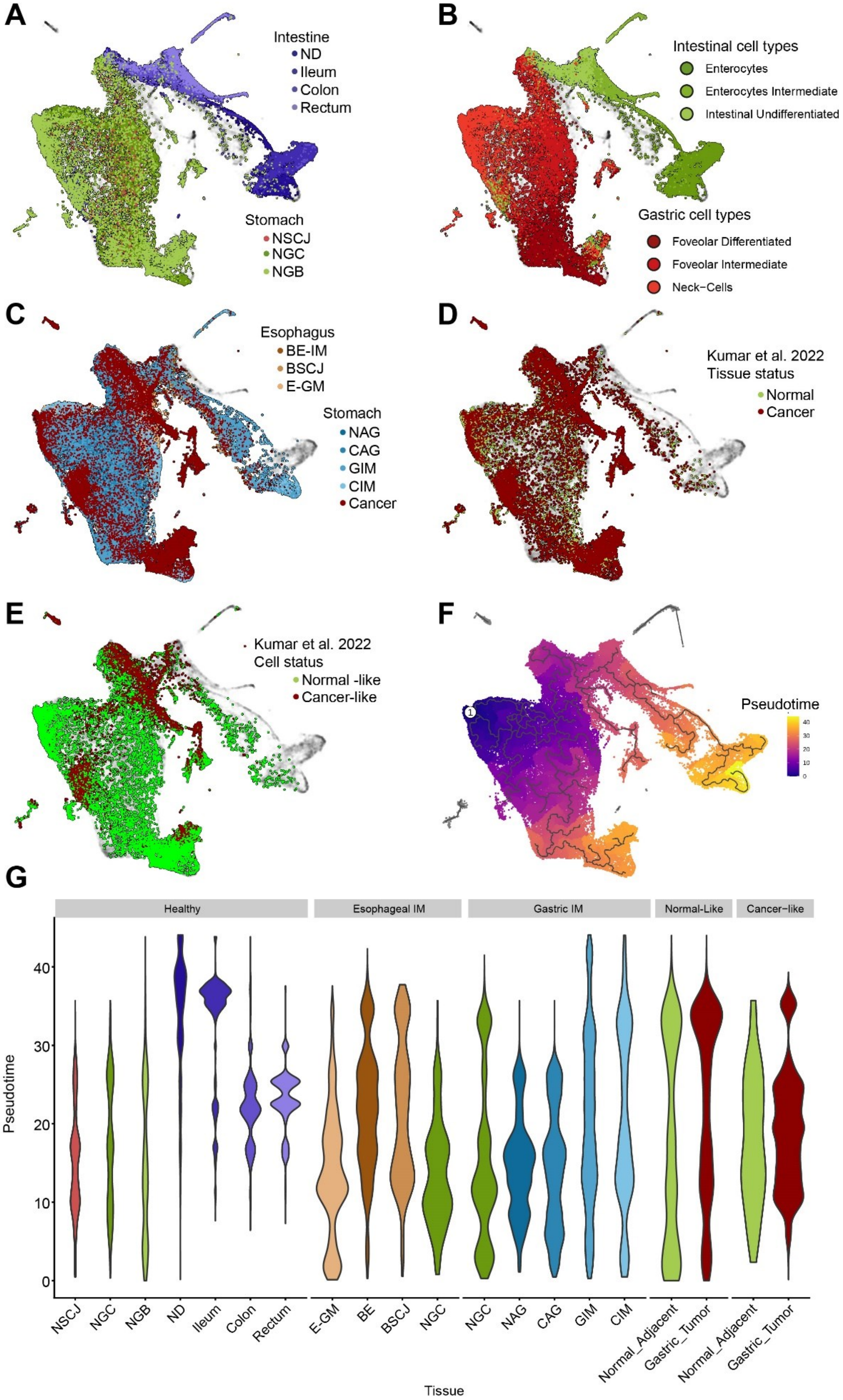

A
Intestine
ND
Ileum
Colon
Rectum
Stomach
NSCJ
NGC
NGB
B
Intestinal cell types
Enterocytes
Enterocytes Intermediate
Intestinal Undifferentiated
Gastric cell types
Foveolar Differentiated
Foveolar Intermediate
Neck−Cells
C
Esophagus
BE-IM
BSCJ
E-GM
Stomach
NAG
CAG
GIM
CIM
Cancer
D
Kumar et al. 2022
Tissue status
Normal
Cancer
E
Kumar et al. 2022
Cell status
Normal -like
Cancer-like
F
Pseudotime
G
Healthy
Esophageal IM
Gastric IM
Normal-Like
Cancer−like
Pseudotime
0
10
20
30
40
NSCJ
NGC
NGB
ND
Ileum
Colon
Rectum
E-GM
BE
BSCJ
NGC
NGC
NAG
CAG
GIM
CIM
Normal_Adjacent
Gastric_Tumor
Normal_Adjacent
Gastric_Tumor
Tissue

Figure S18: ***Monocle3*** **pseudotime analysis places esophageal and gastric intestinal metaplasia cells and gastric cancer cells between normal gastric and intestinal cells.**

A) UMAP of columnar cell types obtained using monocle3 analysis. Only cells from normal tissue types are visualized and tissue types are indicated.
B) UMAP of columnar cell types obtained using monocle3 analysis. Only cells from normal tissue types are visualized and cell types are indicated.
C) UMAP of columnar cell types obtained using monocle3 analysis. Only cells from metaplastic and cancer tissue types are visualized and tissue types are indicated.
D) UMAP of columnar cell types obtained using monocle3 analysis. Only cells from Kumar et al 2022 study are indicated with sample annotation (normal adjacent to or cancer) indicated.
E) UMAP of columnar cell types obtained using monocle3 analysis. Only cells from Kumar et al 2022 study are indicated with cells showing properties of cancer cells indicated.
F) Monocle3 psuedotime trajectory of all columnar cells from normal and metaplastic tissues. Pseudotime trajectory was anchored at the gastric mucous-neck cells, most likely stem-like cells of normal gastric epithelium
G) Violin plot of monocle3 pseudotime trajectory with cells split into individual tissue types and segregated into healthy and esophageal or stomach IM groups. NGC samples were collected from healthy stomach or from endoscopically normal gastric tissue adjacent to BE or Gastric IM (hence it listed 3 time, depending on patient status).

Abbreviation: NSCJ – normal squamo-columnar junction, NGC – normal gastric cardia, NGB – normal gastric body, ND – normal duodenum, BE-IM – Barrett's esophagus, E-GM – gastric metaplasia of esophagus, BSCJ – squamo-columnar junction between NE and BE, CIM – cardia intestinal metaplasia, GIM – gastric intestinal metaplasia, NAG – non-chronic atrophic gastritis, CAG – chronic atrophic gastritis.

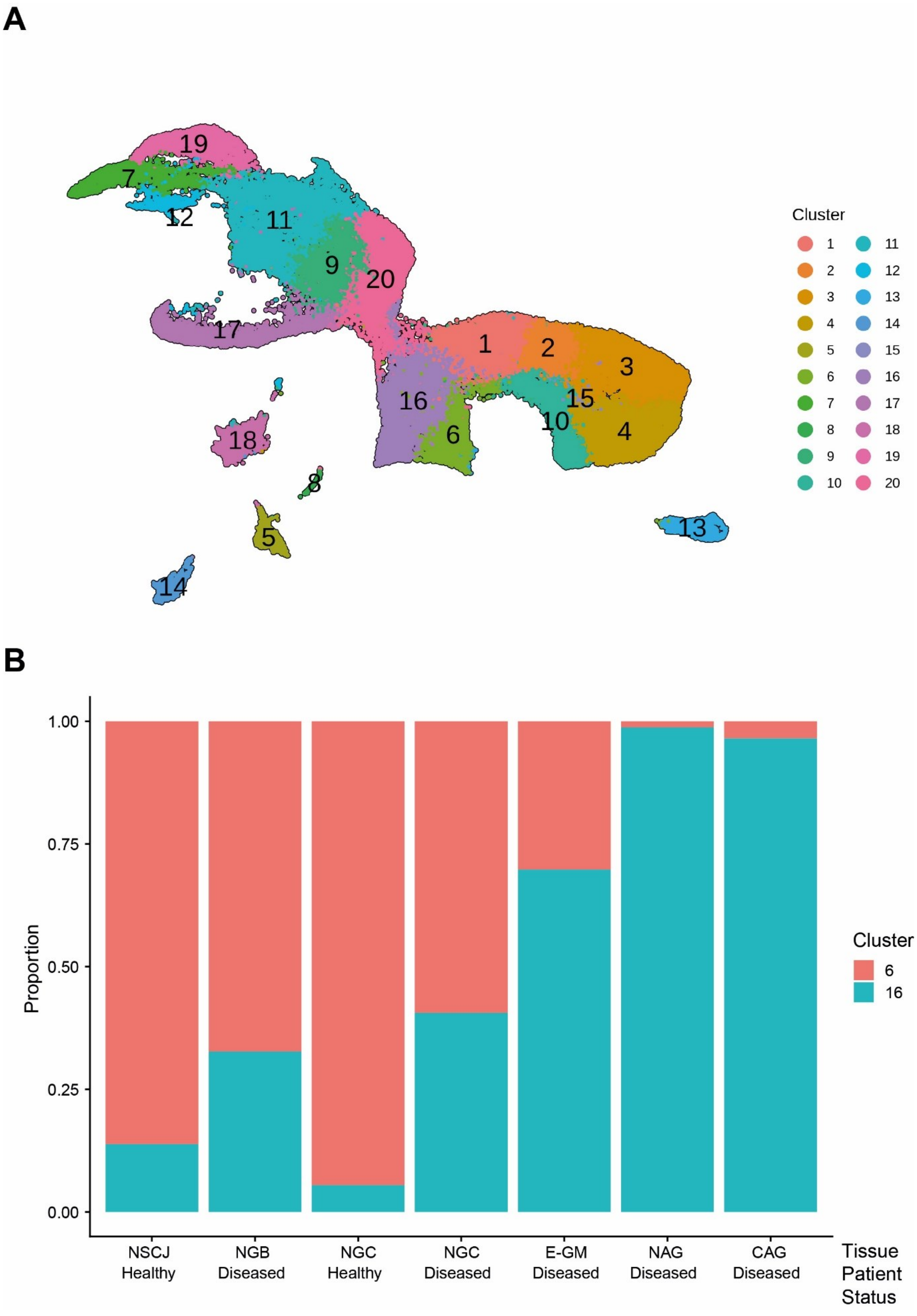
A
19
7
12
11
9
20
17
1
2
3
15
16
10
4
6
18
8
5
13
14
Cluster
1
2
3
4
5
6
7
8
9
10
11
12
13
14
15
16
17
18
19
20
B
Proportion
1.00
0.75
0.50
0.25
0.00
NSCJ Healthy
NGB Diseased
NGC Healthy
NGC Diseased
E-GM Diseased
NAG Diseased
CAG Diseased
Tissue
Patient
Status
Cluster
6
16

Figure S19: Reclustering of columnar cells identifies a new subtype of Neck-like cells.

A) UMAP of all columnar cell types (coordinates as in Fig. 3A) with clusters identified during the reclustering highlighted.
B) Stack bar chart of contribution of neck-like cells that overlap cluster 6 and 16 to individual tissue types. For tissue that patient status could be identified, the tissue was split into healthy and disease states. NGB sample were adjacent to gastric cancer samples.

Abbreviation: NSCJ – normal squamo-columnar junction, NGC – normal gastric cardia, NGB – normal gastric body, E-GM – gastric metaplasia of esophagus, NAG – non-chronic atrophic gastritis, CAG – chronic atrophic gastritis.

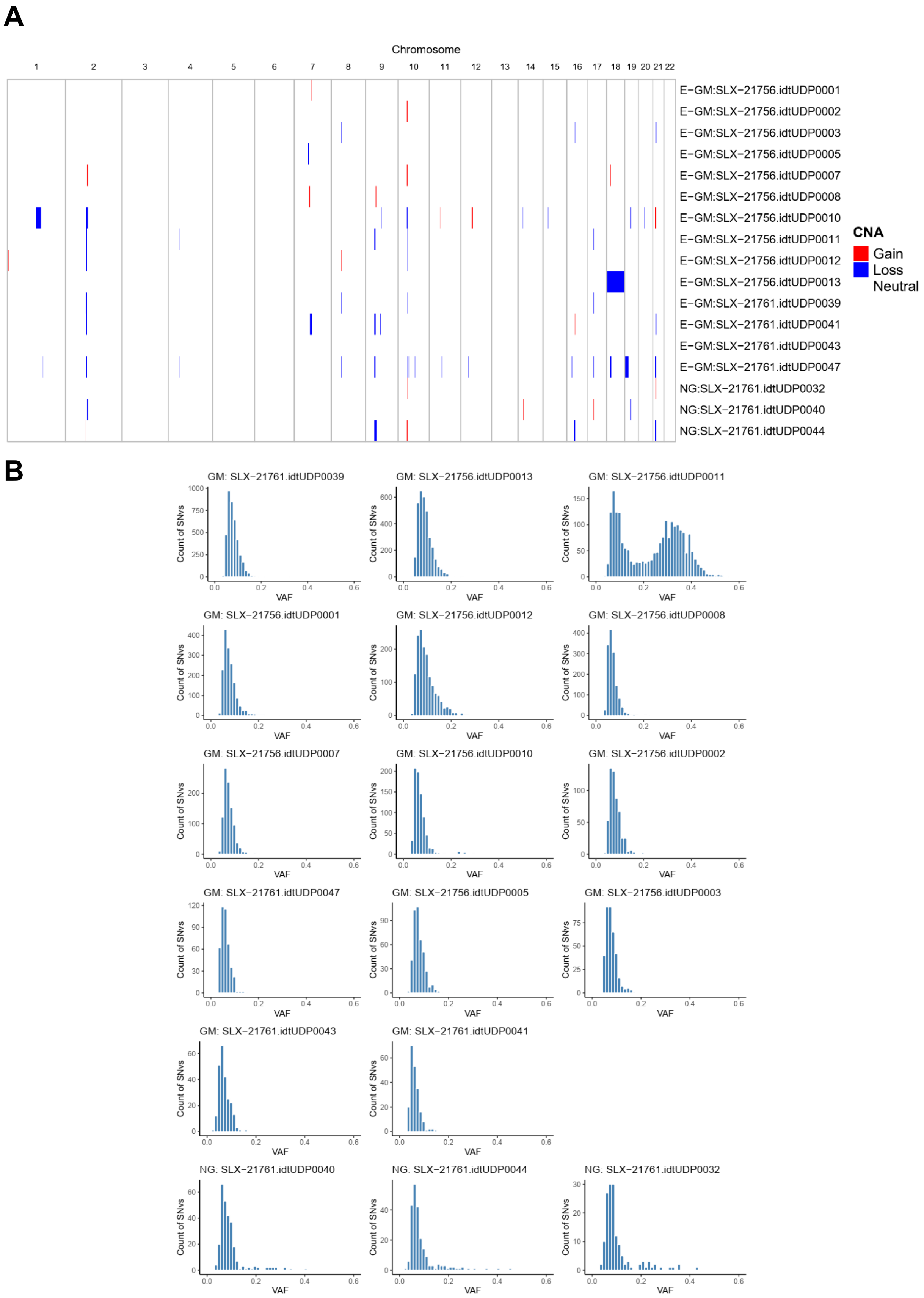
A
Chromosome
1 2 3 4 5 6 7 8 9 10 11 12 13 14 15 16 17 18 19 20 21 22
E−GM:SLX−21756.idtUDP0001
E−GM:SLX−21756.idtUDP0002
E−GM:SLX−21756.idtUDP0003
E−GM:SLX−21756.idtUDP0005
E−GM:SLX−21756.idtUDP0007
E−GM:SLX−21756.idtUDP0008
E−GM:SLX−21756.idtUDP0010
E−GM:SLX−21756.idtUDP0011
E−GM:SLX−21756.idtUDP0012
E−GM:SLX−21756.idtUDP0013
E−GM:SLX−21761.idtUDP0039
E−GM:SLX−21761.idtUDP0041
E−GM:SLX−21761.idtUDP0043
E−GM:SLX−21761.idtUDP0047
NG:SLX−21761.idtUDP0032
NG:SLX−21761.idtUDP0040
NG:SLX−21761.idtUDP0044
CNA
Gain
Loss
Neutral
B
GM: SLX−21761.idtUDP0039
GM: SLX−21756.idtUDP0013
GM: SLX−21756.idtUDP0011
GM: SLX−21756.idtUDP0001
GM: SLX−21756.idtUDP0012
GM: SLX−21756.idtUDP0008
GM: SLX−21756.idtUDP0007
GM: SLX−21756.idtUDP0010
GM: SLX−21756.idtUDP0002
GM: SLX−21761.idtUDP0047
GM: SLX−21756.idtUDP0005
GM: SLX−21756.idtUDP0003
GM: SLX−21761.idtUDP0043
GM: SLX−21761.idtUDP0041
NG: SLX−21761.idtUDP0040
NG: SLX−21761.idtUDP0044
NG: SLX−21761.idtUDP0032
Count of SNVs
VAF

Figure S20: Genetic changes detected in the esophagus with gastric metaplasia

A) Heatmap of copy number alterations identified in the esophagus with gastric metaplasia (E-GM) or normal gastric cardia (NG).
B) Histograms of the distribution of variant allele frequency (VAF) in the esophagus with gastric metaplasia (GM) or normal gastric cardia (NG).

To retain privacy, individual samples are named after their library preparation barcodes.

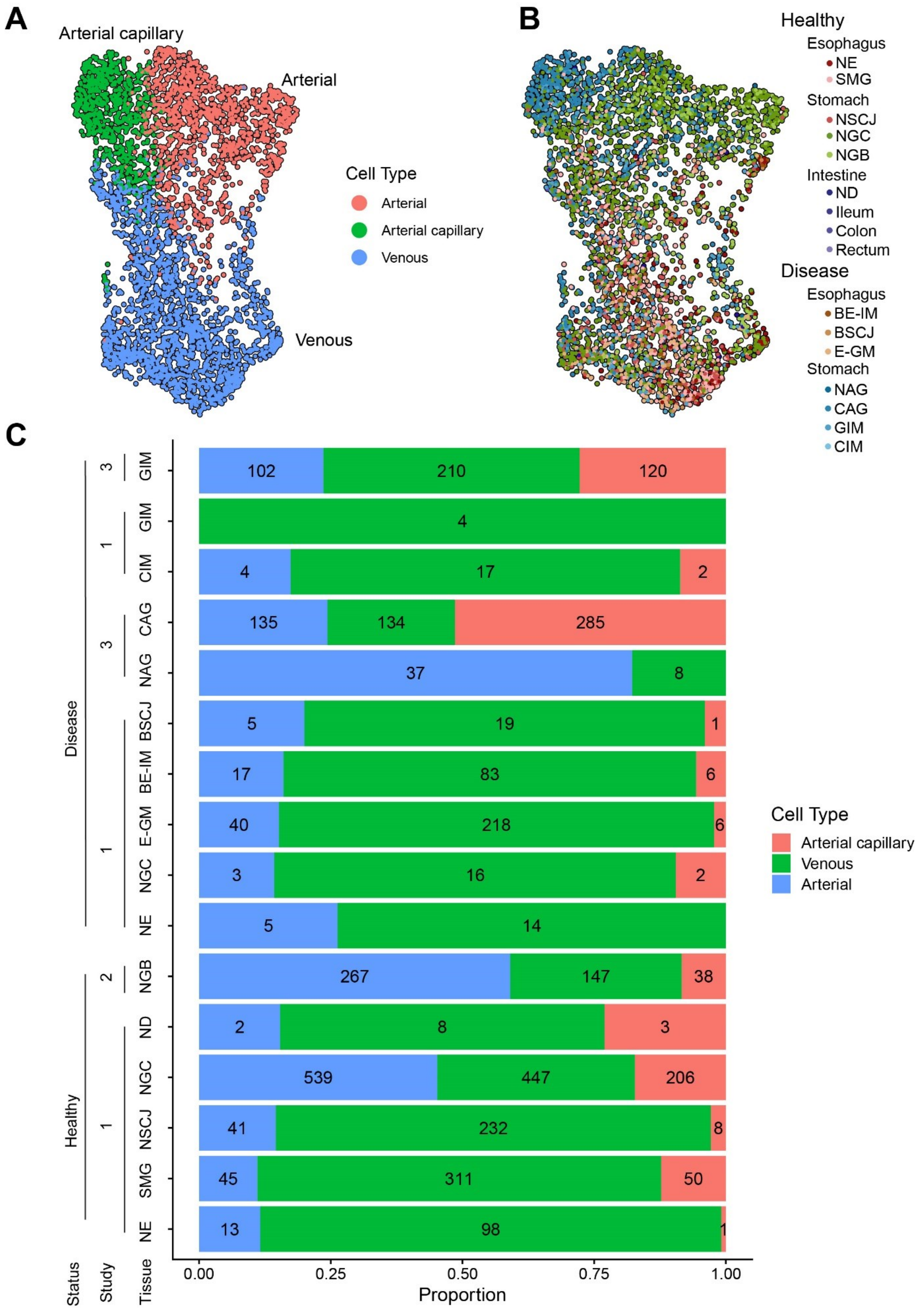

A
Arterial capillary
Arterial
Venous
Cell Type
Arterial
Arterial capillary
Venous
B
Healthy
Esophagus
NE
SMG
Stomach
NSCJ
NGC
NGB
Intestine
ND
Ileum
Colon
Rectum
Disease
Esophagus
BE-IM
BSCJ
E-GM
Stomach
NAG
CAG
GIM
CIM
C
Status
Study
Tissue
Disease
Healthy
GIM 102 210 120
GIM 4
CIM 4 17 2
CAG 135 134 285
NAG 37 8
BSCJ 5 19 1
BE-IM 17 83 6
E-GM 40 218 6
NGC 3 16 2
NE 5 14
NGB 267 147 38
ND 2 8 3
NGC 539 447 206
NSCJ 41 232 8
SMG 45 311 50
NE 13 98 1
0.00 0.25 0.50 0.75 1.00
Proportion
Cell Type
Arterial capillary
Venous
Arterial

Figure S21: Cell classification of endothelial cell types

A) UMAP projection cell types identified within the endothelial cells
B) UMAP projection with tissues of origin for the individual endothelial cell
C) Contribution of endothelial cell types to cell counts from individual tissue types. The tissues originate from three studies: 1 – Nowicki-Osuch and Zhuang et al. and this study, 2 – Sathe et al. 3 – Zhang et al.. For tissue that patient status could be identified, the tissue was split into healthy and disease states. NGB sample were adjacent to gastric cancer samples.

Abbreviation: NE – Normal Esophagus, SMG – esophageal submucosal glands, NSCJ – normal squamo-columnar junction, NGC – normal gastric cardia, NGB – normal gastric body, ND – normal duodenum, BE-IM – Barrett's esophagus, E-GM – gastric metaplasia of esophagus, BSCJ – squamo-columnar junction between NE and BE, CIM – cardia intestinal metaplasia, GIM – gastric intestinal metaplasia, NAG – non-chronic atrophic gastritis, CAG – chronic atrophic gastritis.

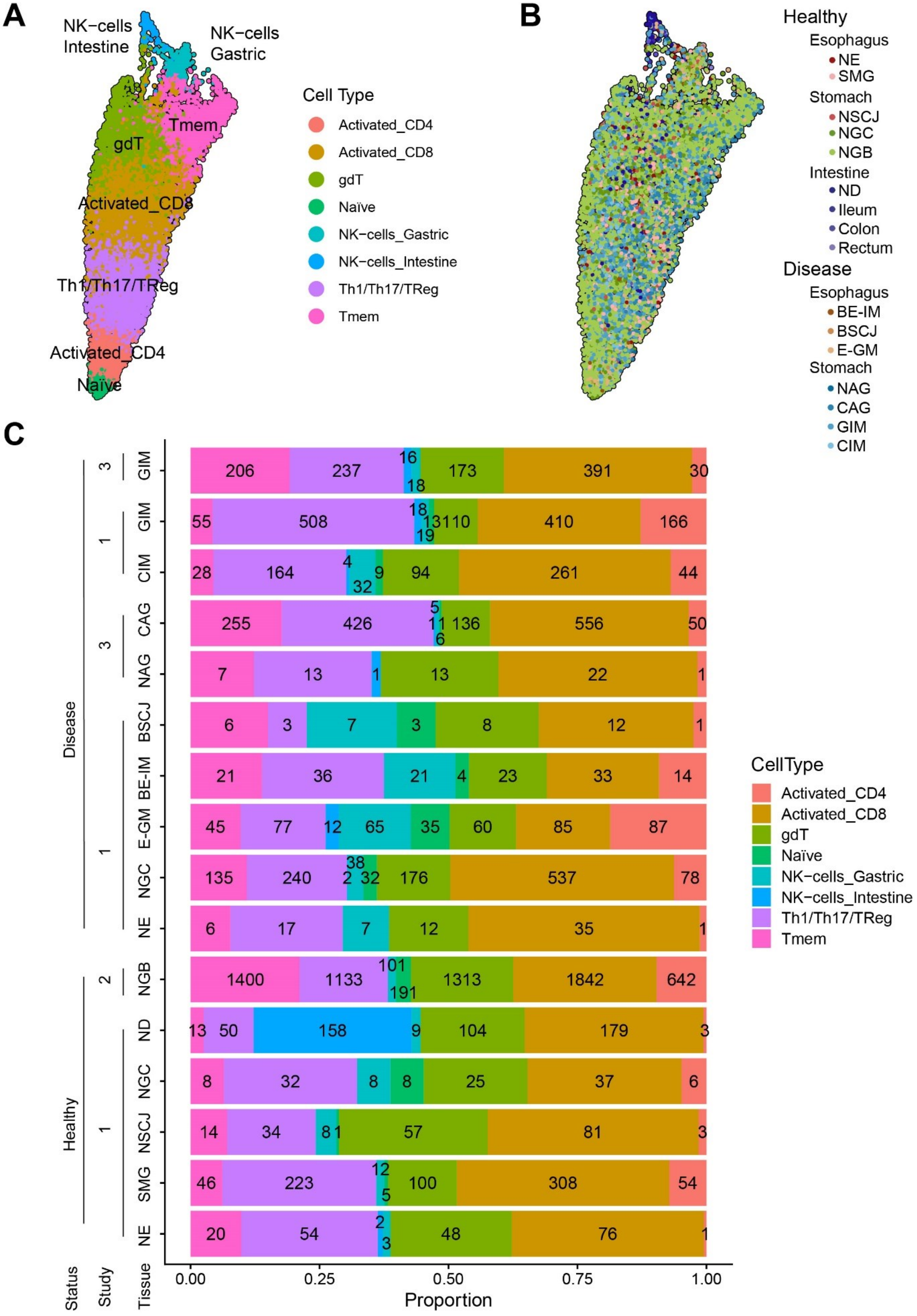
A
NK−cells Intestine
NK−cells Gastric
Tmem
gdT
Activated_CD8
Th1/Th17/TReg
Activated_CD4
Naïve
Cell Type
Activated_CD4
Activated_CD8
gdT
Naïve
NK−cells_Gastric
NK−cells_Intestine
Th1/Th17/TReg
Tmem
B
Healthy
Esophagus
NE
SMG
Stomach
NSCJ
NGC
NGB
Intestine
ND
Ileum
Colon
Rectum
Disease
Esophagus
BE-IM
BSCJ
E-GM
Stomach
NAG
CAG
GIM
CIM
C
Disease
3 GIM 206 237 16 18 173 391 30
1 GIM 55 508 18 13 19 110 410 166
1 CIM 28 164 4 32 9 94 261 44
3 CAG 255 426 5 11 6 136 556 50
3 NAG 7 13 1 13 22 1
1 BSCJ 6 3 7 3 8 12 1
1 BE-IM 21 36 21 4 23 33 14
1 E-GM 45 77 12 65 35 60 85 87
1 NGC 135 240 38 2 32 176 537 78
1 NE 6 17 7 12 35 1
Healthy
2 NGB 1400 1133 101 191 1313 1842 642
1 ND 13 50 158 9 104 179 3
1 NGC 8 32 8 8 25 37 6
1 NSCJ 14 34 8 1 57 81 3
1 SMG 46 223 12 5 100 308 54
1 NE 20 54 2 3 48 76 1
Status
Study
Tissue
0.00
0.25
0.50
0.75
1.00
Proportion
CellType
Activated_CD4
Activated_CD8
gdT
Naïve
NK−cells_Gastric
NK−cells_Intestine
Th1/Th17/TReg
Tmem

Figure S22: Cell classification of T cell types

A) UMAP projection cell types identified within the T cells cluster
B) UMAP projection with tissues of origin for the individual endothelial cell
C) Contribution of endothelial cell types to cell counts from individual tissue types. The tissues originate from three studies: 1 – Nowicki-Osuch and Zhuang et al. and this study, 2 – Sathe et al. 3 – Zhang et al.. For tissue that patient status could be identified, the tissue was split into healthy and disease states. NGB samples were adjacent to gastric cancer samples.

Abbreviation: NE – Normal Esophagus, SMG – esophageal submucosal glands, NSCJ – normal squamo-columnar junction, NGC – normal gastric cardia, NGB – normal gastric body, ND – normal duodenum, BE-IM – Barrett's esophagus, E-GM – gastric metaplasia of esophagus, BSCJ – squamo-columnar junction between NE and BE, CIM – cardia intestinal metaplasia, GIM – gastric intestinal metaplasia, NAG – non-chronic atrophic gastritis, CAG – chronic atrophic gastritis.

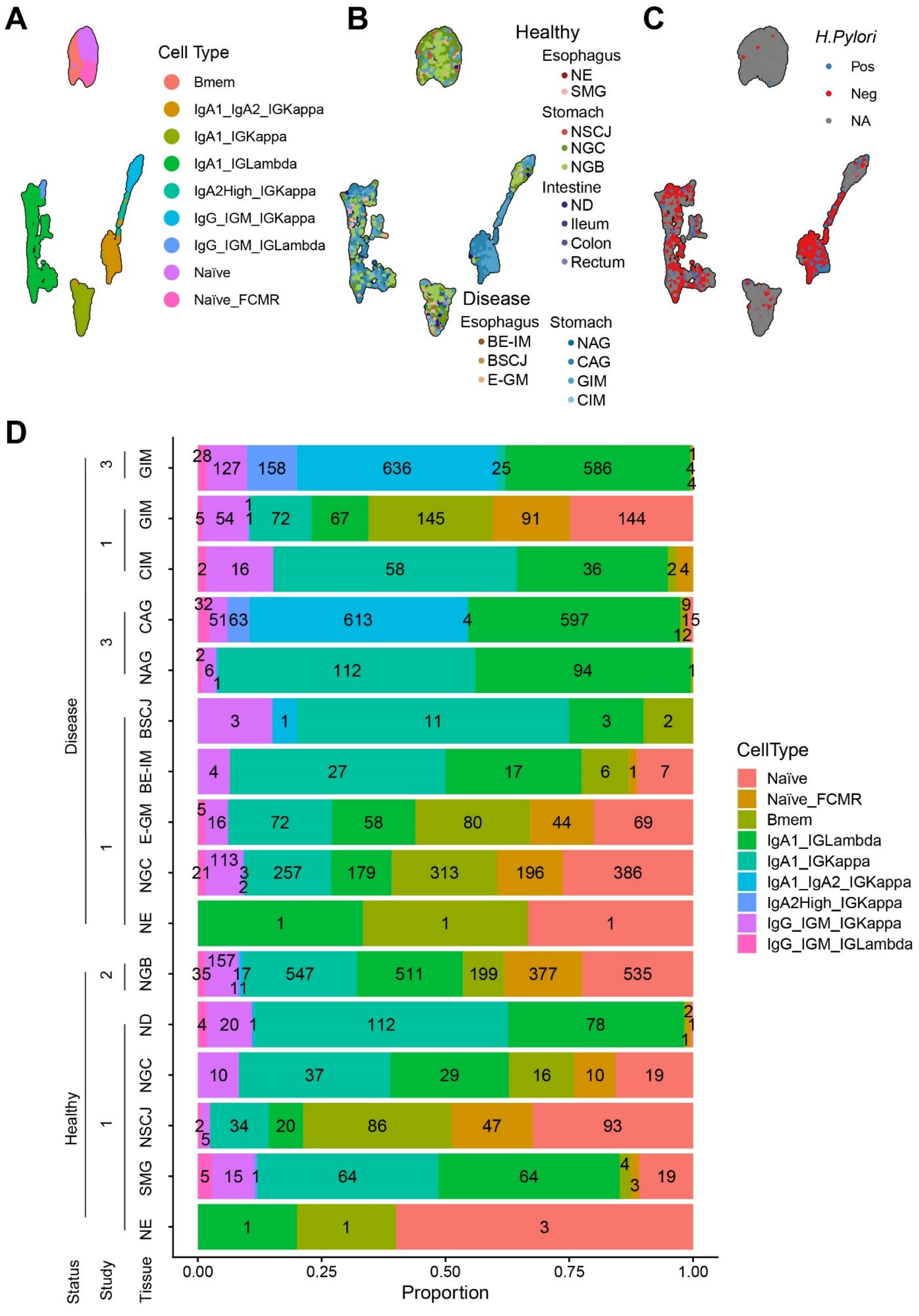

A
Cell Type
Bmem
IgA1_IgA2_IGKappa
IgA1_IGKappa
IgA1_IGLambda
IgA2High_IGKappa
IgG_IGM_IGKappa
IgG_IGM_IGLambda
Naïve
Naïve_FCMR
B
Healthy
Esophagus
NE
SMG
Stomach
NSCJ
NGC
NGB
Intestine
ND
Ileum
Colon
Rectum
Disease
Esophagus
BE-IM
BSCJ
E-GM
Stomach
NAG
CAG
GIM
CIM
C
H.Pylori
Pos
Neg
NA
D
Status
Study
Tissue
Disease
Healthy
Proportion
0.00
0.25
0.50
0.75
1.00
CellType
Naïve
Naïve_FCMR
Bmem
IgA1_IGLambda
IgA1_IGKappa
IgA1_IgA2_IGKappa
IgA2High_IGKappa
IgG_IGM_IGKappa
IgG_IGM_IGLambda

Figure S23: Cell classification of B- and plasma cell types

A) UMAP projection cell types identified within the B- and plasma cell clusters
B) UMAP projection with tissues of origin for the individual endothelial cell
C) UMAP projection with *H.pylori* status (Pos – positive, Neg – negative, NA – data not available) overlay
D) Contribution of endothelial cell types to cell counts from individual tissue types. The tissues originate from three studies: 1 – Nowicki-Osuch and Zhuang et al. and this study, 2 – Sathe et al. 3 – Zhang et al.. For tissue that patient status could be identified, the tissue was split into healthy and disease states. NGB samples were adjacent to gastric cancer samples.

Abbreviation: NE – Normal Esophagus, SMG – esophageal submucosal glands, NSCJ – normal squamo-columnar junction, NGC – normal gastric cardia, NGB – normal gastric body, ND – normal duodenum, BE-IM – Barrett's esophagus, E-GM – gastric metaplasia of esophagus, BSCJ – squamo-columnar junction between NE and BE, CIM – cardia intestinal metaplasia, GIM – gastric intestinal metaplasia, NAG – non-chronic atrophic gastritis, CAG – chronic atrophic gastritis.

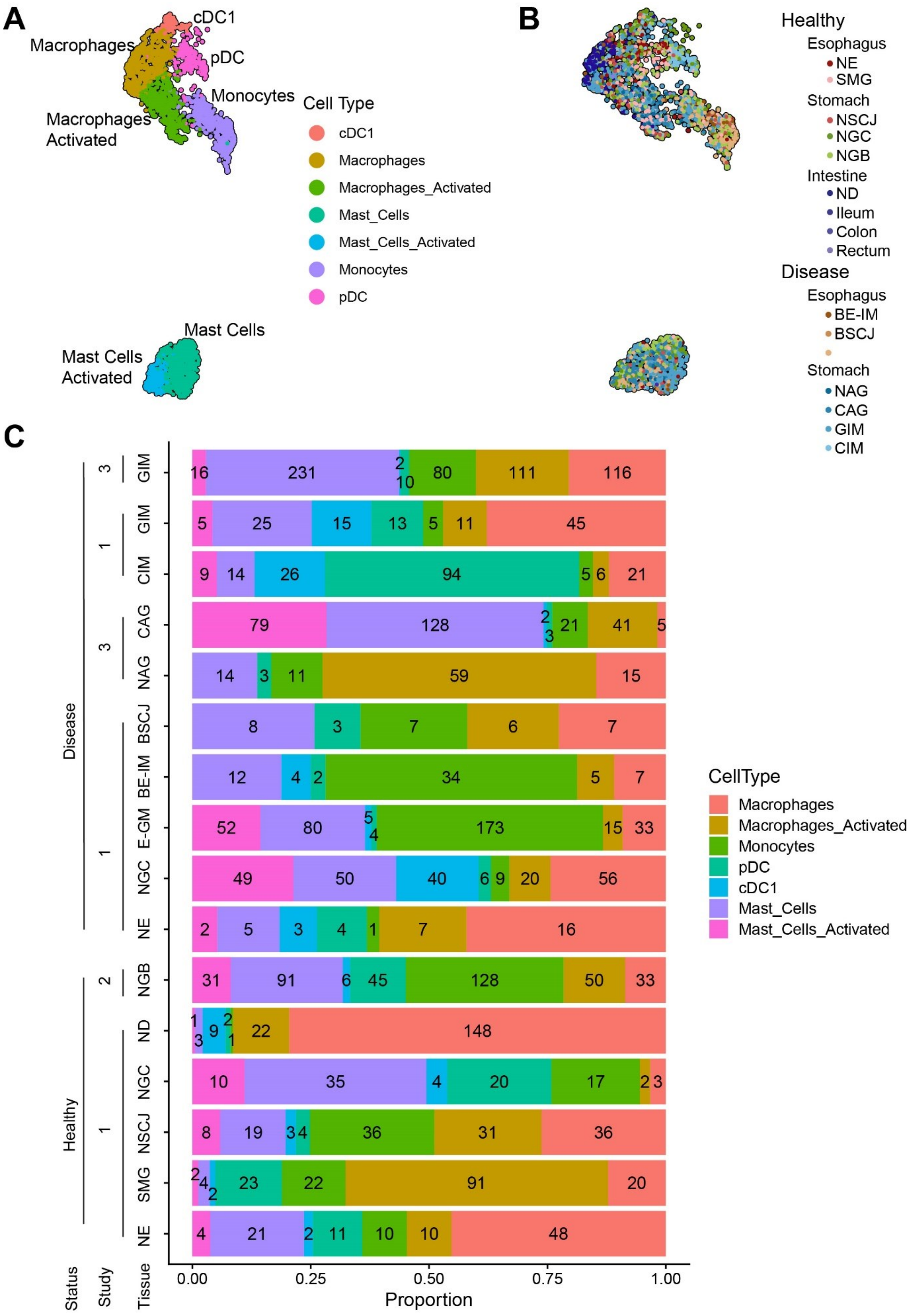
A
cDC1
Macrophages
pDC
Monocytes
Macrophages Activated
Mast Cells
Mast Cells Activated
Cell Type
cDC1
Macrophages
Macrophages_Activated
Mast_Cells
Mast_Cells_Activated
Monocytes
pDC
B
Healthy
Esophagus
NE
SMG
Stomach
NSCJ
NGC
NGB
Intestine
ND
Ileum
Colon
Rectum
Disease
Esophagus
BE-IM
BSCJ
Stomach
NAG
CAG
GIM
CIM
C
Status
Study
Tissue
Disease
Healthy
Proportion
0.00
0.25
0.50
0.75
1.00
CellType
Macrophages
Macrophages_Activated
Monocytes
pDC
cDC1
Mast_Cells
Mast_Cells_Activated

Figure S24: Cell classification of myeloid cell types

A) UMAP projection cell types identified within the myeloid cell clusters
B) UMAP projection with tissues of origin for the individual endothelial cell
C) Contribution of endothelial cell types to cell counts from individual tissue types. The tissues originate from three studies: 1 – Nowicki-Osuch and Zhuang et al. and this study, 2 – Sathe et al. 3 – Zhang et al.. For tissue that patient status could be identified, the tissue was split into healthy and disease states. NGB samples were adjacent to gastric cancer samples.

Abbreviation: NE – Normal Esophagus, SMG – esophageal submucosal glands, NSCJ – normal squamo-columnar junction, NGC – normal gastric cardia, NGB – normal gastric body, ND – normal duodenum, BE-IM – Barrett's esophagus, E-GM – gastric metaplasia of esophagus, BSCJ – squamo-columnar junction between NE and BE, CIM – cardia intestinal metaplasia, GIM – gastric intestinal metaplasia, NAG – non-chronic atrophic gastritis, CAG – chronic atrophic gastritis.

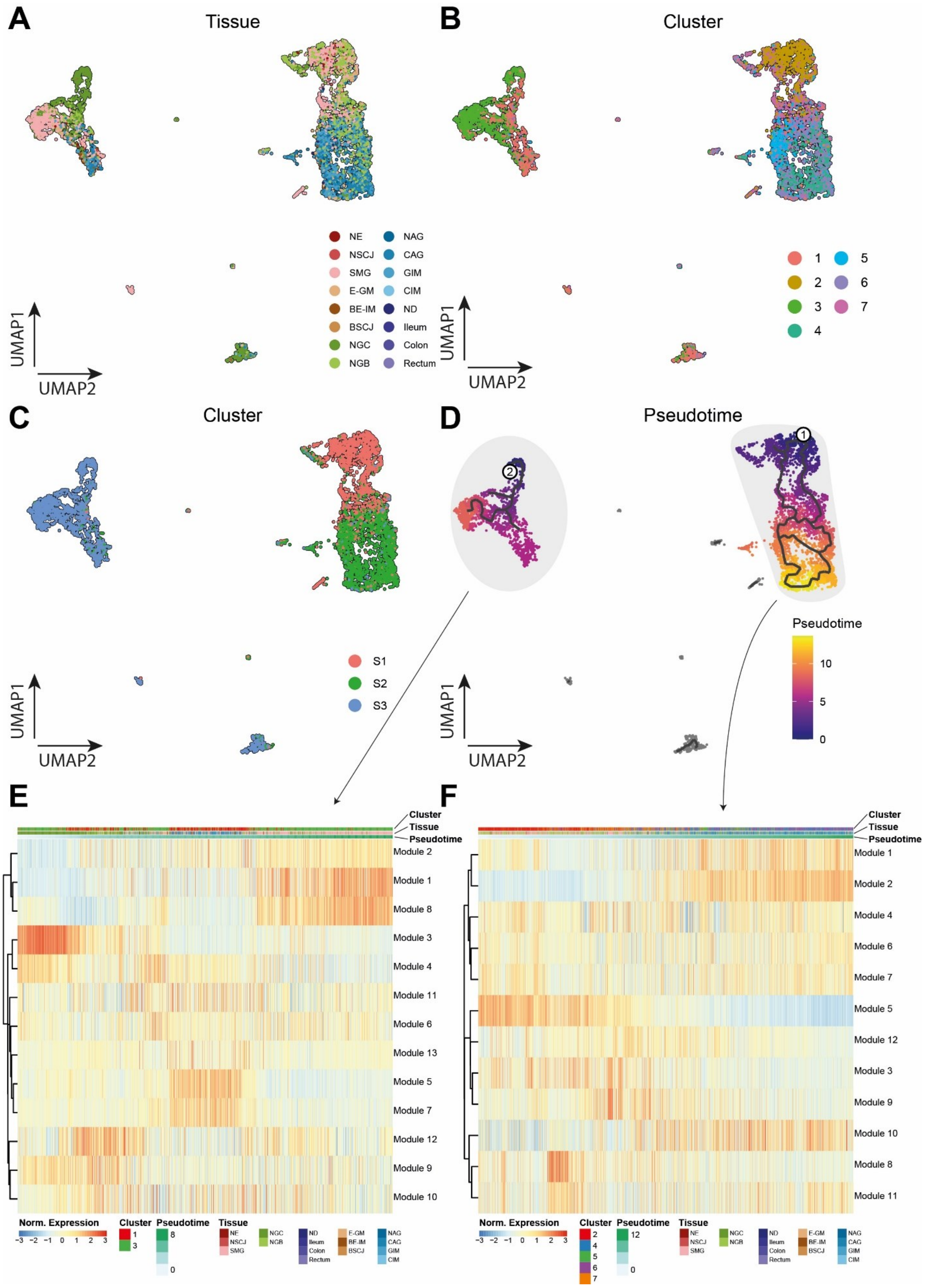

A
Tissue
B
Cluster
NE
NSCJ
SMG
E-GM
BE-IM
BSCJ
NGC
NGB
NAG
CAG
GIM
CIM
ND
Ileum
Colon
Rectum
UMAP1
UMAP2
1
2
3
4
5
6
7
C
Cluster
D
Pseudotime
S1
S2
S3
Pseudotime
10
5
0
E
Cluster
Tissue
Pseudotime
Module 2
Module 1
Module 8
Module 3
Module 4
Module 11
Module 6
Module 13
Module 5
Module 7
Module 12
Module 9
Module 10
Norm. Expression
Cluster
Pseudotime
Tissue
F
Module 1
Module 2
Module 4
Module 6
Module 7
Module 5
Module 12
Module 3
Module 9
Module 10
Module 8
Module 11

Figure S25: ***Monocle3*** **pseudotime time analysis of stromal cell types**

A) UMAP of fibroblasts-like cells (cluster 13 on figure S1) with contribution of individual tissue types highlighted.
B) UMAP of fibroblasts-like cells (cluster 13 on figure S1) with fibroblast specific clusters highlighted.
C) UMAP of fibroblasts-like cells (cluster 13 on figure S1) with manually annotated cell types derived from Davidson et al.
D) UMAP of fibroblasts-like cells (cluster 13 on figure S1) with *monocle3* pseudotime overlay. The trajectories were fixed at the extreme of S1 cell population (for S1-S2 trajectory) and at the normal gastric terminus of S3 trajectory.
E) Heatmap of normalized expression values for gene modules identified in the S3 trajectory. The cells are ordered by pseudotime value from D.
F) Heatmap of normalized expression values for gene modules identified in the S1-S2 trajectory. The cells are ordered by pseudotime value from D.

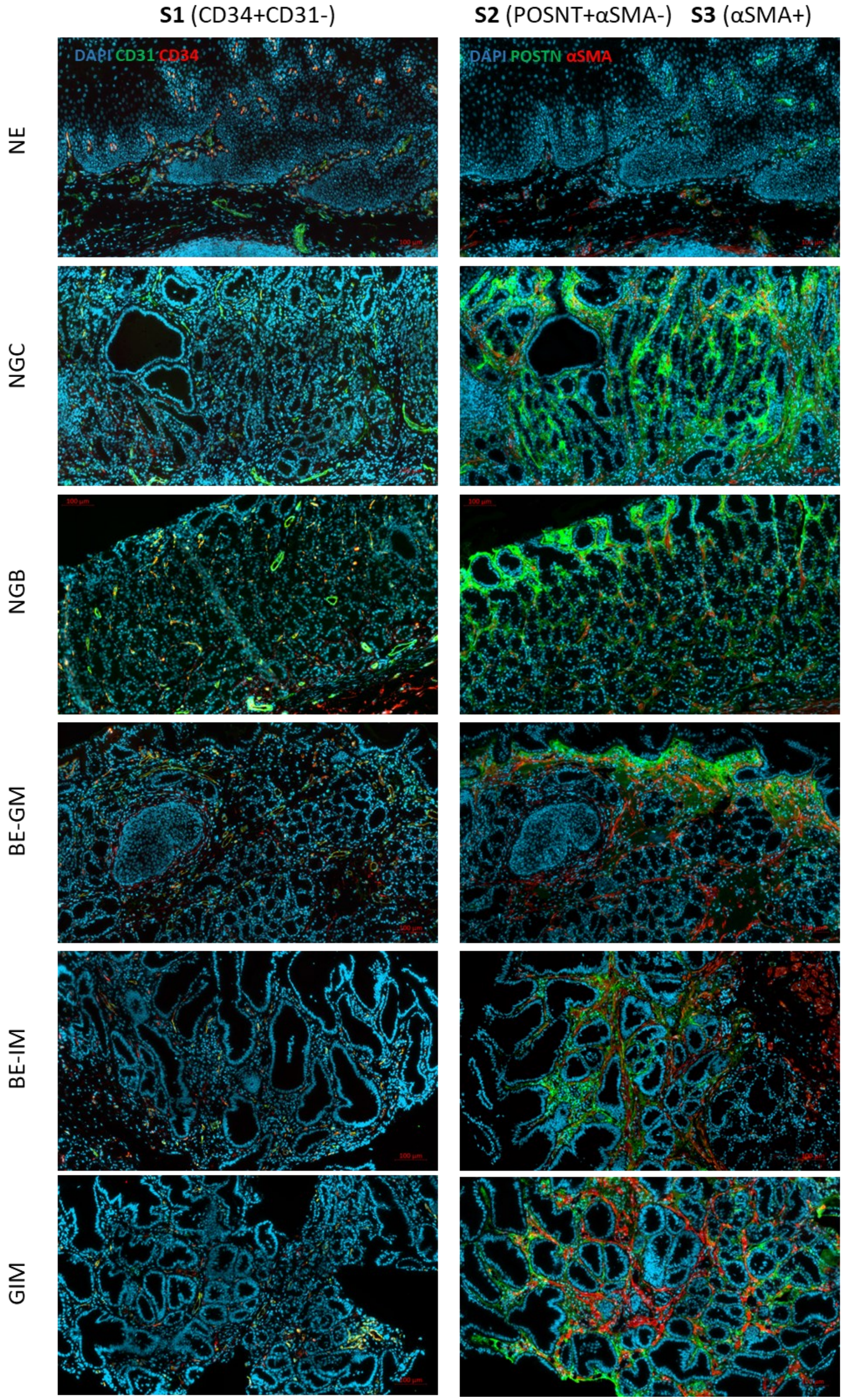
S1 (CD34+CD31-)
S2 (POSNT+αSMA-) S3 (αSMA+)
DAPI CD31 CD34
DAPI POSTN αSMA
NE
NGC
NGB
BE-GM
BE-IM
GIM

Figure S26: Immunofluorescent staining of S1 (CD34+CD31-), S2 (POSTN+aSMA-) and S3 (aSMA+) fibroblasts.

NE, NGC and NGB samples for immunofluorescence were from disease-free organ donors. NGC was defined as the immediate 3 mm long mucosa area adjacent to the Z-line; NGB was defined as the gastric mucosa that 3 to 5 cm away from the Z-line. NE – normal esophagus, NGC – normal gastric cardia, NGB – normal gastric body, E-GM – esophagus with gastric metaplasia, BE-IM – Barrett's esophagus with intestinal metaplasia, GIM – gastric intestinal metaplasia

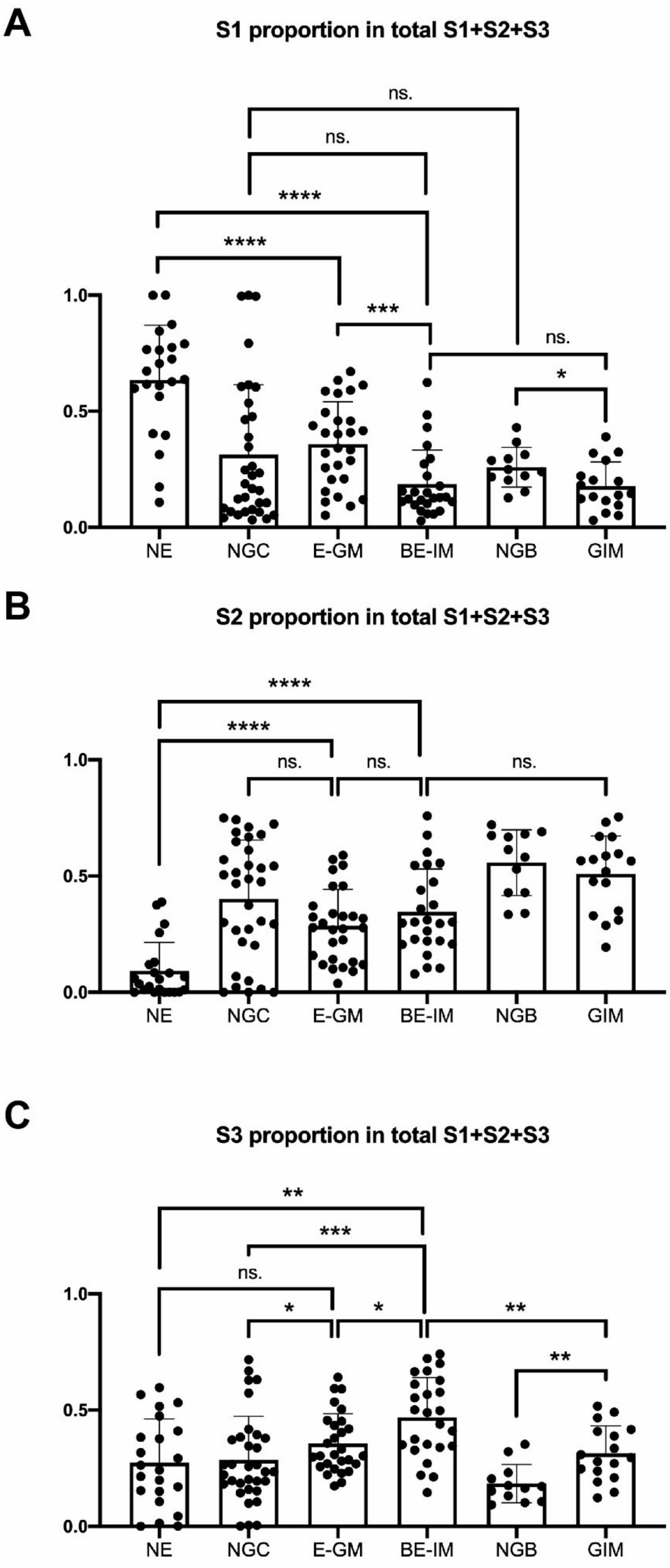
A
S1 proportion in total S1+S2+S3
ns.
ns.
****
****
***
ns.
*
1.0
0.5
0.0
NE
NGC
E-GM
BE-IM
NGB
GIM
B
S2 proportion in total S1+S2+S3
****
****
ns.
ns.
ns.
1.0
0.5
0.0
NE
NGC
E-GM
BE-IM
NGB
GIM
C
S3 proportion in total S1+S2+S3
**
***
ns.
*
*
**
**
1.0
0.5
0.0
NE
NGC
E-GM
BE-IM
NGB
GIM

Figure S27: Proportion of each subtype in total S1/S2/S3 fibroblasts derived from immunofluorescent staining of E-GM, BE-IM and GIM.

A) Proportion of S1 populations in total S1/S2/S3 count of fibroblasts
B) Proportion of S2 populations in total S1/S2/S3 count of fibroblasts
C) Proportion of S3 populations in total S1/S2/S3 count of fibroblasts

Statistical analysis was performed using Mann Whitney two-tailed test: ****: $p < 0.0001$; ***: $p < 0.001$; **: $p < 0.01$; *: $p < 0.05$; n.s. – not significant

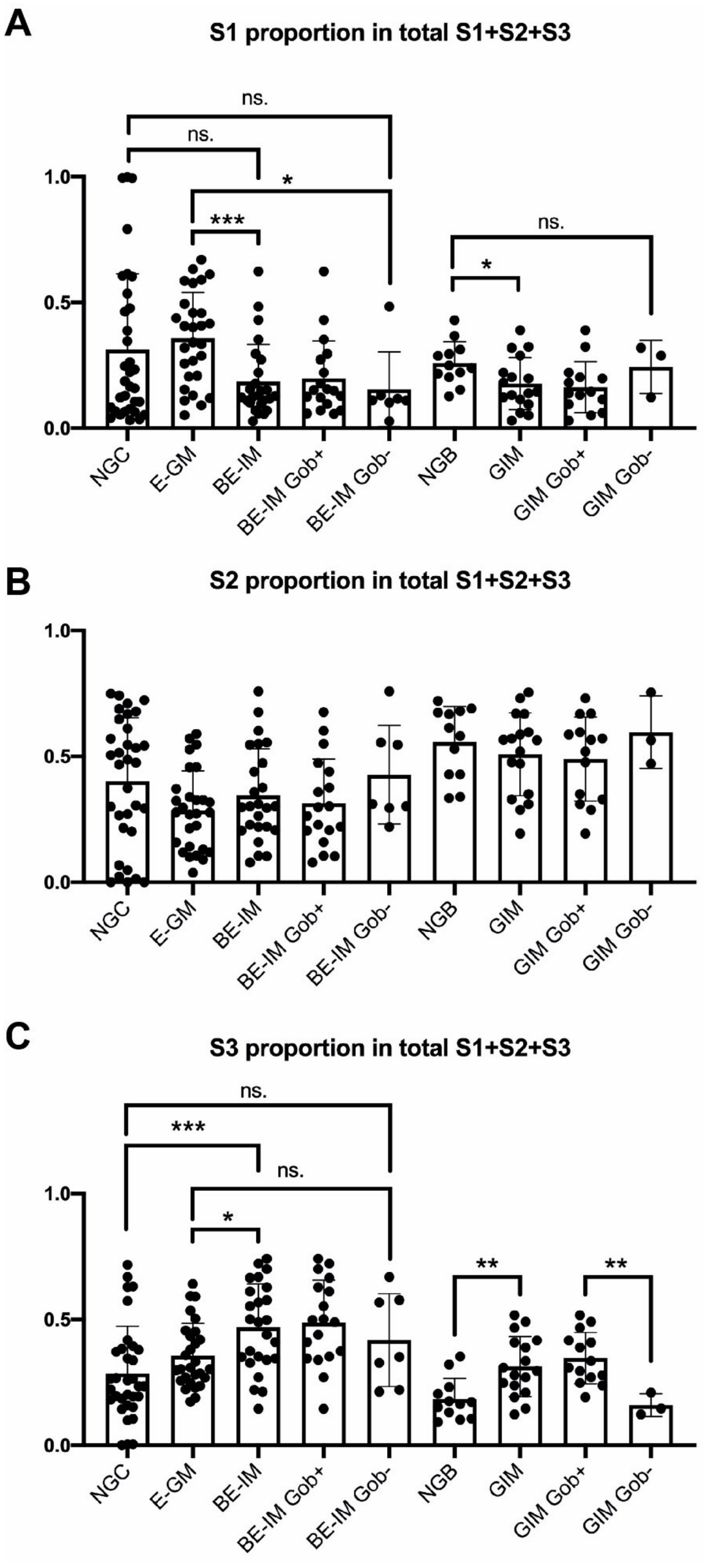
A
S1 proportion in total S1+S2+S3
ns.
ns.
*
***
ns.
*
1.0
0.5
0.0
NGC
E-GM
BE-IM
BE-IM Gob+
BE-IM Gob-
NGB
GIM
GIM Gob+
GIM Gob-
B
S2 proportion in total S1+S2+S3
1.0
0.5
0.0
NGC
E-GM
BE-IM
BE-IM Gob+
BE-IM Gob-
NGB
GIM
GIM Gob+
GIM Gob-
C
S3 proportion in total S1+S2+S3
ns.
***
ns.
*
**
**
1.0
0.5
0.0
NGC
E-GM
BE-IM
BE-IM Gob+
BE-IM Gob-
NGB
GIM
GIM Gob+
GIM Gob-

Figure S28: Proportion of each subtype in total S1/S2/S3 fibroblasts derived from immunofluorescent staining of E-GM, BE-IM, and GIM in the context of goblet cells

A) Proportion of S1 populations in total S1/S2/S3 count of fibroblasts
B) Proportion of S2 populations in total S1/S2/S3 count of fibroblasts
C) Proportion of S3 populations in total S1/S2/S3 count of fibroblasts

BE-IM Gob+: biopsies from BE-IM segment, in which goblet cells are present; BE-IM Gob-: biopsies from BE-IM segment, in which goblet cells are absent; GIM Gob+: biopsies from GIM patient, in which goblet cells are present; GIM Gob-: biopsies from GIM patient, in which goblet cells are absent. Statistical analysis was performed using Mann Whitney two-tailed test: ****: $p < 0.0001$; ***: $p < 0.001$; **: $p < 0.01$; *: $p < 0.05$; n.s. not significant
